# Three ancient documents solve the jigsaw of the parchment purple spot deterioration and validate the microbial succession model

**DOI:** 10.1038/s41598-018-37651-y

**Published:** 2019-02-07

**Authors:** Luciana Migliore, Nicoletta Perini, Fulvio Mercuri, Silvia Orlanducci, Alessandro Rubechini, Maria Cristina Thaller

**Affiliations:** 1 Department of Biology, Tor Vergata University, Rome, Italy; 2 Department of Industrial Engineering, Tor Vergata University, Rome, Italy; 3 Department of Chemical Science and Technology, Tor Vergata University, Rome, Italy; 4 Vatican Secret Archives, Vatican City, Vatican City State

## Abstract

The preservation of cultural heritage is one of the major challenges of today’s society. Parchments, a semi-solid matrix of collagen produced from animal skin, are a significant part of the cultural heritage, being used as writing material since ancient times. Due to their animal origin, parchments easily undergo biodeterioration: the most common biological damage is characterized by isolated or coalescent purple spots, that often lead to the detachment of the superficial layer and the consequent loss of written content. Although many parchments with purple spot biodegradative features were studied, no common causative agent had been identified so far. In a previous study a successional model has been proposed, basing on the multidisciplinary analysis of damaged versus undamaged samples from a moderately damaged document. Although no specific sequences were observed, the results pointed to *Halobacterium salinarum* as the starting actor of the succession. In this study, to further investigate this topic, three dramatically damaged parchments were analysed; belonging to a collection archived as *Faldone Patrizi A 19*, and dated back XVI-XVII century A.D. With the same multidisciplinary approach, the Next Generation Sequencing (NGS, Illumina platform) revealed DNA sequences belonging to *Halobacterium salinarum*; the RAMAN spectroscopy identified the pigment within the purple spots as haloarchaeal bacterioruberin and bacteriorhodopsine, and the LTA technique quantified the extremely damaged collagen structures through the entire parchments, due to the biological attack to the parchment frame structures. These results allowed to propose a model of the progressive degradation pattern of the parchment collagen. Overall, these data validate a multi-phase microbial succession model. This demonstration is pivotal to possible new restoration strategies, important for a huge number of ancient documents.

## Introduction

The most used writing support in ancient times was parchment, a semi-solid matrix of collagen produced from animal skin (*i*.*e*. sheep or goats). Ancient parchments commonly undergo a biological damage characterized by isolated or coalescent purple spots^1,2^. In correspondence of purple spots, often the superficial layer of the parchment is clearly damaged and detached, leading to the irreversible loss of its historical written content^3,4^.

The preservation of historical parchments is one of the major challenges of today’s society, needing an updated technical approach. In a recent paper^1^ we proposed an integrated approach to investigate the purple spot damage of ancient parchments. The Next-Generation Sequencing (NGS, 454-pyrosequencing) was successfully used for the first time to describe and identify colonizers of the damaged parchments. Raman spectroscopy, already used to provide the chemical identification of pigments in cultural heritage, was applied to rapidly identify the pigments in the purple spots without altering samples structure. Lastly, the novel Light Transmission Analysis (LTA) technique, was used to quantify the structural damage suffered by the native collagen in damaged areas. This interdisciplinary approach was applied on a roll dated back 1244 A.D., belonging to the oldest collection of the Secret Vatican Archives (*A*.*A*. *Arm*. *I-XVIII 3328*; Fondo *“Archivum Arcis”*), and produced interesting results by measuring the differences between purple damaged and uncoloured undamaged areas of the parchment. The NGS metagenomic analysis revealed different prokaryotic communities in the damaged and undamaged areas of the document, with Pseudonocardiales mostly present in the undamaged areas, and Gamma-Proteobacteria (mainly *Vibrio*) exclusively present in the damaged ones. Ubiquitous and environmental bacteria were observed in both sets of samples. In the purple spots, the Raman spectroscopy identified rhodopsin-like pigments: purple transmembrane protein containing retinal produced mainly by *Halobacteria* whose genetic traces, however, were not found in the parchment. The Light Transmission Analysis technique bared the collagen structural damage in the purple areas: bacteria cause the thinning out of the interfibers collagen texture and some deterioration of the fibre sheath, the more chemically stable kind of collagen. Basing on these results, a biodeterioration model was hypothesized and proposed. This model consists in a microbial succession, regarded as the putative responsible of the purple spot damage of parchments. During an ecological succession, the community structure of the site changes over time, so that species substitution takes place. The succession starts by the activity (and growth) of pioneer species. The process occurs in every kind of ecosystems and its course depends on the quality and quantity of available resources. The species substitution often depends on a ‘facilitation’ process as the system modification introduced by the activity of the colonizers make the environment more favourable to species other than the colonizers themselves. Successions can be autotrophic or heterotrophic, according to the photosynthesis/respiration ratio^5^. Heterotrophic successions must take advantage on the energy/material contained in the system and their ‘aim’ is to mineralize the nutrient content in the framework of biogeochemical cycles.

The ecological succession putative responsible of the purple spot damage of parchments still remains unsolved in some points. The most important and still lacking piece of this jigsaw puzzle of parchment biodegradation being the recovery of the DNA traces of haloarchaea, supposed to be the ‘pioneer species’, on the base of the presence of retinal residues and bacterioruberin.

**Figure 1:**
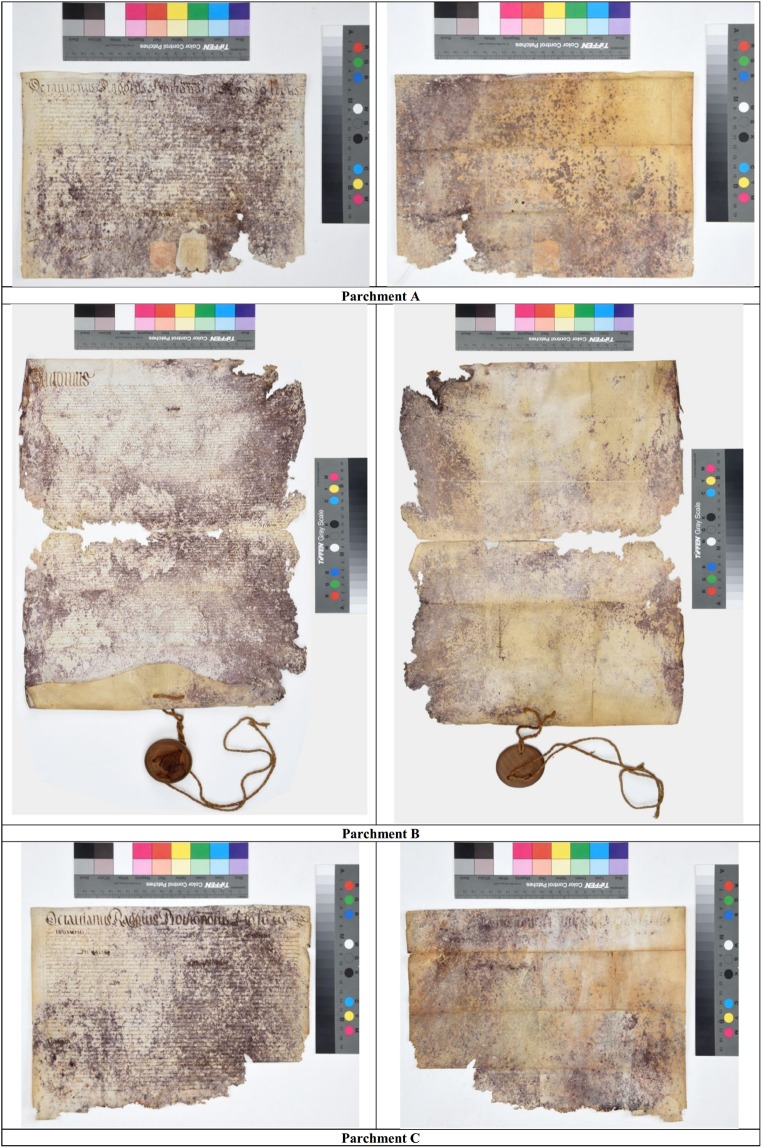
Parchments of the Faldone Patrizi A 19, from the Vatican Secret Archives. The three parchments were identified as (**A–C**). (**A**) Document named ‘Octavianus Raggius Protonotarius Apostolicus’ and dated 1640 A.D, XVII year of pontificate of Pope Urbanus VIII; (**B**) Document named ‘Antonius…’ and dated about 1510 A.D., VII year of pontificate of Pope Julius II; (**C** Document named ‘Octavianus Raggius Protonotarius…’ and dated 1639 A.D., XVI year of pontificate of Pope Urbanus VIII.

The aims of this study were: to solve the jigsaw puzzle of the purple spot biodegradation process dynamics, to validate the proposed successional model and to deepen the understanding of the parchment degradation process. To this end, new data on the microbes responsible of the purple spots and their effects were gathered from three dramatically damaged parchments. These documents were chosen due to the deep and widespread purple spot damage, leading even to the loss of significant portion of the documents. In these extremely damaged conditions, we supposed that the pioneer species could have been more largely present at the beginning of the document history, so that they would have been more likely identified. Furthermore, as these documents belonged to the XVI-XVII century, *i*.*e*. they are centuries younger than the previously analysed one (XIII century), they also provided the opportunity to check if the different age/epoch could lead to different biodegradation outcomes. The three parchments are archived as *Faldone Patrizi A 19*, dated back XVI-XVII century A.D, and are still conserved in their original envelope, along with other documents. Their conservation state is truly poor (Fig. 1); surely the documents were exposed to high humidity while in the Archive of the Patrizi-Montoro family (in Rome). Since 1946, they are conserved in the Vatican Secret Archives, under controlled environmental conditions of relative humidity and temperature. The documents were analysed by the same multidisciplinary approach used in the previous study: NGS metagenomic analysis (Illumina platform), Raman spectroscopy and LTA analysis.

## Materials and Methods

### Metagenomic 16S analysis

#### Sample collection

Three documents belonging to a collection of dramatically damaged parchments of the Vatican Secret Archives were chosen for the bacterial and haloarchaeal community characterization. These documents were considered impossible to be restored and are archived as *Faldone Patrizi A 19*. The *Faldone* [binder] reports on the back the title ‘*Mandati*, *monitori e sentenze*. *Scritture*, *sommarii*, *ed altro relativo a cause tanto attive quanto passive*. *Dal 1359–1736’* [Mandates, monitors and judgments. Scriptures, summaries, and more about active or passive causes. From 1359 to 1736]. Small pieces (2–4 mm^2^) from three parchments were collected and identified as *A*, *B* and *C* samples: A document was entitled ‘*Octavianus Raggius Protonotarius Apostolicus*’ and dated 1640 A.D., B document was entitled ‘*Antonius…*’ dated about 1510, and C document was entitled ‘*Octavianus Raggius Protonotarius…*’ and dated 1639. The analysed samples were collected in sterile conditions from hardly purple stained or quite uncoloured areas in sterile conditions. From each document, three replicates were selectively collected from both purple and uncoloured less damaged areas (as control). Due to the dramatic and advanced state of biodeterioration of the documents, the three white pieces were chosen in the better preserved and less damaged area, as far as possible from the purple spots; in the most damaged parchment (C), the control samples were collected in an uncoloured area even if it was not clearly differentiated from the purple spotted one.

#### Bacterial pellet and DNA extraction

For the DNA metagenomic extraction, each parchment replicate was directly processed using the Power Soil®DNA isolation kit (Mo Bio, Carlsbad, CA, USA) following manufacturer’s guidance.

#### Illumina paired end sequencing

Purified DNA extracts were sent to the Molecular Research LP in TX, USA (MR DNA, http://www.mrdnalab.com/^6^) where PCR amplification of bacterial and haloarchaeal 16S rRNA gene and, successively, Illumina paired end sequencing were performed. The almost universal primers 515 F (Forward, 5-GTGCCAGCMGCCGCGGTAA-3) and 806 R (Reverse, 5-GGACTACHVGGGTWTCTAAT-3) which anneal to bacterial and archaeal 16S sequences, other than the SAR11 clade and some Crenarcheota and Taumarchaeota^7,8^, were used to amplify the phylogenetically highly variable regions V4^9,10^ of bacteria and haloarchaea. The 16S rRNA gene V4 variable region PCR primers 515/806 with barcode on the forward primer were used in a 30 cycle PCR (5 cycle used on PCR products) using the HotStarTaq Plus Master Mix Kit (Qiagen, USA) under the following conditions: 94 °C for 3 minutes, followed by 28 cycles of 94 °C for 30 seconds, 53 °C for 40 seconds and 72 °C for 1 minute, after which a final elongation step at 72 °C for 5 minutes was performed. After amplification, PCR products were checked in 2% agarose gel to determine the success of amplification and the relative intensity of bands. Then the purified PCR product was used to prepare DNA library by following Illumina TruSeq DNA library preparation protocol. Sequencing was performed at MR DNA (www.mrdnalab.com, Shallowater, TX, USA) on a MiSeq following the manufacturer’s guidelines.

#### Illumina paired-end sequencing data processing

Sequence data were processed using *Quantitative Insights Into Microbial Ecology* (QIIME) 1.1.9, an open-source bioinformatics pipeline used for the data analysis. The analysis steps for processing sequence data were developed based on state-of-the-art tools for metagenomic. In summary, full.fasta and full.qual files provided from MR DNA were converted in fastq files using the free software available on www.mrdnafreesoftware.com. After mapping file validation, reads were depleted of barcodes, filtered for quality (short sequences < 150 bp and ambiguous base calls sequences were deleted) and multiplexed to starting samples by nucleotide barcodes. The dataset was normalized to the sample with the lowest number of reads (sample AP1) at the common depth of 14,243 reads per sample, by random sub-sampling, in order to standardize samples differences. Operational Taxonomic Units (OTUs) were specified by clustering at 97% similarity (3% divergence). Two similarity methods implemented in QIIME, including BLAST and UCLUST, were used. A representative sequence from each OTU cluster was chosen and used for taxonomic identification and phylogenetic alignment. Prior to tree building, the alignment was filtered to remove positions, which are phylogenetically uninformative. Chimeras and singletons were identified and removed from the alignment, so that non-chimeric sequences were aligned and used to create a phylogenetic tree. Finally, OTUs were taxonomically classified using BLASTn against the databases derived from GreenGenes, RDPII and NCBI^10^, (http://rdp.cme.msu.edu, www.ncbi.nlm.nih.gov, respectively). Diversity metrics were calculated for each sample; the taxonomic and phylogenetic assignment was used to compare the types of community. To show the differences among the sample set, PCoA plots were generated. All the sequences processed in this study have been deposited in GenBank under the accession: SUB4105198 (MH490917:MH490934) and SUB4090063 (MH413071:MH4414454).

#### Prokaryotic community analyses

The free software QIIME was used to analyse the bacterial and haloarchaeal community structure and composition on the normalized OTU dataset: (i) rarefaction curves were created to assess sampling efficiency (Fig. S1, in Supporting Materials); (ii) Shannon index (H’) was performed to quantify diversity; (iii) Venn diagram was used to quantify the shared/unshared OTUs in the two set of samples; (iv) bar and pie charts were built to show the community composition and the relative abundance of taxa. Multivariate analyses on the OTU dataset were also performed, using the UniFrac unweighted metric within QIIME.

#### Statistical analyses

Principal Coordinates Analysis (PCoA) and ANOSIM, UniFrac unweighted and PERMANOVA were used in QIIME. PCoA ordination was performed on a Bray-Curtis distance matrix calculated between sampling plots with log (x + 1)-transformed OTUs abundance data. ANOSIM (n = 99 randomizations^11^) and PERMANOVA with Bonferroni correction (n = 999 permutations), were tested to find out significant differences in bacterial/haloarchaeal communities between purple damaged and uncoloured less damaged samples within each parchment and among the three parchments. The UniFrac unweighted analysis, which measures the difference between collections of sequences as the amount of evolutionary history that is unique to each one^12,13^, was used to evaluate if the microbial community structure and composition of the two types of samples differed significantly based on their identity and the abundances of the taxonomic groups present in the samples; statistical significance was defined at p < 0.05.

#### Growth of Halobacterium salinarum in the laboratory

The strain *Halobacterium salinarum* DSM 3754 was grown on home-made medium (DSMZ medium #97): casamino acids 7.5 g, yeast extract 10 g, Na_3_-citrate 3 g, KCl 2 g, MgSO_4_ 7H_2_O 20 g, FeSO_4_ 7H_2_O 0.05 g, MnSO_4_ H_2_O 0.2 mg, NaCl 250 g, agar 20 g, in 1 l of deionized water, adjusted pH to 7.4. The cultures were incubated at 37 °C in humidified chamber for 21 days.

### Chemical analyses

#### Raman analysis

Raman analyses were performed on purple damaged samples^1^ by chemically extracting pigments from purple spots, and on *Halobacterium* harvested colonies; reddish colonies were analysed directly without any pre-treatment. Due to heterogeneity of the parchment supports and the high entity of damage, less damaged areas were difficult to be focalized, providing fluorescence as the dominant response.

Raman analyses were performed by using a micro-Raman spectrometer eXplora system (Horiba) with a laser source at 785 nm for parchment samples and 532 nm for the grown colonies.

### Physical analyses

#### Light Transmission Analysis (LTA) with visual control by microscopy

In order to assess the deterioration state of the collagen both in purple and uncoloured less damaged areas, the induced hydrothermal denaturation of the collagen has been analysed by means of the LTA technique, integrated by the microscopy continuous monitoring, as in Migliore *et al*.^1^. In this study, the signal amplitude was recorded for the whole process of hydrothermal denaturation, every 0.08 °C during the heating scans, running from 30 to 85 °C.

## Results

### Metagenomic 16S analysis

QIIME bioinformatic analysis revealed a total of 830,435 sequences belonging to Bacteria and Haloarchaea. As a whole, purple samples coming from the three documents yielded 474,973 sequences (nine replicates, sample yield ranging from 14,243 to 108,388), while uncoloured samples coming from the three documents yielded 355,462 sequences (nine replicates, sample yield ranging from 18,376 to 97,390) (Table 1). The sequences were assigned to a total 1,402 OTUs (1251 from the purple samples and 1240 from the unstained ones), out of which 1089 were common to the two batches.

**Table 1:** Purple damaged (P) and uncoloured less damaged (U) samples yields, from each replicate (#1, 2, 3) of the three parchments (A, B, C). Each sequence was assigned to the appropriate Operational Taxonomic Unit (OTU) and the total number of OTUs per batch is reported.

P		U	
Sample	N. Sequences	Sample	N. Sequences
**AP1**	14243	**AU1**	18376
**AP2**	20537	**AU2**	19421
**AP3**	23087	**AU3**	19927
**BP1**	33542	**BU1**	23409
**BP2**	53584	**BU2**	24154
**BP3**	62906	**BU3**	33498
**CP1**	74662	**CU1**	48868
**CP2**	84024	**CU2**	70419
**CP3**	108388	**CU3**	97390
**Total**	474973	**Total**	355462
**N. OTUs**	1251	**N. OTUs**	1240

**Figure 2:**
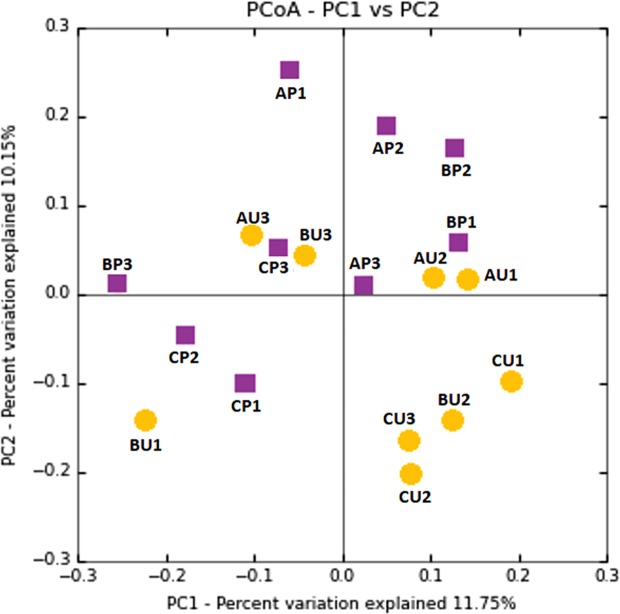
PCoA based on Bray-Curtis distances of bacterial/haloarchaeal composition between purple-damaged (square purple spots) and less damaged areas (round warm yellow spots) of parchments. No clear patterns of separation are shown. Significance (ANOSIM, p > 0,05) was calculated by permutation test with pseudo F-ratio. Each sample is identified as: parchment (A, B, C), purple damaged (P) or uncoloured less damaged samples (U) and replicate (#1, 2, 3).

By random subsampling, the entire dataset was normalized to the lowest common depth of 14,243 (sample AP1) sequence reads per sample, in order to standardize differences among samples. Taking into consideration the amount of available material (2–4 mm^2^ of parchment), rarefaction curves confirmed the efficiency of the sampling (Fig. S1, Supporting Materials). Shannon diversity index (H′), calculated on the entire dataset, was 2.60 ± 0.01 for the purple damaged samples, and 3.45 ± 0.01 in the uncoloured less damaged ones; in fact, it was lower in each of the purple damaged samples if compared to each of the uncoloured less damaged ones, being 2.70 ± 0.78 in the sample AP, 2.47 ± 0.03 in the sample BP, 1.90 ± 0.40 in the sample CP *vs* 3.02 ± 0.99 in the sample AU, 3.03 ± 0.60 in the sample BU, 3.62 ± 0.10 in the sample CU. PCoA plot (Fig. 2) was used to compare the composition of purple damaged and uncoloured less damaged samples. It showed that the microbial composition of the two sets of samples overlap. ANOSIM analysis revealed that differences both between the purple damaged and uncoloured less damaged samples in each parchment and the pooled dataset of purple damaged or less damaged samples were not statistically significant (99 permutations, p > 0.05).

**Figure 3:**
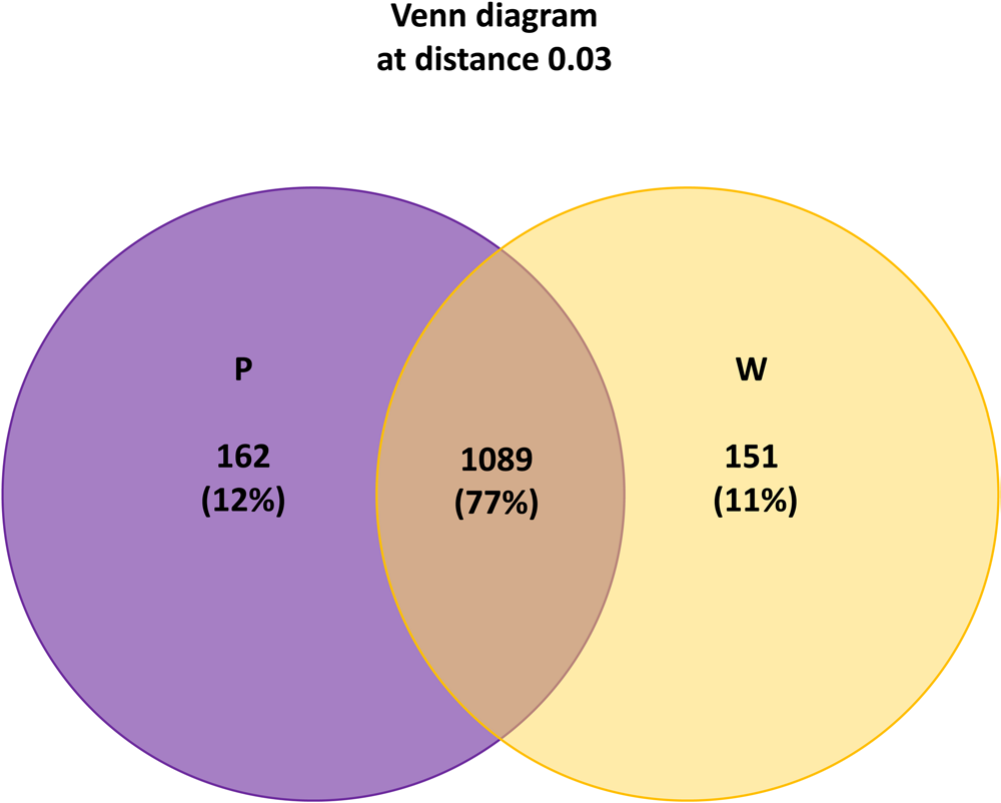
Unique or shared OTUs in purple damaged (P) and uncoloured less damaged samples (U) of the three parchments. The number and percent of OTUs are also reported. The total number of OTUs is 1,402.

The Venn diagram (Fig. 3) showed how OTUs are differently distributed between the two sets of samples: 162 OTUs (12%) were only found in purple damaged samples (183,555 sequences, 34% of the sequences); 151 OTUs (11%) were only found in uncoloured less damaged samples (64,044 sequences, 12%); while the great majority, 1,089 OTUs (77%) were shared (829,258 sequences, 99%).

A detailed list of the total OTUs collected analysing both purple damaged and uncoloured less damaged samples of the three parchments is reported in Table S1 (OTU Table, Supporting Materials) along with the UPGMA tree (Fig. S2, Supporting Materials).

**Figure 4:**
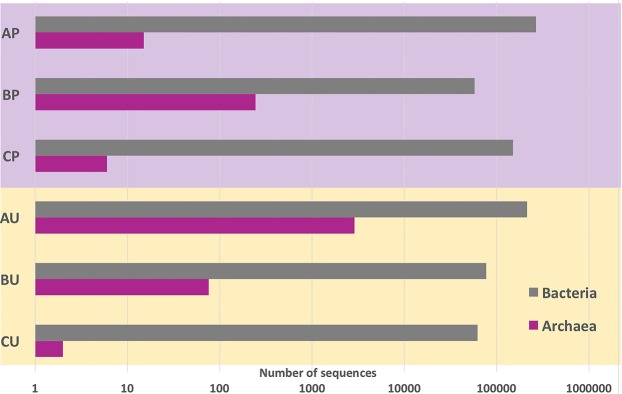
Haloarchaeal and bacterial sequence distribution, in purple damaged (P, in the purple box) and uncoloured less damaged (U, in the warm yellow box) samples in the three parchment samples (**A–C** documents).

Both Halobacteria and Bacteria were found. Halophylic Archaea accounted for 3,221 sequences assigned to one single OTU and found in both set of samples; Bacteria accounted for 827,214 sequences, assigned to 1,401 OTUs. Figure 4 shows how haloarchaeal and bacterial sequences are distributed in purple damaged and uncoloured less damaged samples. The detailed distribution of the sequences found in each replicate sample from the three parchments is reported in Table S2, Supporting Materials.

**Figure 5:**
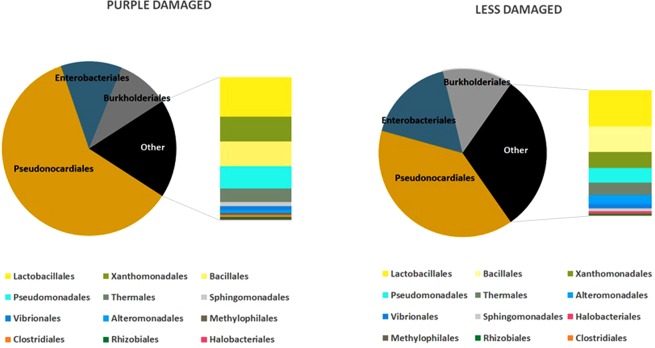
Distribution of taxa at order level in the purple damaged (left) and uncoloured less damaged (right) samples of the three parchments, as percentage of sequences found in the samples from the Faldone Patrizi A 19.

The haloarchaeal OTU belonged to the genus *Halobacterium*, with a 100% identity with several *H*. *salinarum* sequences, including the type strain one (96-R6^T^, NR_025555.1). They are more frequent in the purple damaged samples than in the less damaged uncoloured ones, other than in the document A. The main bacterial phyla were Actinobacteria, Proteobacteria and Firmicutes. All of these microorganisms are observed in both the sample sets, reported in Fig. 4 as the sum of all bacterial strains found in the three replicates of each document, and in Fig. 5 as the distribution of the main taxa in the two batches (purple damaged or uncoloured less damaged samples) obtained from the three parchments.

The distribution of taxa, at order level (Fig. 5), shows that the dominant groups in both sets of data are the environmental, human/animal associated bacteria, Pseudonocardiales, Enterobacteriales and Burkholderiales; these groups are present at different percentage in the two batches. On the contrary, the early colonizers - marine bacteria, as the Vibrionales - are found at very low percentage.

Among Bacteria, the class Actinobacteria was prevalent in both purple damaged [#P 61%; ranging from 69% (CP) to 55% (BP)] and uncoloured less damaged samples [#U 44.9%; ranging from 59.7% (BU) to 8% (CU)], with Pseudonocardiales being the prevalent order (#P 98.6%; #U 92.3%).

The second most represented class is Proteobacteria; which showed a slight prevalence in the less damaged batch [#P 28.4%, ranging from 30.6% (AP) to 22.4% (CP); #U 38.8%, ranging from 60.5% (CU) to 31.5% (BU)]. The decreasing frequency order in the Class was: Gamma-Proteobacteria [63.4%; #P 63% and #U 63.8%], Beta-Proteobacteria [33%; #P 32.8% and #U 33.3%] and Alpha-Proteobacteria [3.5%; #P 4.2% and #U 2.9%]. The Delta- and Epsilon- subclasses were practically absent. Enterobacteriales, Xhantomonadales, Pseudomonadales (Gamma-Proteobacteria); Burkholderiales (Beta-Proteobacteria) and Sphingomonadales (Alpha-Proteobacteria) were the more frequently encountered orders.

Firmicutes [#P 8.2%, ranging from 9.7% (BP) to 8.3% (AP); #U 12.8%, ranging from 27.5% (CU) to 7.9% (BU)] were mainly Bacillales and Lactobacillales.

Rare taxa, in both purple damaged and uncoloured less damaged sample areas were: *Alteromonadales* among Gamma-Proteobacteria, *Methylophilales* among Beta-Proteobacteria, *Thermales* among Deinococci, *Rhizobiales* and *Sphingomonadales* among Alpha-Proteobacteria (see OTU Table, Table S1 in Supporting Materials).

UniFrac unweighted and PERMANOVA (with Bonferroni correction, 999 permutations) demonstrated that the differences between damaged and less damaged areas within each parchment were not significant (PERMANOVA: AP *vs* AU, p = 0.1; BP *vs* BU, p = 0.2; CP *vs* CU, p = 0.1; UniFrac unweighted: AP *vs* AU, BP *vs* BU, CP *vs* CU, all comparisons p > 0.5). However, when the complete dataset from the three parchments is tested, a statistical difference between the purple and uncoloured samples is found (PERMANOVA: p = 0.04; UniFrac unweighted: p < 0.05).

### Chemical analysis

**Figure 6:**
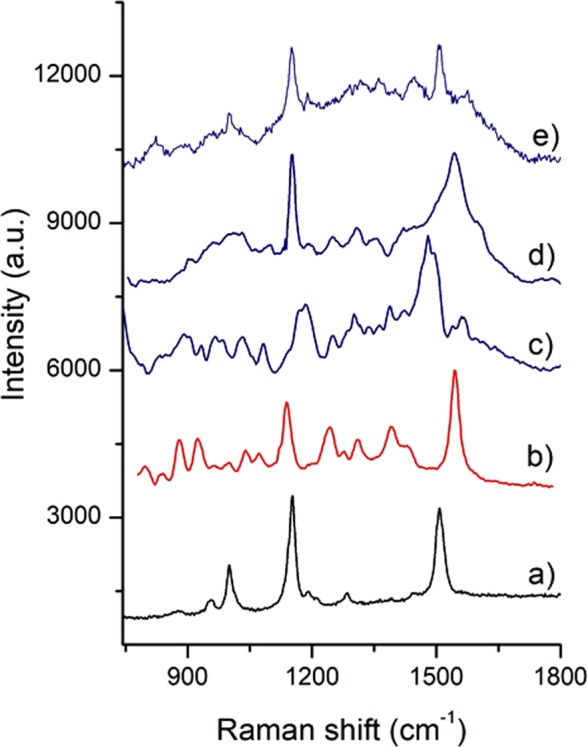
Raman spectra. (**a**) Halobacterium salinarum colonies directly taken from the growth medium; (**b**) pigment extracted from the parchment A.A. Arm. I-XVIII 3328, XIII century^1^; (**c–e**) extracted pigment from the A, B and C parchments of the Faldone Patrizi A 19, XVI-XVII century.

Raman spectra acquired from *Halobacterium salinarum* (Fig. 6a) contained major features at 1001, 1152, and 1508 cm^−1^. These bands are attributed to the bacterioruberin, the main carotenoid component responsible for the colour of the red archaea; the signal results very intense because of the resonance condition due to the use of green laser source^14^. Other less intense signals are found and could be attribute to bacteriorhodopsin contribution to the spectrum.

Raman spectra of extracted pigments from parchments are reported in Fig. 6(b–e). In particular Fig. 6(b) is one of the Raman spectra already reported in our previous work^1^, while (c,d) and (e) came from the A, B, C samples. All the spectra are more complex than that acquired from the cultured cells due to the nature of the samples; moreover, no resonance is expected using the 785 nm laser source. The signals of bacterioruberin are almost present in every samples. Furthermore, the red shift detected in particular from the band at 1508 cm^−1^, in the spectra c), d) and e) together with the other signatures of the spectra could be attributed to retinal isomers that are the polyene chromophore bound to opsins proteins. It is important to underline that bacteriorhodopsin, rhodopsin-like pigments, and retinal isomers exhibit almost overlapping Raman spectra. Their dominant feature in the Raman spectra is a complex strong band in the 1575 cm^−1^ region; moreover, other intense signals are sited at about 870, 930, 1260, 1320 and 1450 cm^−1^ ^15,16^.

### LTA analysis

#### Denaturation analysis

**Figure 7:**
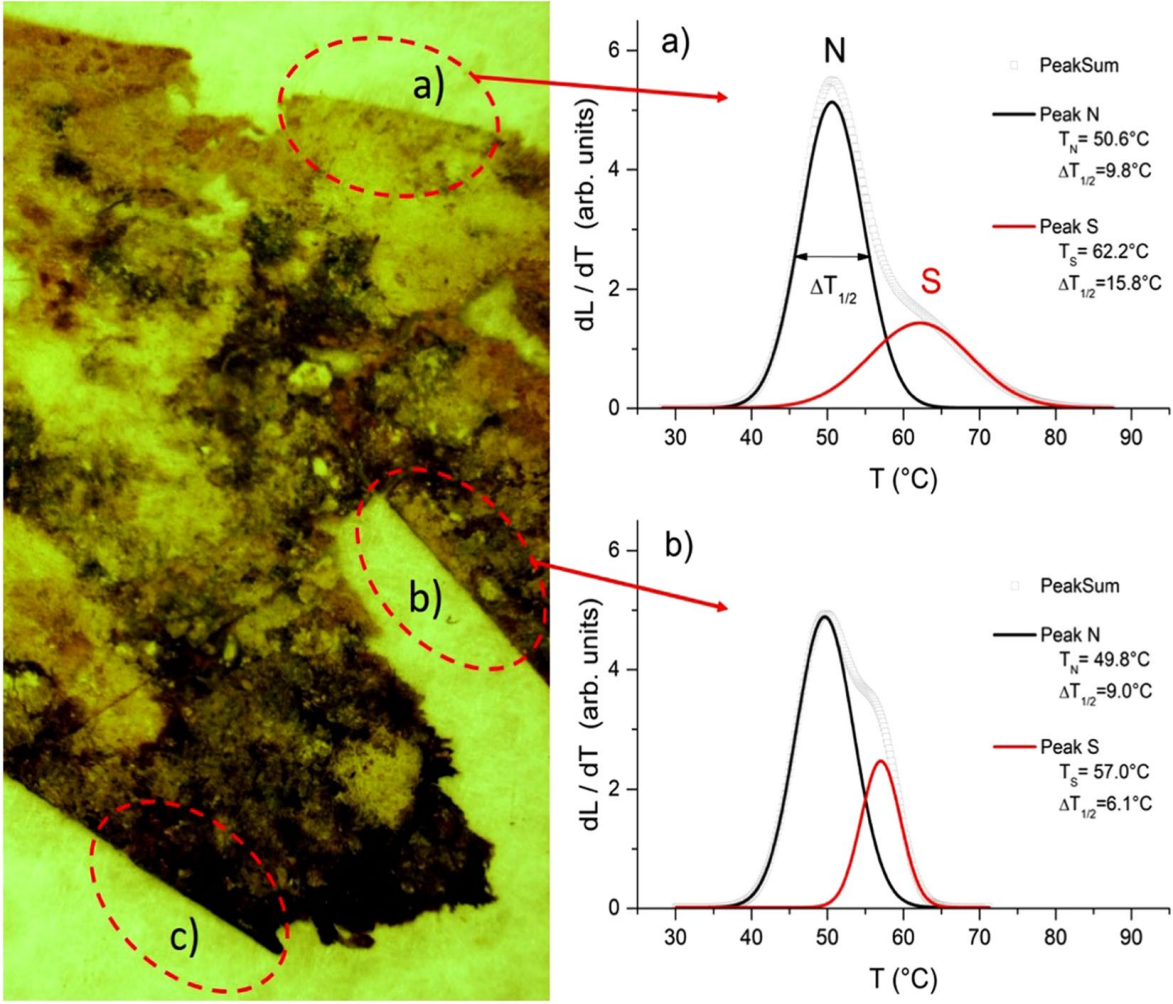
Parchment structural alterations identified by Light Transmission Analysis (LTA). On the left, a piece of parchment from Faldone Patrizi A 19, (in red light, that shows as dark brown the purple stained areas); on the right, the corresponding denaturation curves: (**a**) uncoloured less damaged and (**b**) purple damaged sample, the samples being representative of all the three documents. The image is representative of the parchment patch from where samples have been withdrawn (circled areas). The grey curves represent the best fit of the thermal denaturation rate dL/dT plotted as a function of temperature T, being L the measured amplitude of the signal generated by the transmitted light. They have been deconvolved in two curves (black and red lines) representing the contribution to the denaturation by collagen fractions with different thermal stability. The (**c**) samples did not give repeatable results and are not shown.

Figure 7 shows two typical LTA plots obtained from the uncoloured (a, less damaged) and purple (b, more damaged) parchment samples withdrawn from the areas circled in the aside image. Samples from the highly damaged areas, c), were also analysed but the results were neither reproducible, nor univocal and are not shown here because of their unsoundness. The grey curves represent the variation rate of the signal generated by the unscattered transmitted light, as a function of temperature and express the intensity of the hydrothermal denaturation process. The profile of these curves can be seen as the combination of two peak curves associated with the contributions to the denaturation of material fractions, characterized by collagen with different chemical stability. In particular, the low temperature peak describes the denaturation of the less stable collagen, classified as *native* (N) in previous works^1,17^, while the high temperature peak is related to the denaturation of a more stable collagen, the so called *stabilized* (S) one^17,18^, typical of the fibres sheath and characterized by a larger thermal stability. It has been shown^17,18^ that the deterioration of the collagen populations leads to a temperature downshift of the related LTA peak^1^. In this view the graph of Fig. 7(b) shows that both S and N peaks of the purple parchment have a lower characteristic temperature with respect to the corresponding one of Fig. 7(a), revealing a larger damage, possibly associated with the bacterial action in the purple spots area. Nevertheless, the temperature difference ΔTn = −0.8 °C between the N peaks position is very small and can be considered of no relevance. A quite different situation is observed for the S peaks. The temperature change is large (Ts = −5.2 °C), revealing a relevant amount of damage produced in the collagen S, which appears narrower in the purple sample. Furthermore, the thermal stability of the S population for the uncoloured less damaged samples (Ts = 62.2 °C) is much lower than in the uncoloured undamaged samples investigated in Migliore *et al*. ^1^; (Ts = 70.4 °C).

## Discussion

In this study, the purple spot damage of ancient parchments was studied by an interdisciplinary approach, comparing hardly purple *vs* uncoloured (less damaged) areas from three dramatically damaged parchments archived as *Faldone Patrizi A 19* in the Vatican Secret Archives, dated back to XVI-XVII century A.D. In a previous work on the parchment roll *A*.*A*. *Arm*. *I-XVIII 3328*, studied by the same interdisciplinary approach^1^, the Raman analysis detected the trace of the past presence of Haloarchaea, even in the absence of any amplifiable DNA. Haloarchaea are known to cause the red-heats affecting the modern brine cured hides and reach up to 10^8^ CFU/mL in the brines, according to the used salt and the brining conditions^19,20^. So, we chose extremely damaged documents, on the premise that a possible larger amount of halobacteria in the original brines could have caused a faster and widespread formation of the purple spots, and that the DNA of the putative pioneer species would have been more likely found out. Actually, in these largely damaged documents, the NGS revealed the presence of *Halobacterium salinarum*, and the Raman spectroscopy confirmed the presence of bacteriorhodopsin together with bacterioruberin in all the purple samples^1^. Bacterioruberin is also produced by some species of Actinobacteria out of Micrococcales and Corynebacteriales order^21,22^ which have been found also in the documents from *Faldone Patrizi A 19*, at low percentage in all samples. Nevertheless, the possibility of them to determine the purple spot-damage had been already ruled out in the previous work, as their number was comparable in the neatly undamaged and damaged samples from *A*.*A*. *Arm*. *I-XVIII 3328*^1^. All the Raman spectra show two main bands in the 1152, and 1508 cm^−1^ regions and are superimposable to the one from the laboratory cultured *H*. *salinarum*, which, in turn, is perfectly superimposed to the one from the *A*.*A*. *Arm*. *I-XVIII 3328*. As suggested by Marshall *et al*.^23^, bacteriorhodopsin and bacterioruberin can be used as a halophilic archaeal biomarkers using resonance Raman spectroscopy. In our experiment only the spectra of colonies were taken in resonance conditions (using 532 nm laser source) and, as expected, are dominated by the bacterioruberin signals.

The spectra taken in non-resonance conditions (785 nm laser source) from the A, B, C documents highlight the effects of the biological degradation on the pigments, at a such high rate that even the spectra of the chemically stable chromophore from bacteriorhodopsin (retinal isomers), although well recognizable, are smoothed and modified. By comparing the *A*.*A*. *Arm*. *I-XVIII 3328* document dated 1244 A.D.^1^, with the more recent A, B, C documents, dated from 1510 to 1640 A.D., is quite clear that the different degradation degree is more probably due to the extent of the biological attack than to an age effect.

Regarding the prokaryotic biodeteriogens, in these three documents from the *Faldone Patrizi A 19* NGS found the traces of halobacteria, with 3221 sequences, at different percentages in all samples. Other than haloarchaea, the bacterial taxa in the three documents are almost the same as those found in the *A*.*A*. *Arm*. *I-XVIII 3328* parchment roll from the Fondo *‘Archivum Arcis’*. The roll, however, is still quite well preserved, and the quantitative distribution of taxa in the purple and undamaged areas was dramatically different.

On the contrary, in the *Faldone Patrizi A 19* documents, the comparison of the bacterial communities did not show significant differences within each parchment, as the majority of OTUs was shared between the two datasets, although the H’ diversity is lower in the purple damaged samples. Furthermore, the incidence of unique OTUs, as a total number of sequences, is much higher in purple damaged samples if compared to the uncoloured less damaged ones (three times higher in purple damaged samples, = 34% of the 183,555 sequences). This, highlights a significantly different colonization rate between the two set of samples, while analyzed as the complete dataset. The exiguity of sample material and the variability of microbial community composition, even at this small scale, are responsible of the absence of significant differences within each document. Nevertheless, while comparing the entire dataset, the algorithm is able to highlight the differences. This implies that the environmental and nutritional conditions in the damaged parchment environment select relatively few microbial strains and that, even in the same document, the damaged and undamaged areas offer different environments. However, in all samples from the *Faldone Patrizi A 19* the dominant component of the colonizing microbial community belonged to Proteobacteria, Firmicutes and Actinobacteria.

**Figure 8:**
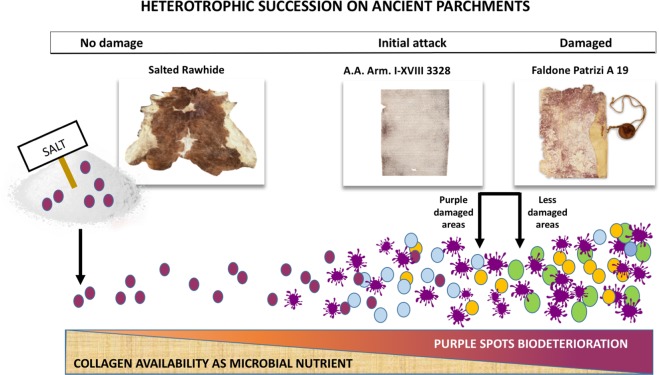
Dynamics of the microbial succession in the parchment. In the deterioration process the main actors are in the sequence: haloarchea (purple), halotolerant bacteria (light blue), actinobacteria (gold) and fungi (green).

In our previous study we hypothesized a two phases heterotrophic microbial succession, able to degrade the parchment. The first-phase ‘pioneer’ colonizers of this succession process would have been halophylic and halotolerant microorganisms, while the second-phase ‘late’ colonizers were recruited among the environmentally available ones. This work allowed to have a more accurate picture of the parchment succession (Fig. 8).

The presence of *Halobacterium salinarum* in the *Faldone Patrizi A 19* parchments put in place a first (pivotal) piece in our biodegradation jigsaw puzzle, confirming the identity of the actual pioneer population. The brining of the hides in ancient times, as nowadays, was a common step of the manufacturing process of both parchments and leathers. In the brine, the halophilic and halotolerant microbes from the marine salt, can grow and enter into the hides, forming the core of the purple spot damage as it happens in brine-cured leathers^24^. They grow inside hides by producing proteolytic and lipolytic enzymes which attack and degrade the parchment collagen matrix^20^.

Later, maybe when the salt concentration within the parchment lowered, halobacteria lysed, in the hot-spots where they had grown up, releasing bacteriorhodopsin and cellular content and providing a nutrient and energy supplement. Such a boost allowed other halotolerant early colonizers, such as the proteobacterial taxa found in the parchments (mainly Gammaproteobacteria) and Firmicutes to rapidly utilize the nutrients made available by the *Halobacterium*’s primal colonization, growing and wiping away the halobacterial debris, except for the persistent bacteriorhodopsin purple derived compounds. So, the absence^1^ or the low percentage of the Haloarcheal OTUs are explained by the intrinsic mechanism of the ecological succession. Besides the bacterial attack, DNase mediated ‘self-degradation’^25^, and/or the possible activation of temperate phages^26^ could have helped to cancel the DNA haloarcheal traces. Already in the first steps of the parchment manifacture, the history of each document/hide can have been biased by several factors, influencing the amount of haloarchaea, such as the time of brining that was sometimes prolonged to several days^27^, the organic content in the brine, related to both the number of treated hides and the quality/provenience of the salt^19,20^.

The first phase of the succession, therefore, is predictable as mainly driven by the brining process - according to the Clements model^28^. Differently, the second phase attack to the parchment, is driven by a casual (Gleasonian) model^29^, and puts in place a second piece of the puzzle.

Even the second-phase bacteria, depend on the history of each parchment, that is, on the environment where the parchment was kept (libraries, bookcases, scholars, etc.) and are selected by the features of the finished parchment. In this study, as in the previous one, Pseudonocardiaceae (soil-dwelling, ubiquitous, often able to stand harsh environments) have been detected as the major colonizers of all the samples. Due to their wide metabolic versatility, Actinobacteria can be very efficient in a less rich environment on the long-time of parchment colonization/biodegradation, and are able to outcompete the previous colonizers. In the late colonization phases even some human-derived species, like staphylococci and enterobacteria can be found.

Therefore, it is not surprising that Actinobacteria (mainly Pseudonocardiales) have been frequently pointed out as the responsible of the parchment deterioration as, at the end point, they are actually dominant on the damaged parchments, even in the undamaged portions of the documents [1–2,30 and this study]. Concurrently the molecular approaches such as 16S libraries, and even the much more sensitive NGS, take a snapshot of the archaeal and bacterial colonizers which arrived on the parchments over the centuries and, obviously, the more recent are better represented than the old ones. This explains why the composition of the prokaryotic community found in the *Faldone Patrizi A 19* documents accounts for a prevalence of the second phase taxa, as even the halotolerant Gamma-Proteobacteria are quite few and the Actinobacteria definitely dominate the scene. In the *Faldone Patrizi A 19* documents they had a long time to degrade the parchment, achieving an almost complete ‘recycling/mineralization’ of all the nutrients in some part of them, as several portions of the document are deeply destroyed or even lost (Fig. 1). Most probably they can also benefit of the initial lesions produced by the first colonizers to penetrate the document, adding more and more collagen degradation in the already damaged stained spots.

Moreover, Actinobacteria are surely involved in the damage, so as fungi^30,31^, due to their ability to deeply attack the collagen, but they participate to the last act of the parchment tragedy, rather than to its causal trigger, at the first beginning.

**Figure 9:**
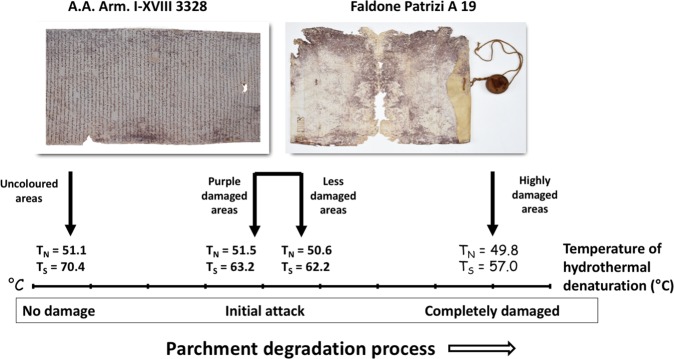
Steps of the parchment degradation process. They were identified by the temperatures of hydrothermal denaturation measured in both the A.A. Arm. I-XVIII 3328 parchment roll (left)^1^ and in one of the parchments analysed in this study (right; parchment B).

As a final point, by comparing the results on the *A*.*A*. *Arm*. *I-XVIII 3328* roll and the *Faldone Patrizi A 19* parchments, the temporal dimension and the efficiency of the microbial succession is clearly quantified by LTA (Fig. 9). LTA gives insight on the collagen degradation: the peak temperatures for N and S depicts the thermal stability of the collagen populations, the higher the peak temperature, the higher the stability. In the *Faldone Patrizi A 19* samples, a much higher degradation of the so-called *stabilized* collagen population (S; Ts = − 5.2 °C) can be found as compared to the *native* (N) one: in these samples the S collagen population is dramatically damaged, while the N one seems almost unchanged.

Downshift of the denaturation temperatures have been already reported in Migliore *et al*.^1^ for the *A*.*A*. *Arm*. *I-XVIII 3328* parchment roll, in which a large downshift of T_S_ (−7.2 °C) and a small variation of T_N_ (+1.5 °C) were observed. In this document, however, the width of the T_S_ peak increased with the deterioration, revealing a process that made the stabilized collagen population less homogeneous. Differently, in the measurements on the *Faldone Patrizi A 19* documents (Fig. 7), the T_S_ peak appears narrower in the purple sample. Furthermore, it is worth noting that the thermal stability of the S population for the undamaged samples of the *A*.*A*. *Arm*. *I-XVIII 3328* parchment roll was much higher than the one of the *Faldone Patrizi A 19* documents, and maybe different starting thermal stability can address different dynamics of the deterioration process.

The results on the three *Faldone Patrizi A 19* documents seem to constitute a *continuum* with those found on the *A*.*A*. *Arm*. *I-XVIII 3328* parchment roll (Fig. 9). In the framework of the progressive process of collagen degradation and utilization, intrinsic in the successional process, the samples from the *A*.*A*. *Arm*. *I-XVIII 3328* document represent the starting point, a well-preserved structure of the parchment (the uncoloured samples), and a first degradation step (purple samples). At the starting point, a diffused matrix encompassing the more robust fibres was found, while, as the process of collagen degradation goes on, the matrix is completely absent and only the more robust fibres can be found. The *Faldone Patrizi A 19* documents seems to show how the process of collagen degradation proceeds/continues, due to the second-phase bacterial colonizers. Actinobacteria are able to deeply penetrate into the collagen structure and damage even the more robust fibres, by a slow but constant action.

The uncoloured samples show comparable collagen stability to the *A*.*A*. *Arm*. *I-XVIII 3328* damaged samples, as the temperatures of hydrothermal denaturation shown by the curves in Fig. 7(a), are T_N_ = 50.6 *vs* 51.5 °C and T_S_ = 62.2 *vs* 63.2 °C, respectively. Hence, it is possible to hypothesize that a general collagen degradation already happened in the entire document, even in the uncoloured areas. The purple damaged samples in some cases were so deteriorated (see, for instance, the circled area c in Fig. 7) that was impossible to get reproducible, univocal hydrothermal denaturation data. Other less stained samples, surprisingly, displayed a relevant quantitative and qualitative amount of damage in the S collagen, while the N population seemed almost unchanged. This result could be explained in term of a biological attack to the stabilizing crosslinks in the S collagen present in the more robust fibers, representing the frame structures of the parchment^17,18^; probably microbes are able to attack and degrade collagen to a less structured status. This degraded collagen has hydrothermal denaturation temperatures similar to the N population and it could accumulate and mimic the less structured collagen populations, which are easily wiped away from the bacterial/haloarchaeal colonizers, as observed in the *A*.*A*. *Arm*. *I-XVIII 3328* parchment. The loss of these ‘bearing structures’ is responsible for the cracking, detachment and loss of superficial pieces at the flesh side of the parchment, including the written areas.

In conclusion, the integrated advanced multidisciplinary approach adopted in this study, was able to validate the successional model hypothesized in the previous study, and to further shed light on the causes and processes involved in historical parchment deterioration, successfully deciphering other pieces of the purple spot degradation jigsaw puzzle. The importance of this study is not only limited to the ancient parchment story but also to the nowadays problem of the red-heat deterioration in brine cured hides, that show similar features with purple spots, although a common causative agent has not been demonstrated so far. Hence, this study opens opportunities of intervention on ancient parchments, but may significantly contribute to the efficiency of leather manufacturing.

## Supplementary information

Figg. S1 & S2

OTUs identification

Number of sequences found in each replicate sample from the three parchments
