# Supplementary material for: Three ancient documents solve the jigsaw of the parchment purple spot deterioration and validate the microbial succession model
Source: Sci Rep. 2019 Feb 7;9:1623. doi: 10.1038/s41598-018-37651-y (PMC6367363; doi:10.1038/s41598-018-37651-y)

Three ancient documents solve the jigsaw of the parchment purple spot deterioration and validate the microbial succession model

Migliore L., PeriniN., Mercuri F., Orlanducci S., Rubechini A. & Thaller M.C.

Fig. S1. Rarefaction curves built on each replicate sample (#1, 2, 3) from both purple damaged samples (P) and uncoloured less damaged samples (U) of the three parchments

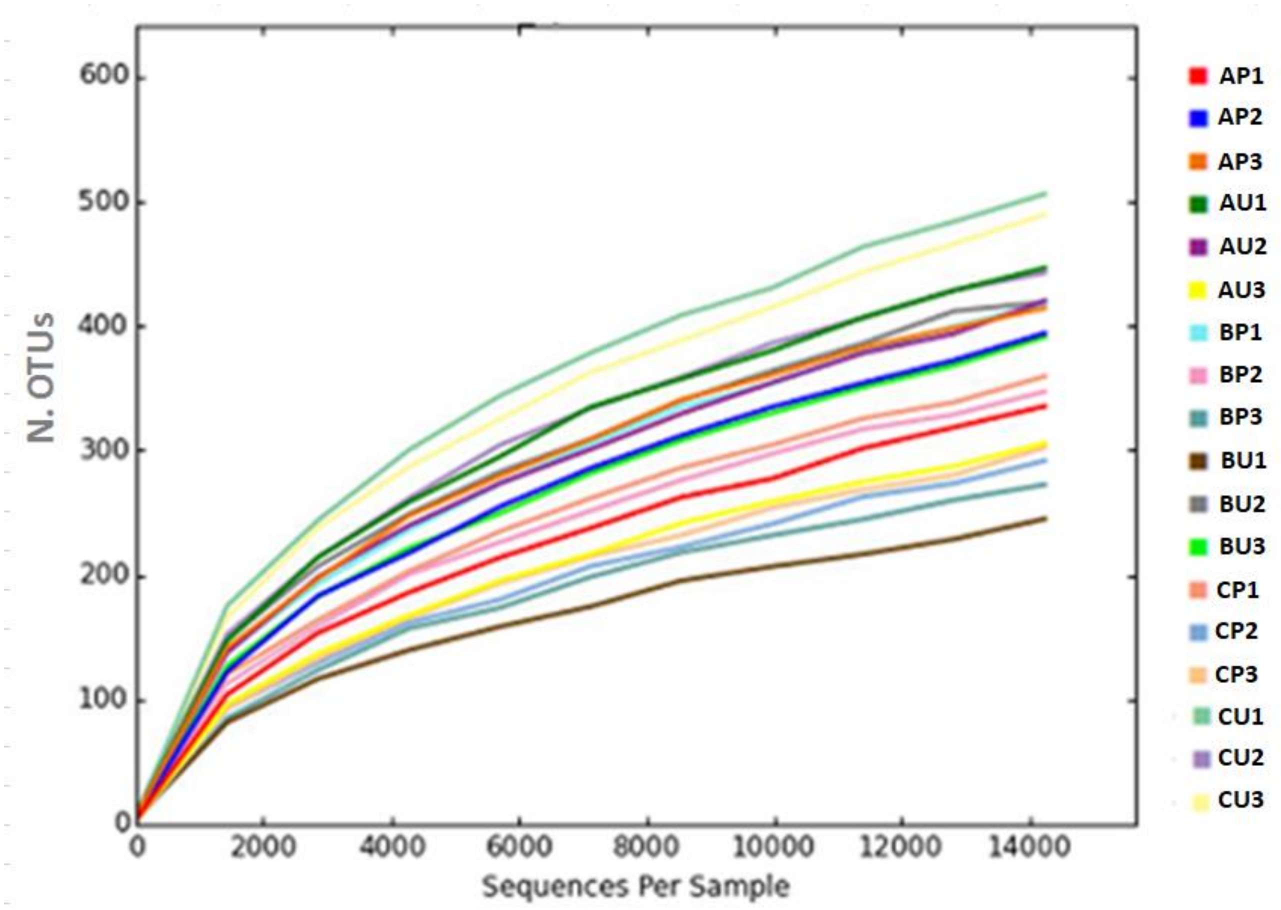

Three ancient documents solve the jigsaw of the parchment purple spot deterioration and validate the microbial succession model

Migliore L., Perini N., Mercuri F., Orlanducci S., Rubechini A. & Thaller M.C.

Fig. S2. UPGMA clustering built on each replicate sample (#1, 2, 3) from both purple damaged samples (P) and uncoloured less damaged samples (U) of the three parchments (A, B, C)

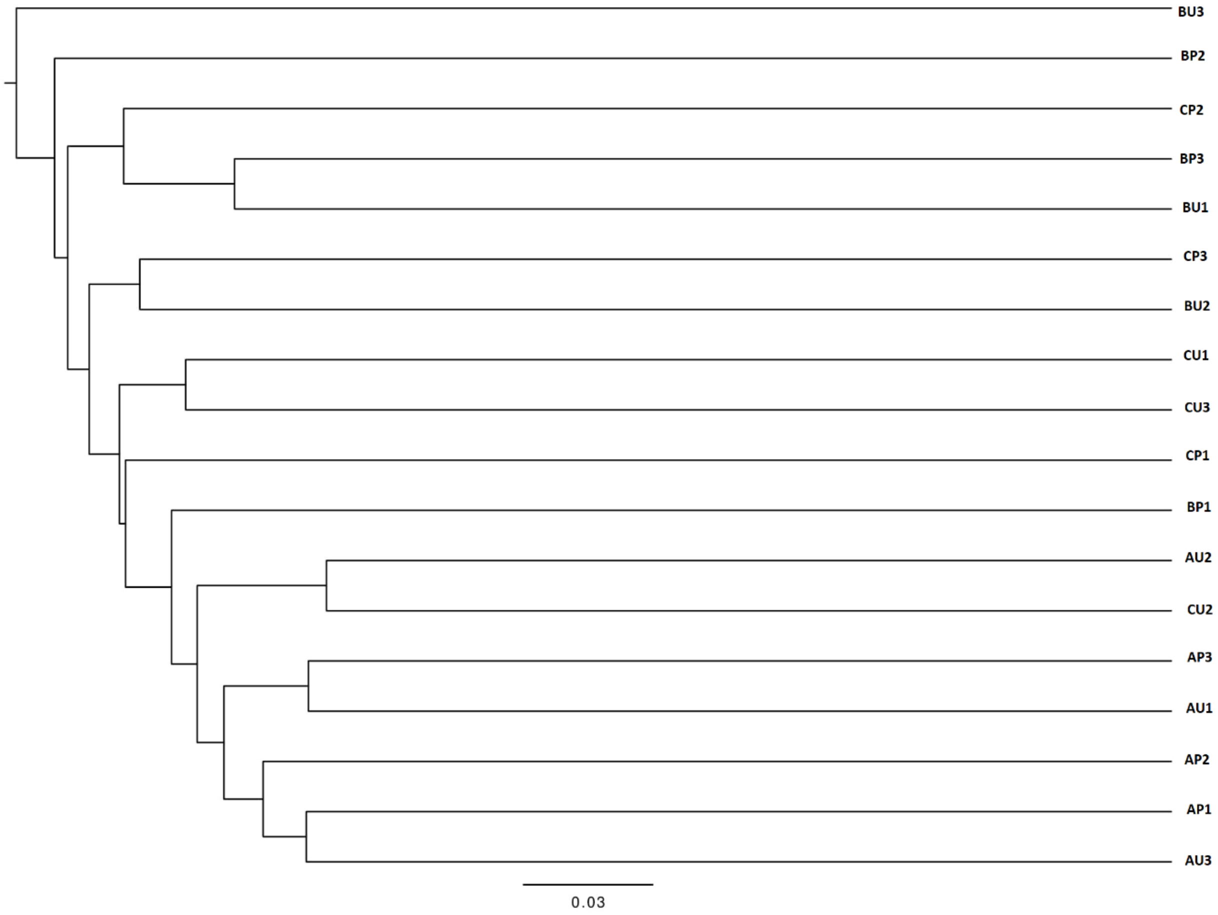

Supplement: Supplementary file 1 — Figg. S1 & S2 [file 41598_2018_37651_MOESM1_ESM.pdf]
