# Supplementary material for: Three ancient documents solve the jigsaw of the parchment purple spot deterioration and validate the microbial succession model
Source: Sci Rep. 2019 Feb 7;9:1623. doi: 10.1038/s41598-018-37651-y (PMC6367363; doi:10.1038/s41598-018-37651-y)
Supplement: Supplementary file 2 — OTUs identification [file 41598_2018_37651_MOESM2_ESM.pdf]

Three ancient documents solve the jigsaw of the parchment purple spot deterioration and validate the microbial succession model.

Migliore L., Perini N., Mercuri F., Orlanducci S. Rubechini A. & Thaller M.C.

Table S1. OTUs identification

#OTU	DOMAIN (%)	PHYLUM (%)	CLASS/SUBCLASS (%)	ORDER (%)	FAMILY (%)	GENUS (%)
OTU0001	Bacteria (100)	Proteobacteria (100)	Alphaproteobacteria (100)	Rhizobiales (99)	Unclassified (<80)	Unclassified (<80)
OTU0002	Bacteria (100)	Proteobacteria (100)	Alphaproteobacteria (100)	Rhodobacterales (100)	Rhodobacteraceae (100)	*Pseudorhodobacte*[illegible] *(94)*
OTU0003	Bacteria (100)	Proteobacteria (100)	Betaproteobacteria (100)	Rhodocyclales (98)	Rhodocyclaceae (98)	Unclassified (<80)
OTU0004	Bacteria (100)	Proteobacteria (100)	Betaproteobacteria (100)	Burkholderiales (100)	Burkholderiaceae (100)	*Pandoraea (98)*
OTU0005	Bacteria (100)	Proteobacteria (100)	Gammaproteobacteria (100)	Xanthomonadales (100)	Xanthomonadaceae (100)	*Stenotrophomonas (100)*
OTU0006	Bacteria (100)	Proteobacteria (100)	Gammaproteobacteria (100)	Xanthomonadales (100)	Xanthomonadaceae (100)	*Stenotrophomonas (99)*
OTU0007	Bacteria (100)	Proteobacteria (100)	Gammaproteobacteria (100)	Pseudomonadales (98)	Pseudomonadaceae (98)	Unclassified (<80)
OTU0008	Bacteria (100)	Proteobacteria (100)	Gammaproteobacteria (100)	Pseudomonadales (100)	Pseudomonadaceae (100)	*Pseudomonas (95)*
OTU0009	Bacteria (100)	Proteobacteria (100)	Gammaproteobacteria (100)	Pseudomonadales (100)	Pseudomonadaceae (100)	*Azotobacter (87)*
OTU0010	Bacteria (100)	Proteobacteria (100)	Gammaproteobacteria (100)	Alteromonadales (100)	Alteromonadaceae (100)	Unclassified (<80)
OTU0011	Bacteria (100)	Proteobacteria (100)	Gammaproteobacteria (100)	Alteromonadales (100)	Alteromonadaceae (100)	*Alteromonas (87)*
OTU0012	Bacteria (100)	Proteobacteria (100)	Gammaproteobacteria (100)	Alteromonadales (87)	Shewanellaceae (83)	*Shewanella (83)*
OTU0013	Bacteria (100)	Proteobacteria (100)	Gammaproteobacteria (100)	Vibrionales (100)	Vibrionaceae (100)	Unclassified (<80)
OTU0014	Bacteria (100)	Proteobacteria (100)	Gammaproteobacteria (100)	Vibrionales (100)	Vibrionaceae (100)	Unclassified (<80)
OTU0015	Bacteria (100)	Proteobacteria (100)	Gammaproteobacteria (100)	Enterobacteriales (100)	Enterobacteriaceae (100)	*Escherichia/Shigell*[illegible] *(86)*
OTU0016	Bacteria (100)	Proteobacteria (100)	Gammaproteobacteria (100)	Enterobacteriales (100)	Enterobacteriaceae (100)	Unclassified (<80)
OTU0017	Bacteria (100)	Proteobacteria (100)	Gammaproteobacteria (100)	Enterobacteriales (100)	Enterobacteriaceae (100)	Unclassified (<80)
OTU0018	Bacteria (100)	Proteobacteria (100)	Gammaproteobacteria (100)	Enterobacteriales (100)	Enterobacteriaceae (100)	Unclassified (<80)
OTU0019	Bacteria (100)	Proteobacteria (100)	Gammaproteobacteria (100)	Enterobacteriales (100)	Enterobacteriaceae (100)	Unclassified (<80)
OTU0020	Bacteria (100)	Proteobacteria (100)	Gammaproteobacteria (100)	Enterobacteriales (100)	Enterobacteriaceae (100)	Unclassified (<80)
OTU0021	Bacteria (100)	Proteobacteria (100)	Gammaproteobacteria (100)	Enterobacteriales (100)	Enterobacteriaceae (100)	Unclassified (<80)
OTU0022	Bacteria (100)	Actinobacteria (100)	Actinobacteria (100)	Geodermatophilales (100)	Geodermatophilaceae (100)	*Blastococcus (99)*
OTU0023	Bacteria (100)	Actinobacteria (100)	Actinobacteria (100)	Micrococcales (100)	Microbacteriaceae (99)	Unclassified (<80)
OTU0024	Bacteria (100)	Actinobacteria (100)	Actinobacteria (100)	Micrococcales (100)	Microbacteriaceae (100)	*Microbacterium (100)*
OTU0025	Bacteria (100)	Actinobacteria (100)	Actinobacteria (100)	Micrococcales (100)	Micrococcaceae (98)	Unclassified (<80)
OTU0026	Bacteria (100)	Actinobacteria (100)	Actinobacteria (100)	Actinomycetales (100)	Actinomycetaceae (100)	*Actinomyces (100)*
OTU0027	Bacteria (100)	Actinobacteria (100)	Actinobacteria (100)	Actinomycetales (100)	Actinomycetaceae (100)	*Actinomyces (98)*
OTU0028	Bacteria (100)	Actinobacteria (100)	Actinobacteria (100)	Pseudonocardiales (100)	Pseudonocardiaceae (100)	*Pseudonocardia (93)*
OTU0029	Bacteria (100)	Actinobacteria (100)	Actinobacteria (100)	Pseudonocardiales (100)	Pseudonocardiaceae (100)	*Pseudonocardia (100)*
OTU0030	Bacteria (100)	Actinobacteria (100)	Actinobacteria (100)	Pseudonocardiales (100)	Pseudonocardiaceae (100)	*Saccharopolyspora (84)*
OTU0031	Bacteria (100)	Actinobacteria (100)	Actinobacteria (100)	Corynebacteriales (100)	Corynebacteriaceae (99)	*Corynebacterium (99)*

#OTU	DOMAIN (%)	PHYLUM (%)	CLASS/SUBCLASS (%)	ORDER (%)	FAMILY (%)	GENUS (%)
OTU0045	Bacteria (100)	Actinobacteria (100)	Actinobacteria (100)	Pseudonocardiales (100)	Pseudonocardiaceae (100)	*Pseudonocardia (93)*
OTU0046	Bacteria (100)	Proteobacteria (100)	Gammaproteobacteria (99)	Alteromonadales (89)	Shewanellaceae (89)	*Shewanella (87)*
OTU0047	Bacteria (100)	Firmicutes (100)	Bacilli (100)	Bacillales (100)	Paenibacillaceae 1 (100)	*Paenibacillus (99)*
OTU0048	Bacteria (100)	Firmicutes (100)	Bacilli (100)	Bacillales (98)	Unclassified (<80)	Unclassified (<80)
OTU0049	Bacteria (100)	Proteobacteria (99)	Gammaproteobacteria (87)	Unclassified (<80)	Unclassified (<80)	Unclassified (<80)
OTU0050	Bacteria (100)	Proteobacteria (100)	Gammaproteobacteria (100)	Oceanospirillales (100)	Halomonadaceae (100)	*Halomonas (95)*
OTU0051	Bacteria (100)	Firmicutes (100)	Bacilli (100)	Bacillales (99)	Bacillaceae 1 (87)	*Bacillus (84)*
OTU0052	Bacteria (100)	Firmicutes (100)	Bacilli (100)	Lactobacillales (100)	Carnobacteriaceae (100)	*Atopostipes (100)*
OTU0053	Bacteria (100)	Proteobacteria (100)	Alphaproteobacteria (100)	Rhodospirillales (100)	Acetobacteraceae (100)	*Roseomonas (100)*
OTU0054	Bacteria (100)	Proteobacteria (99)	Alphaproteobacteria (99)	Sphingomonadales (99)	Sphingomonadaceae (99)	*Sphingomonas (96)*
OTU0055	Bacteria (100)	Deinococcus-Thermus (100)	Deinococci (100)	Deinococcales (100)	Deinococcaceae (100)	*Deinococcus (100)*
OTU0056	Bacteria (100)	Proteobacteria (100)	Gammaproteobacteria (100)	Vibrionales (100)	Vibrionaceae (100)	*Vibrio (100)*
OTU0057	Bacteria (100)	Firmicutes (100)	Bacilli (100)	Lactobacillales (100)	Leuconostocaceae (100)	*Weissella (100)*
OTU0058	Bacteria (100)	Proteobacteria (100)	Gammaproteobacteria (100)	Pseudomonadales (100)	Moraxellaceae (100)	*Acinetobacter (100)*
OTU0059	Bacteria (100)	Proteobacteria (100)	Gammaproteobacteria (100)	Pseudomonadales (97)	Pseudomonadaceae (97)	Unclassified (<80)
OTU0060	Bacteria (100)	Proteobacteria (100)	Gammaproteobacteria (100)	Pseudomonadales (96)	Pseudomonadaceae (96)	Unclassified (<80)
OTU0061	Bacteria (100)	Proteobacteria (100)	Gammaproteobacteria (100)	Pseudomonadales (100)	Pseudomonadaceae (100)	Unclassified (<80)
OTU0062	Bacteria (100)	Firmicutes (100)	Bacilli (100)	Lactobacillales (98)	Enterococcaceae (98)	*Enterococcus (86)*
OTU0063	Bacteria (100)	Proteobacteria (100)	Betaproteobacteria (100)	Gallionellales (84)	Gallionellaceae (84)	*Gallionella (84)*
OTU0064	Bacteria (100)	Proteobacteria (100)	Alphaproteobacteria (100)	Sphingomonadales (100)	Sphingomonadaceae (100)	*Sphingomonas (100)*
OTU0065	Bacteria (100)	Firmicutes (100)	Bacilli (100)	Bacillales (100)	Staphylococcaceae (100)	*Staphylococcus (84)*
OTU0066	Bacteria (100)	Proteobacteria (100)	Gammaproteobacteria (100)	Oceanospirillales (100)	Halomonadaceae (100)	*Halomonas (84)*
OTU0067	Bacteria (100)	Proteobacteria (100)	Betaproteobacteria (100)	Burkholderiales (99)	Comamonadaceae (99)	*Rhodoferax (99)*
OTU0068	Bacteria (100)	Proteobacteria (100)	Betaproteobacteria (100)	Methylophilales (100)	Methylophilaceae (100)	*Methylophilus (100)*
OTU0069	Bacteria (100)	Actinobacteria (100)	Actinobacteria (100)	Bifidobacteriales (100)	Bifidobacteriaceae (100)	*Bifidobacterium (100)*
OTU0070	Bacteria (100)	Actinobacteria (100)	Actinobacteria (100)	Micrococcales (100)	Brevibacteriaceae (100)	*Brevibacterium (100)*
OTU0071	Bacteria (100)	Actinobacteria (100)	Actinobacteria (100)	Micrococcales (100)	Micrococcaceae (100)	Unclassified (<80)
OTU0072	Bacteria (100)	Actinobacteria (100)	Actinobacteria (100)	Micrococcales (100)	Dermabacteraceae (100)	*Brachybacterium (100)*
OTU0073	Bacteria (100)	Proteobacteria (100)	Betaproteobacteria (100)	Burkholderiales (100)	Comamonadaceae (100)	*Unclassified (<80)*
OTU0074	Bacteria (100)	Firmicutes (100)	Clostridia (100)	Clostridiales (100)	Clostridiaceae 1 (100)	*Clostridium (100) sensu stricto*
OTU0075	Bacteria (100)	Proteobacteria (100)	Alphaproteobacteria (100)	Rhodobacterales (98)	Rhodobacteraceae (98)	Unclassified (<80)
OTU0076	Bacteria (100)	Proteobacteria (100)	Gammaproteobacteria (100)	Chromatiales (99)	Chromatiaceae (99)	*Rheinheimera (99)*
OTU0077	Bacteria (100)	Proteobacteria (100)	Gammaproteobacteria (100)	Vibrionales (98)	Vibrionaceae (98)	Unclassified (<80)
OTU0078	Bacteria (100)	Proteobacteria (100)	Alphaproteobacteria (100)	Rhizobiales (100)	Methylobacteriaceae (100)	*Methylobacterium (98)*

#OTU	DOMAIN (%)	PHYLUM (%)	CLASS/SUBCLASS (%)	ORDER (%)	FAMILY (%)	GENUS (%)
OTU0092	Bacteria (100)	Proteobacteria (100)	Gammaproteobacteria (100)	Alteromonadales (100)	Shewanellaceae (100)	*Shewanella (100)*
OTU0093	Bacteria (100)	Proteobacteria (100)	Gammaproteobacteria (100)	Vibrionales (99)	Vibrionaceae (99)	Unclassified (<80)
OTU0094	Bacteria (100)	Firmicutes (100)	Bacilli (100)	Bacillales (99)	Planococcaceae (91)	Unclassified (<80)
OTU0095	Bacteria (100)	Deinococcus-Thermus (100)	Deinococci (100)	Thermales (100)	Thermaceae (100)	*Meiothermus (100)*
OTU0096	Bacteria (100)	Proteobacteria (100)	Gammaproteobacteria (100)	Enterobacteriales (100)	Enterobacteriaceae (100)	Unclassified (<80)
OTU0097	Bacteria (100)	Proteobacteria (100)	Gammaproteobacteria (100)	Alteromonadales (100)	Shewanellaceae (100)	*Shewanella (100)*
OTU0098	Bacteria (100)	Proteobacteria (100)	Gammaproteobacteria (100)	Pseudomonadales (100)	Moraxellaceae (100)	*Acinetobacter (100)*
OTU0099	Bacteria (100)	Actinobacteria (100)	Actinobacteria (100)	Micrococcales (100)	Microbacteriaceae (100)	*Microbacterium (86)*
OTU0100	Bacteria (100)	Proteobacteria (100)	Gammaproteobacteria (100)	Pseudomonadales (100)	Pseudomonadaceae (100)	*Pseudomonas (85)*
OTU0101	Bacteria (100)	Actinobacteria (100)	Actinobacteria (100)	Pseudonocardiales (100)	Pseudonocardiaceae (100)	*Amycolatopsis (99)*
OTU0102	Bacteria (100)	Proteobacteria (100)	Betaproteobacteria (100)	Hydrogenophilales (100)	Hydrogenophilaceae (100)	*Hydrogenophilus (100)*
OTU0103	Bacteria (100)	Firmicutes (100)	Bacilli (100)	Bacillales (100)	Unclassified (<80)	Unclassified (<80)
OTU0104	Bacteria (100)	Proteobacteria (100)	Betaproteobacteria (100)	Unclassified (<80)	Unclassified (<80)	Unclassified (<80)
OTU0105	Bacteria (100)	Proteobacteria (100)	Gammaproteobacteria (100)	Vibrionales (100)	Vibrionaceae (100)	*Aliivibrio (100)*
OTU0106	Bacteria (100)	Proteobacteria (100)	Alphaproteobacteria (100)	Rhodobacterales (100)	Rhodobacteraceae (100)	*Paracoccus (80)*
OTU0107	Bacteria (100)	Proteobacteria (100)	Gammaproteobacteria (100)	Pseudomonadales (100)	Pseudomonadaceae (100)	*Pseudomonas (97)*
OTU0108	Bacteria (100)	Proteobacteria (100)	Gammaproteobacteria (100)	Pseudomonadales (100)	Pseudomonadaceae (100)	*Pseudomonas (96)*
OTU0109	Bacteria (100)	Cyanobact./Chloroplast (100)	--------	--------	--------	--------
OTU0110	Bacteria (100)	Firmicutes (100)	Bacilli (100)	Bacillales (98)	Bacillaceae 1 (98)	Unclassified (<80)
OTU0111	Bacteria (100)	Proteobacteria (100)	Gammaproteobacteria (100)	Vibrionales (100)	Vibrionaceae (100)	*Aliivibrio (94)*
OTU0112	Bacteria (100)	Proteobacteria (100)	Gammaproteobacteria (100)	Xanthomonadales (100)	Xanthomonadaceae (100)	*Stenotrophomonas (99)*
OTU0113	Bacteria (100)	Firmicutes (100)	Bacilli (100)	Bacillales (100)	Paenibacillaceae 1 (100)	*Brevibacillus (100)*
OTU0114	Bacteria (100)	Acidobacteria (99)	Acidobacteria_Gp16 (98)	--------†	--------†	--------†
OTU0115	Bacteria (100)	Proteobacteria (100)	Gammaproteobacteria (100)	Pseudomonadales (96)	Pseudomonadaceae (96)	Unclassified (<80)
OTU0116	Bacteria (100)	Proteobacteria (100)	Gammaproteobacteria (100)	Pseudomonadales (92)	Pseudomonadaceae (92)	*Pseudomonas (88)*
OTU0117	Bacteria (100)	Proteobacteria (100)	Gammaproteobacteria (100)	Oceanospirillales (100)	Halomonadaceae (100)	*Halomonas (90)*
OTU0118	Bacteria (100)	Proteobacteria (100)	Gammaproteobacteria (100)	Enterobacteriales (100)	Enterobacteriaceae (100)	Unclassified (<80)
OTU0119	Bacteria (100)	Proteobacteria (100)	Gammaproteobacteria (100)	Pseudomonadales (100)	Pseudomonadaceae (100)	*Pseudomonas (100)*
OTU0120	Bacteria (100)	Actinobacteria (100)	Actinobacteria (100)	Pseudonocardiales (100)	Pseudonocardiaceae (100)	*Amycolatopsis (86)*
OTU0121	Bacteria (100)	Proteobacteria (100)	Gammaproteobacteria (100)	Enterobacteriales (100)	Enterobacteriaceae (100)	Unclassified (<80)
OTU0122	Bacteria (100)	Proteobacteria (100)	Gammaproteobacteria (100)	Enterobacteriales (100)	Enterobacteriaceae (100)	Unclassified (<80)
OTU0123	Bacteria (100)	Proteobacteria (100)	Gammaproteobacteria (100)	Pseudomonadales (100)	Pseudomonadaceae (100)	*Pseudomonas (100)*
OTU0124	Bacteria (100)	Proteobacteria (100)	Betaproteobacteria (100)	Methylophilales (100)	Methylophilaceae (100)	Unclassified (<80)
OTU0125	Bacteria (100)	Actinobacteria (100)	Actinobacteria (100)	Micrococcales (100)	Micrococcaceae (100)	*Arthrobacter (91)*

#OTU	DOMAIN (%)	PHYLUM (%)	CLASS/SUBCLASS (%)	ORDER (%)	FAMILY (%)	*GENUS (%)*
OTU0139	Bacteria (100)	Proteobacteria (100)	Alphaproteobacteria (100)	Rhodospirillales (100)	Acetobacteraceae (100)	*Roseomonas (98)*
OTU0140	Bacteria (100)	Firmicutes (100)	Bacilli (100)	Bacillales (100)	Unclassified (<80)	Unclassified (<80)
OTU0141	Bacteria (100)	Proteobacteria (100)	Alphaproteobacteria (100)	Rhodospirillales (100)	Acetobacteraceae (100)	*Belnapia (80)*
OTU0142	Bacteria (100)	Proteobacteria (100)	Alphaproteobacteria (100)	Sphingomonadales (100)	Sphingomonadaceae (100)	*Sphingomonas (99)*
OTU0143	Bacteria (100)	Actinobacteria (100)	Actinobacteria (100)	Micrococcales (100)	Micrococcaceae (99)	*Arthrobacter (94)*
OTU0144	Bacteria (100)	Proteobacteria (100)	Gammaproteobacteria (100)	Enterobacteriales (100)	Enterobacteriaceae (100)	Unclassified (<80)
OTU0145	Bacteria (100)	Actinobacteria (100)	Actinobacteria (100)	Micrococcales (100)	Micrococcaceae (96)	*Kocuria (91)*
OTU0146	Bacteria (100)	Proteobacteria (100)	Gammaproteobacteria (100)	Pseudomonadales (100)	Pseudomonadaceae (100)	*Pseudomonas (93)*
OTU0147	Bacteria (100)	Proteobacteria (100)	Gammaproteobacteria (100)	Pseudomonadales (100)	Pseudomonadaceae (100)	*Pseudomonas (97)*
OTU0148	Bacteria (100)	Actinobacteria (100)	Actinobacteria (100)	Micrococcales (100)	Microbacteriaceae (100)	Unclassified (<80)
OTU0149	Bacteria (100)	Proteobacteria (100)	Betaproteobacteria (100)	Burkholderiales (100)	Comamonadaceae (100)	*Hydrogenophaga (100)*
OTU0150	Bacteria (100)	Firmicutes (100)	Bacilli (100)	Bacillales (100)	Bacillaceae 2 (97)	Unclassified (<80)
OTU0151	Bacteria (100)	Proteobacteria (99)	Gammaproteobacteria (98)	Pseudomonadales (80)	Pseudomonadaceae (80)	Unclassified (<80)
OTU0152	Bacteria (100)	Proteobacteria (100)	Gammaproteobacteria (100)	Oceanospirillales (100)	Halomonadaceae (100)	*Halomonas (98)*
OTU0153	Bacteria (100)	Proteobacteria (100)	Gammaproteobacteria (100)	Alteromonadales (100)	Pseudoalteromonadaceae (100)	*Pseudoalteromonas (100)*
OTU0154	Bacteria (100)	Proteobacteria (99)	Gammaproteobacteria (89)	Unclassified (<80)	Unclassified (<80)	Unclassified (<80)
OTU0155	Bacteria (100)	Proteobacteria (100)	Gammaproteobacteria (100)	Alteromonadales (100)	Pseudoalteromonadaceae (100)	*Pseudoalteromonas (100)*
OTU0156	Bacteria (100)	Firmicutes (100)	Bacilli (100)	Bacillales (100)	Paenibacillaceae 1 (100)	*Brevibacillus (100)*
OTU0157	Bacteria (100)	Proteobacteria (100)	Gammaproteobacteria (100)	Xanthomonadales (100)	Xanthomonadaceae (100)	*Stenotrophomonas (99)*
OTU0158	Bacteria (100)	Proteobacteria (100)	Alphaproteobacteria (100)	Sphingomonadales (100)	Sphingomonadaceae (100)	*Sphingomonas (92)*
OTU0159	Bacteria (100)	Firmicutes (100)	Bacilli (100)	Bacillales (100)	Bacillaceae 1 (97)	*Bacillus (93)*
OTU0160	Bacteria (100)	Proteobacteria (100)	Gammaproteobacteria (100)	Chromatiales (99)	Chromatiaceae (99)	*Rheinheimera (99)*
OTU0161	Bacteria (100)	Proteobacteria (100)	Betaproteobacteria (100)	Burkholderiales (100)	Burkholderiaceae (100)	*Burkholderia (99)*
OTU0162	Bacteria (100)	Proteobacteria (100)	Gammaproteobacteria (100)	Oceanospirillales (100)	Halomonadaceae (100)	Unclassified (<80)
OTU0163	Bacteria (100)	Firmicutes (100)	Bacilli (100)	Bacillales (100)	Paenibacillaceae 1 (100)	*Paenibacillus (100)*
OTU0164	Bacteria (100)	Firmicutes (100)	Clostridia (100)	Clostridiales (100)	Ruminococcaceae (100)	*Ruminococcus (88)*
OTU0165	Bacteria (100)	Actinobacteria (100)	Actinobacteria (100)	Micrococcales (100)	Microbacteriaceae (100)	Unclassified (<80)
OTU0166	Bacteria (100)	Firmicutes (100)	Bacilli (100)	Bacillales (100)	Bacillales incertae sedis iXII (96)	*Exiguobacterium (96)*
OTU0167	Bacteria (100)	Actinobacteria (100)	Actinobacteria (100)	Micrococcales (100)	Micrococcaceae (100)	*Kocuria (99)*
OTU0168	Bacteria (100)	Proteobacteria (100)	Gammaproteobacteria (100)	Alteromonadales (99)	Shewanellaceae (99)	*Shewanella (99)*
OTU0169	Bacteria (100)	Proteobacteria (100)	Gammaproteobacteria (100)	Pseudomonadales (89)	Pseudomonadaceae (89)	Unclassified (<80)
OTU0170	Bacteria (100)	Proteobacteria (100)	Gammaproteobacteria (100)	Pseudomonadales (98)	Pseudomonadaceae (98)	Unclassified (<80)
OTU0171	Bacteria (100)	Firmicutes (100)	Bacilli (100)	Bacillales (100)	Staphylococcaceae (100)	*Staphylococcus (100)*
OTU0172	Bacteria (100)	Actinobacteria (100)	Actinobacteria (100)	Micrococcales (100)	Micrococcaceae (100)	*Micrococcus (89)*
[illegible]	Bacteria [illegible]	Firmicutes [illegible]	Bacilli [illegible]	Bacillales [illegible]	Unclassified [illegible]	Unclassified [illegible]

#OTU	DOMAIN (%)	PHYLUM (%)	CLASS/SUBCLASS (%)	ORDER (%)	FAMILY (%)	*GENUS (%)*
OTU0186	Bacteria (100)	Proteobacteria (100)	Gammaproteobacteria (100)	Alteromonadales (100)	Shewanellaceae (100)	*Shewanella (100)*
OTU0187	Bacteria (100)	Firmicutes (100)	Bacilli (99)	Bacillales (99)	Paenibacillaceae 1 (83)	Unclassified (<80)
OTU0188	Bacteria (100)	Firmicutes (100)	Bacilli (100)	Bacillales (99)	Staphylococcaceae (97)	*Staphylococcus (93)*
OTU0189	Bacteria (100)	Proteobacteria (100)	Gammaproteobacteria (100)	Unclassified (<80)	Unclassified (<80)	Unclassified (<80)
OTU0190	Bacteria (100)	Proteobacteria (100)	Gammaproteobacteria (100)	Enterobacteriales (100)	Enterobacteriaceae (100)	Unclassified (<80)
OTU0191	Bacteria (100)	Proteobacteria (100)	Gammaproteobacteria (100)	Enterobacteriales (100)	Enterobacteriaceae (100)	*Escherichia/Shigella (86)*
OTU0192	Bacteria (100)	Proteobacteria (100)	Alphaproteobacteria (100)	Rhodobacterales (99)	Rhodobacteraceae (99)	Unclassified (<80)
OTU0193	Bacteria (100)	Firmicutes (100)	Bacilli (100)	Bacillales (99)	Bacillaceae 2 (97)	*Oceanobacillus (97)*
OTU0194	Bacteria (100)	Firmicutes (100)	Bacilli (100)	Bacillales (98)	Unclassified (<80)	Unclassified (<80)
OTU0195	Bacteria (100)	Proteobacteria (100)	Gammaproteobacteria (100)	Unclassified (<80)	Unclassified (<80)	Unclassified (<80)
OTU0196	Bacteria (100)	Firmicutes (100)	Bacilli (100)	Bacillales (96)	Unclassified (<80)	Unclassified (<80)
OTU0197	Bacteria (100)	Proteobacteria (100)	Gammaproteobacteria (100)	Enterobacteriales (97)	Enterobacteriaceae (97)	Unclassified (<80)
OTU0198	Bacteria (100)	Proteobacteria (100)	Gammaproteobacteria (100)	Vibrionales (100)	Vibrionaceae (100)	Unclassified (<80)
OTU0199	Bacteria (100)	Proteobacteria (100)	Epsilonproteobacteria (100)	Campylobacterales (100)	Helicobacteraceae (100)	*Sulfuricurvum (99)*
OTU0200	Bacteria (100)	Proteobacteria (100)	Gammaproteobacteria (100)	Xanthomonadales (100)	Xanthomonadaceae (100)	*Stenotrophomonas (96)*
OTU0201	Bacteria (100)	Proteobacteria (100)	Gammaproteobacteria (100)	Enterobacteriales (100)	Enterobacteriaceae (100)	*Escherichia/Shigella (83)*
OTU0202	Bacteria (100)	Proteobacteria (100)	Gammaproteobacteria (100)	Enterobacteriales (100)	Enterobacteriaceae (100)	Unclassified (<80)
OTU0203	Bacteria (100)	Firmicutes (100)	Bacilli (100)	Bacillales (100)	Staphylococcaceae (100)	*Staphylococcus (100)*
OTU0204	Bacteria (100)	Firmicutes (98)	Negativicutes (96)	Selenomonadales (96)	Veillonellaceae (96)	*Dialister (93)*
OTU0205	Bacteria (100)	Actinobacteria (100)	Actinobacteria (100)	Corynebacteriales (100)	Dietziaceae (99)	*Dietzia (99)*
OTU0206	Bacteria (100)	Proteobacteria (100)	Deltaproteobacteria (100)	Bdellovibrionales (100)	Bacteriovoracaceae (100)	*Peredibacter (100)*
OTU0207	Bacteria (100)	Proteobacteria (100)	Gammaproteobacteria (100)	Pseudomonadales (97)	Pseudomonadaceae (97)	*Pseudomonas (82)*
OTU0208	Bacteria (100)	Firmicutes (100)	Bacilli (100)	Bacillales (99)	Planococcaceae (95)	Unclassified (<80)
OTU0209	Bacteria (100)	Proteobacteria (100)	Gammaproteobacteria (100)	Alteromonadales (100)	Shewanellaceae (100)	*Shewanella (100)*
OTU0210	Bacteria (100)	Proteobacteria (100)	Gammaproteobacteria (100)	Alteromonadales (100)	Alteromonadaceae (100)	*Alteromonas (96)*
OTU0211	Bacteria (100)	Firmicutes (100)	Bacilli (100)	Bacillales (100)	Bacillaceae 1 (88)	Unclassified (<80)
OTU0212	Bacteria (100)	Firmicutes (100)	Bacilli (100)	Lactobacillales (100)	Streptococcaceae (100)	*Streptococcus (100)*
OTU0213	Bacteria (100)	Firmicutes (100)	Bacilli (100)	Bacillales (98)	Unclassified (<80)	Unclassified (<80)
OTU0214	Bacteria (100)	Actinobacteria (100)	Actinobacteria (100)	Micrococcales (100)	Micrococcaceae (100)	*Citricoccus (82)*
OTU0215	Bacteria (100)	Proteobacteria (100)	Gammaproteobacteria (100)	Pseudomonadales (100)	Moraxellaceae (100)	*Acinetobacter (100)*
OTU0216	Bacteria (100)	Actinobacteria (100)	Actinobacteria (100)	Micrococcales (100)	Micrococcaceae (100)	*Arthrobacter (99)*
OTU0217	Bacteria (100)	Proteobacteria (100)	Gammaproteobacteria (100)	Xanthomonadales (100)	Xanthomonadaceae (100)	*Xanthomonas (100)*
OTU0218	Bacteria (100)	Proteobacteria (100)	Gammaproteobacteria (100)	Alteromonadales (100)	Alteromonadaceae (100)	Unclassified (<80)
OTU0219	Bacteria (100)	Proteobacteria (100)	Gammaproteobacteria (100)	Vibrionales (99)	Vibrionaceae (99)	Unclassified (<80)
[illegible]	Bacteria [illegible]	Proteobacteria [illegible]	Gammaproteobacteria [illegible]	Pseudomonadales [illegible]	Pseudomonadaceae [illegible]	Unclassified [illegible]

#OTU	DOMAIN (%)	PHYLUM (%)	CLASS/SUBCLASS (%)	ORDER (%)	FAMILY (%)	*GENUS (%)*
OTU0234	Bacteria (100)	Proteobacteria (100)	Alphaproteobacteria (100)	Rhizobiales (100)	Rhizobiaceae (99)	*Neorhizobium (99)*
OTU0235	Bacteria (100)	Proteobacteria (100)	Gammaproteobacteria (100)	Pseudomonadales (93)	Pseudomonadaceae (93)	Unclassified (<80)
OTU0236	Bacteria (100)	Firmicutes (100)	Clostridia (100)	Clostridiales (100)	Clostridiaceae 1 (100)	*Clostridium sensu st (97)*
OTU0237	Bacteria (100)	Proteobacteria (100)	Alphaproteobacteria (100)	Rhizobiales (100)	Rhizobiaceae (97)	*Neorhizobium (94)*
OTU0238	Bacteria (100)	Proteobacteria (100)	Gammaproteobacteria (99)	Pseudomonadales (89)	Pseudomonadaceae (89)	Unclassified (<80)
OTU0239	Bacteria (100)	Firmicutes (100)	Erysipelotrichia (100)	Erysipelotrichales (100)	Erysipelotrichaceae (100)	*Erysipelothrix (100)*
OTU0240	Bacteria (100)	Firmicutes (100)	Bacilli (100)	Lactobacillales (99)	Carnobacteriaceae (93)	*Granulicatella (81)*
OTU0241	Bacteria (100)	Proteobacteria (100)	Betaproteobacteria (100)	Neisseriales (100)	Neisseriaceae (100)	Unclassified (<80)
OTU0242	Bacteria (100)	Proteobacteria (100)	Gammaproteobacteria (100)	Enterobacteriales (100)	Enterobacteriaceae (100)	Unclassified (<80)
OTU0243	Bacteria (100)	Firmicutes (100)	Bacilli (100)	Bacillales (99)	Bacillaceae 1 (86)	*Bacillus (82)*
OTU0244	Bacteria (100)	Proteobacteria (100)	Alphaproteobacteria (99)	Sphingomonadales (99)	Sphingomonadaceae (99)	*Sphingomonas (84)*
OTU0245	Bacteria (100)	Firmicutes (100)	Bacilli (100)	Bacillales (87)	Unclassified (<80)	Unclassified (<80)
OTU0246	Bacteria (100)	Proteobacteria (100)	Gammaproteobacteria (100)	Enterobacteriales (100)	Enterobacteriaceae (100)	Unclassified (<80)
OTU0247	Bacteria (100)	Proteobacteria (100)	Deltaproteobacteria (100)	Desulfobacterales (100)	Desulfobulbaceae (100)	*Desulfopila (92)*
OTU0248	Bacteria (100)	Proteobacteria (100)	Alphaproteobacteria (100)	Unclassified (<80)	Unclassified (<80)	*Unclassified (<80)*
OTU0249	Bacteria (100)	Firmicutes (100)	Bacilli (100)	Lactobacillales (100)	Enterococcaceae (100)	*Enterococcus (100)*
OTU0250	Bacteria (100)	Firmicutes (100)	Bacilli (100)	Lactobacillales (94)	Enterococcaceae (88)	*Enterococcus (88)*
OTU0251	Bacteria (100)	Proteobacteria (100)	Alphaproteobacteria (100)	Rhizobiales (100)	Bradyrhizobiaceae (99)	*Bosea (99)*
OTU0252	Bacteria (100)	Firmicutes (100)	Bacilli (100)	Bacillales (100)	Staphylococcaceae (100)	*Staphylococcus (100)*
OTU0253	Bacteria (100)	Proteobacteria (100)	Gammaproteobacteria (100)	Xanthomonadales (100)	Xanthomonadaceae (100)	*Stenotrophomonas (100)*
OTU0254	Bacteria (100)	Proteobacteria (100)	Gammaproteobacteria (100)	Vibrionales (100)	Vibrionaceae (100)	*Lucibacterium (83)*
OTU0255	Bacteria (100)	Proteobacteria (100)	Gammaproteobacteria (100)	Vibrionales (100)	Vibrionaceae (100)	*Lucibacterium (82)*
OTU0256	Bacteria (100)	Proteobacteria (100)	Gammaproteobacteria (100)	Enterobacteriales (100)	Enterobacteriaceae (100)	Unclassified (<80)
OTU0257	Bacteria (100)	Proteobacteria (100)	Gammaproteobacteria (100)	Oceanospirillales (100)	Halomonadaceae (100)	*Halomonas (95)*
OTU0258	Bacteria (100)	Proteobacteria (100)	Betaproteobacteria (100)	Burkholderiales (100)	Comamonadaceae (100)	*Acidovorax (95)*
OTU0259	Bacteria (100)	Proteobacteria (100)	Betaproteobacteria (100)	Rhodocyclales (97)	Rhodocyclaceae (97)	*Unclassified (<80)*
OTU0260	Bacteria (100)	Proteobacteria (100)	Betaproteobacteria (100)	Gallionellales (92)	Gallionellaceae (92)	*Sideroxydans (92)*
OTU0261	Bacteria (100)	Actinobacteria (100)	Actinobacteria (100)	Micrococcales (100)	Cellulomonadaceae (98)	*Cellulomonas (97)*
OTU0262	Bacteria (100)	Firmicutes (100)	Bacilli (95)	Lactobacillales (95)	Leuconostocaceae (91)	Unclassified (<80)
OTU0263	Bacteria (100)	Firmicutes (100)	Bacilli (100)	Lactobacillales (100)	Aerococcaceae (100)	*Aerococcus (99)*
OTU0264	Bacteria (100)	Proteobacteria (100)	Gammaproteobacteria (100)	Vibrionales (100)	Vibrionaceae (100)	Unclassified (<80)
OTU0265	Bacteria (100)	Firmicutes (100)	Bacilli (100)	Bacillales (99)	Planococcaceae (97)	Unclassified (<80)
OTU0266	Bacteria (100)	Proteobacteria (100)	Gammaproteobacteria (100)	Enterobacteriales (100)	Enterobacteriaceae (100)	Unclassified (<80)
OTU0267	Bacteria (100)	Actinobacteria (100)	Actinobacteria (94)	Gaiellales (84)	Gaiellaceae (84)	*Gaiella (84)*
	Bacteria	Actinobacteria	Actinobacteria	Micrococcales	Bogoriellaceae	*Georgenia*

#OTU	DOMAIN (%)	PHYLUM (%)	CLASS/SUBCLASS (%)	ORDER (%)	FAMILY (%)	*GENUS (%)*
OTU0282	Bacteria (100)	Proteobacteria (100)	Gammaproteobacteria (100)	Pseudomonadales (100)	Pseudomonadaceae (100)	Unclassified (<80)
OTU0283	Bacteria (100)	Firmicutes (100)	Bacilli (100)	Bacillales (100)	Bacillaceae 1 (84)	Unclassified (<80)
OTU0284	Bacteria (100)	Firmicutes (100)	Bacilli (100)	Lactobacillales (100)	Leuconostocaceae (100)	*Leuconostoc (100)*
OTU0285	Bacteria (100)	Firmicutes (100)	Bacilli (100)	Bacillales (100)	Planococcaceae (92)	Unclassified (<80)
OTU0286	Bacteria (100)	Proteobacteria (100)	Gammaproteobacteria (100)	Xanthomonadales (100)	Xanthomonadaceae (100)	*Pseudoxanthomonas (100)*
OTU0287	Bacteria (100)	Firmicutes (100)	Bacilli (100)	Bacillales (100)	Planococcaceae (99)	*Sporosarcina (87)*
OTU0288	Bacteria (100)	Proteobacteria (100)	Gammaproteobacteria (100)	Enterobacteriales (100)	Enterobacteriaceae (100)	*Salmonella (100)*
OTU0289	Bacteria (100)	Actinobacteria (100)	Actinobacteria (100)	Streptomycetales (100)	Streptomycetaceae (92)	Unclassified (<80)
OTU0290	Bacteria (100)	Firmicutes (100)	Bacilli (100)	Bacillales (95)	Unclassified (<80)	Unclassified (<80)
OTU0291	Bacteria (100)	Actinobacteria (100)	Actinobacteria (100)	Frankiales (100)	Unclassified (<80)	Unclassified (<80)
OTU0292	Bacteria (100)	Proteobacteria (100)	Betaproteobacteria (97)	Burkholderiales (97)	Comamonadaceae (96)	Unclassified (<80)
OTU0293	Bacteria (100)	Firmicutes (100)	Bacilli (100)	Lactobacillales (100)	Aerococcaceae (100)	*Aerococcus (100)*
OTU0294	Bacteria (100)	Actinobacteria (100)	Actinobacteria (100)	Micrococcales (100)	Micrococcaceae (100)	*Kocuria (98)*
OTU0295	Bacteria (100)	Proteobacteria (100)	Gammaproteobacteria (100)	Enterobacteriales (100)	Enterobacteriaceae (100)	Unclassified (<80)
OTU0296	Bacteria (100)	Proteobacteria (100)	Alphaproteobacteria (100)	Sphingomonadales (100)	Sphingomonadaceae (99)	*Sphingobium (99)*
OTU0297	Bacteria (100)	Actinobacteria (100)	Actinobacteria (100)	Micrococcales (100)	Micrococcaceae (100)	Unclassified (<80)
OTU0298	Bacteria (100)	Actinobacteria (100)	Actinobacteria (100)	Propionibacteriales (100)	Nocardiopsaceae (100)	*Nocardiopsis (92)*
OTU0299	Bacteria (100)	Firmicutes (100)	Bacilli (100)	Lactobacillales (100)	Unclassified (<80)	Unclassified (<80)
OTU0300	Bacteria (100)	Actinobacteria (100)	Actinobacteria (100)	Pseudonocardiales (100)	Pseudonocardiaceae (100)	*Saccharopolyspora (96)*
OTU0301	Bacteria (100)	Actinobacteria (100)	Actinobacteria (100)	Pseudonocardiales (100)	Pseudonocardiaceae (99)	Unclassified (<80)
OTU0302	Bacteria (100)	Actinobacteria (100)	Actinobacteria (100)	Micrococcales (100)	Micrococcaceae (100)	Unclassified (<80)
OTU0303	Bacteria (100)	Proteobacteria (100)	Alphaproteobacteria (100)	Rhizobiales (100)	Methylobacteriaceae (100)	*Methylobacterium (100)*
OTU0304	Bacteria (100)	Firmicutes (100)	Bacilli (100)	Bacillales (98)	Bacillaceae 1 (98)	Unclassified (<80)
OTU0305	Bacteria (100)	Proteobacteria (100)	Gammaproteobacteria (100)	Enterobacteriales (100)	Enterobacteriaceae (100)	Unclassified (<80)
OTU0306	Bacteria (100)	Proteobacteria (100)	Alphaproteobacteria (100)	Sphingomonadales (100)	Sphingomonadaceae (100)	*Sphingomonas (100)*
OTU0307	Bacteria (100)	Actinobacteria (99)	Actinobacteria (99)	Corynebacteriales (99)	Corynebacteriaceae (95)	*Corynebacterium (94)*
OTU0308	Bacteria (100)	Proteobacteria (100)	Alphaproteobacteria (100)	Caulobacterales (100)	Caulobacteraceae (100)	*Brevundimonas (100)*
OTU0309	Bacteria (100)	Proteobacteria (100)	Alphaproteobacteria (100)	Caulobacterales (100)	Caulobacteraceae (100)	*Brevundimonas (100)*
OTU0310	Bacteria (100)	Firmicutes (100)	Bacilli (100)	Bacillales (97)	Unclassified (<80)	Unclassified (<80)
OTU0311	Bacteria (100)	Firmicutes (100)	Clostridia (100)	Clostridiales (100)	Peptostreptococcaceae (87)	Unclassified (<80)
OTU0312	Bacteria (100)	Actinobacteria (100)	Actinobacteria (100)	Micrococcales (100)	Microbacteriaceae (100)	*Microbacterium (95)*
OTU0313	Bacteria (100)	Firmicutes (100)	Bacilli (100)	Bacillales (100)	Unclassified (<80)	Unclassified (<80)
OTU0314	Bacteria (100)	Actinobacteria (100)	Actinobacteria (100)	Micrococcales (100)	Brevibacteriaceae (99)	*Brevibacterium (97)*
OTU0315	Bacteria (100)	Cyanobact./Chloroplast (100)	----------	----------	----------	----------

#OTU	DOMAIN (%)	PHYLUM (%)	CLASS/SUBCLASS (%)	ORDER (%)	FAMILY (%)	*GENUS (%)*
OTU0329	Bacteria (100)	Proteobacteria (100)	Gammaproteobacteria (100)	Oceanospirillales (100)	Halomonadaceae (100)	*Halomonas (100)*
OTU0330	Bacteria (100)	Proteobacteria (100)	Gammaproteobacteria (100)	Pseudomonadales (100)	Pseudomonadaceae (100)	*Pseudomonas (83)*
OTU0331	Bacteria (100)	Bacteroidetes (100)	Bacteroidia (83)	Bacteroidales (83)	Unclassified (<80)	Unclassified (<80)
OTU0332	Bacteria (100)	Proteobacteria (100)	Gammaproteobacteria (100)	Pseudomonadales (100)	Pseudomonadaceae (100)	*Pseudomonas (97)*
OTU0333	Bacteria (100)	Actinobacteria (100)	Actinobacteria (100)	Micrococcales (100)	Micrococcaceae (100)	*Arthrobacter (90)*
OTU0334	Bacteria (100)	Proteobacteria (100)	Gammaproteobacteria (100)	Pseudomonadales (97)	Pseudomonadaceae (97)	*Pseudomonas (81)*
OTU0335	Bacteria (100)	Actinobacteria (100)	Actinobacteria (100)	Micrococcales (100)	Micrococcaceae (100)	*Arthrobacter (84)*
OTU0336	Bacteria (100)	Actinobacteria (100)	Actinobacteria (100)	Micrococcales (100)	Micrococcaceae (100)	*Kocuria (100)*
OTU0337	Bacteria (100)	Proteobacteria (100)	Gammaproteobacteria (100)	Pseudomonadales (99)	Pseudomonadaceae (99)	*Pseudomonas (96)*
OTU0338	Bacteria (100)	Proteobacteria (100)	Gammaproteobacteria (100)	Pseudomonadales (96)	Pseudomonadaceae (96)	Unclassified (<80)
OTU0339	Bacteria (100)	Proteobacteria (100)	Gammaproteobacteria (100)	Enterobacteriales (100)	Enterobacteriaceae (100)	Unclassified (<80)
OTU0340	Bacteria (100)	Firmicutes (100)	Bacilli (89)	Bacillales (89)	Unclassified (<80)	Unclassified (<80)
OTU0341	Bacteria (100)	Firmicutes (100)	Bacilli (100)	Bacillales (100)	Bacillaceae 2 (100)	*Geomicrobium (98)*
OTU0342	Bacteria (100)	Proteobacteria (100)	Gammaproteobacteria (100)	Enterobacteriales (100)	Enterobacteriaceae (100)	*Yersinia (82)*
OTU0343	Bacteria (100)	Proteobacteria (100)	Gammaproteobacteria (100)	Aeromonadales (100)	Aeromonadaceae (100)	*Zobellella (97)*
OTU0344	Bacteria (100)	Proteobacteria (100)	Gammaproteobacteria (100)	Xanthomonadales (100)	Xanthomonadaceae (100)	*Stenotrophomonas (99)*
OTU0345	Bacteria (100)	Proteobacteria (100)	Alphaproteobacteria (100)	Sphingomonadales (100)	Sphingomonadaceae (100)	*Sphingobium (98)*
OTU0346	Bacteria (100)	Firmicutes (100)	Bacilli (99)	Bacillales (99)	Unclassified (<80)	Unclassified (<80)
OTU0347	Bacteria (100)	Proteobacteria (100)	Gammaproteobacteria (100)	Xanthomonadales (100)	Xanthomonadaceae (100)	*Stenotrophomonas (100)*
OTU0348	Bacteria (100)	Proteobacteria (100)	Gammaproteobacteria (100)	Pseudomonadales (100)	Moraxellaceae (100)	*Psychrobacter (99)*
OTU0349	Bacteria (100)	Proteobacteria (100)	Gammaproteobacteria (100)	Alteromonadales (95)	Shewanellaceae (94)	*Shewanella (94)*
OTU0350	Bacteria (100)	Firmicutes (100)	Bacilli (100)	Lactobacillales (100)	Carnobacteriaceae (99)	*Carnobacterium (97)*
OTU0351	Bacteria (100)	Firmicutes (100)	Bacilli (100)	Bacillales (100)	Unclassified (<80)	Unclassified (<80)
OTU0352	Bacteria (100)	Actinobacteria (100)	Actinobacteria (100)	Micrococcales (100)	Brevibacteriaceae (100)	*Brevibacterium (100)*
OTU0353	Bacteria (100)	Actinobacteria (100)	Actinobacteria (100)	Propionibacteriales (100)	Nocardioidaceae (100)	*Actinopolymorpha (100)*
OTU0354	Bacteria (100)	Proteobacteria (100)	Alphaproteobacteria (100)	Sphingomonadales (100)	Sphingomonadaceae (100)	*Polymorphobacter (99)*
OTU0355	Bacteria (100)	Proteobacteria (100)	Betaproteobacteria (100)	Rhodocyclales (100)	Rhodocyclaceae (100)	*Thauera (95)*
OTU0356	Bacteria (100)	Proteobacteria (100)	Betaproteobacteria (100)	Burkholderiales (84)	Unclassified (<80)	Unclassified (<80)
OTU0357	Bacteria (100)	Proteobacteria (100)	Gammaproteobacteria (100)	Pseudomonadales (98)	Pseudomonadaceae (98)	*Pseudomonas (88)*
OTU0358	Bacteria (100)	Actinobacteria (100)	Actinobacteria (100)	Propionibacteriales (100)	Propionibacteriaceae (100)	*Luteococcus (87)*
OTU0359	Bacteria (100)	Proteobacteria (100)	Gammaproteobacteria (100)	Alteromonadales (100)	Idiomarinaceae (100)	*Idiomarina (100)*
OTU0360	Bacteria (100)	Proteobacteria (100)	Betaproteobacteria (100)	Burkholderiales (100)	Comamonadaceae (99)	*Comamonas (94)*
OTU0361	Bacteria (100)	Actinobacteria (100)	Actinobacteria (100)	Corynebacteriales (100)	Corynebacteriaceae (100)	*Corynebacterium (100)*

#OTU	DOMAIN (%)	PHYLUM (%)	CLASS/SUBCLASS (%)	ORDER (%)	FAMILY (%)	*GENUS (%)*
OTU0375	Bacteria (100)	Actinobacteria (100)	Actinobacteria (100)	Propionibacteriales (100)	Nocardiopsaceae (98)	Unclassified (<80)
OTU0376	Bacteria (100)	Proteobacteria (100)	Gammaproteobacteria (100)	Xanthomonadales (100)	Xanthomonadaceae (100)	*Stenotrophomonas (100)*
OTU0377	Bacteria (100)	Proteobacteria (100)	Gammaproteobacteria (100)	Enterobacteriales (100)	Enterobacteriaceae (100)	*Escherichia/Shigella (87)*
OTU0378	Bacteria (100)	Firmicutes (100)	Bacilli (100)	Bacillales (99)	Unclassified (<80)	Unclassified (<80)
OTU0379	Bacteria (100)	Firmicutes (100)	Clostridia (100)	Clostridiales (100)	Lachnospiraceae (100)	Unclassified (<80)
OTU0380	Bacteria (100)	Proteobacteria (100)	Alphaproteobacteria (100)	Rhodospirillales (100)	Rhodospirillaceae (100)	*Thalassospira (100)*
OTU0381	Bacteria (100)	Proteobacteria (100)	Gammaproteobacteria (100)	Enterobacteriales (100)	Enterobacteriaceae (100)	*Escherichia/Shigella (89)*
OTU0382	Bacteria (100)	Verrucomicrobia (99)	Opitutae (95)	Unclassified (<80)	Unclassified (<80)	Unclassified (<80)
OTU0383	Bacteria (100)	Bacteroidetes (100)	Sphingobacteriia (100)	Sphingobacteriales (100)	Sphingobacteriaceae (100)	*Pedobacter (92)*
OTU0384	Bacteria (100)	Firmicutes (100)	Clostridia (100)	Clostridiales (100)	Peptostreptococcaceae (100)	*RombOTUsia (100)*
OTU0385	Bacteria (100)	Proteobacteria (100)	Betaproteobacteria (98)	Unclassified (<80)	Unclassified (<80)	Unclassified (<80)
OTU0386	Bacteria (100)	Proteobacteria (100)	Alphaproteobacteria (100)	Sphingomonadales (100)	Sphingomonadaceae (100)	*Sphingomonas (85)*
OTU0387	Bacteria (100)	Proteobacteria (100)	Alphaproteobacteria (100)	Sphingomonadales (99)	Sphingomonadaceae (99)	*Sphingomonas (97)*
OTU0388	Bacteria (100)	Proteobacteria (100)	Alphaproteobacteria (100)	Sphingomonadales (100)	Sphingomonadaceae (100)	Unclassified (<80)
OTU0389	Bacteria (100)	Proteobacteria (100)	Gammaproteobacteria (100)	Pseudomonadales (100)	Moraxellaceae (100)	Acinetobacter (98)
OTU0390	Bacteria (100)	Proteobacteria (100)	Gammaproteobacteria (100)	Oceanospirillales (100)	Halomonadaceae (100)	*Halomonas (99)*
OTU0391	Bacteria (100)	Proteobacteria (100)	Alphaproteobacteria (100)	Rhizobiales (100)	Methylobacteriaceae (100)	*Methylobacterium (99)*
OTU0392	Bacteria (100)	Proteobacteria (99)	Alphaproteobacteria (99)	Sphingomonadales (96)	Sphingomonadaceae (95)	*Sphingomonas (88)*
OTU0393	Bacteria (100)	Proteobacteria (100)	Alphaproteobacteria (100)	Sphingomonadales (100)	Sphingomonadaceae (100)	Unclassified (<80)
OTU0394	Bacteria (100)	Proteobacteria (100)	Gammaproteobacteria (100)	Pseudomonadales (100)	Pseudomonadaceae (100)	Unclassified (<80)
OTU0395	Bacteria (100)	Actinobacteria (100)	Actinobacteria (100)	Bifidobacteriales (100)	Bifidobacteriaceae (100)	*Bifidobacterium (99)*
OTU0396	Bacteria (100)	Proteobacteria (100)	Gammaproteobacteria (100)	Xanthomonadales (100)	Xanthomonadaceae (100)	*Stenotrophomonas (87)*
OTU0397	Bacteria (100)	Proteobacteria (100)	Alphaproteobacteria (100)	Sphingomonadales (100)	Sphingomonadaceae (100)	Unclassified (<80)
OTU0398	Bacteria (100)	Proteobacteria (100)	Gammaproteobacteria (100)	Pseudomonadales (96)	Pseudomonadaceae (96)	Unclassified (<80)
OTU0399	Bacteria (100)	Proteobacteria (100)	Gammaproteobacteria (100)	Enterobacteriales (100)	Enterobacteriaceae (100)	*Cronobacter (93)*
OTU0400	Bacteria (100)	Actinobacteria (100)	Actinobacteria (100)	Pseudonocardiales (100)	Pseudonocardiaceae (100)	Unclassified (<80)
OTU0401	Bacteria (100)	Proteobacteria (100)	Alphaproteobacteria (100)	Caulobacterales (100)	Caulobacteraceae (100)	*Brevundimonas (100)*
OTU0402	Bacteria (100)	Proteobacteria (100)	Gammaproteobacteria (100)	Alteromonadales (100)	Shewanellaceae (100)	*Shewanella (100)*
OTU0403	Bacteria (100)	Proteobacteria (100)	Alphaproteobacteria (100)	Sphingomonadales (100)	Sphingomonadaceae (100)	*Sphingomonas (92)*
OTU0404	Bacteria (100)	Proteobacteria (100)	Gammaproteobacteria (100)	Enterobacteriales (100)	Enterobacteriaceae (100)	*Escherichia/Shigella (87)*
OTU0405	Bacteria (100)	Proteobacteria (100)	Alphaproteobacteria (100)	Rhodobacterales (100)	Rhodobacteraceae (100)	Unclassified (<80)
OTU0406	Bacteria (100)	Proteobacteria (100)	Alphaproteobacteria (100)	Rhizobiales (100)	Hyphomicrobiaceae (100)	*Devosia (100)*
OTU0407	Bacteria (100)	Proteobacteria (100)	Gammaproteobacteria (100)	Pseudomonadales (100)	Moraxellaceae (100)	*Acinetobacter (100)*
OTU0408	Bacteria (100)	Firmicutes (100)	Clostridia (100)	Clostridiales (100)	Peptostreptococcaceae (100)	*RombOTUsia (99)*
[illegible]	Bacteria [illegible]	Proteobacteria [illegible]	Betaproteobacteria [illegible]	Burkholderiales [illegible]	Oxalobacteraceae [illegible]	Unclassified [illegible]

#OTU	DOMAIN (%)	PHYLUM (%)	CLASS/SUBCLASS (%)	ORDER (%)	FAMILY (%)	*GENUS (%)*
OTU0422	Bacteria (100)	Firmicutes (100)	Bacilli (100)	Bacillales (100)	Planococcaceae (80)	Unclassified (<80)
OTU0423	Bacteria (100)	Acidobacteria (100)	Acidobacteria_Gp3 (100)	--------†	--------†	--------†
OTU0424	Bacteria (100)	Proteobacteria (100)	Gammaproteobacteria (100)	Enterobacteriales (91)	Enterobacteriaceae (91)	Unclassified (<80)
OTU0425	Bacteria (100)	Firmicutes (100)	Bacilli (100)	Bacillales (100)	Planococcaceae (99)	Unclassified (<80)
OTU0426	Bacteria (100)	Proteobacteria (100)	Betaproteobacteria (100)	Burkholderiales (97)	Burkholderiaceae (95)	*Burkholderia (93)*
OTU0427	Bacteria (100)	Proteobacteria (100)	Gammaproteobacteria (100)	Pseudomonadales (100)	Moraxellaceae (100)	*Acinetobacter (100)*
OTU0428	Bacteria (100)	Deinococcus-Thermus (100)	Deinococci (100)	Deinococcales (100)	Deinococcaceae (100)	*Deinococcus (100)*
OTU0429	Bacteria (100)	Proteobacteria (100)	Alphaproteobacteria (100)	Rhodospirillales (100)	Acetobacteraceae (100)	Unclassified (<80)
OTU0430	Bacteria (100)	Proteobacteria (100)	Betaproteobacteria (100)	Unclassified (<80)	Unclassified (<80)	Unclassified (<80)
OTU0431	Bacteria (100)	Actinobacteria (100)	Actinobacteria (100)	Corynebacteriales (100)	Unclassified (<80)	Unclassified (<80)
OTU0432	Bacteria (100)	Firmicutes (100)	Bacilli (100)	Bacillales (98)	Unclassified (<80)	Unclassified (<80)
OTU0433	Bacteria (100)	Proteobacteria (100)	Gammaproteobacteria (100)	Pseudomonadales (99)	Pseudomonadaceae (99)	*Pseudomonas (93)*
OTU0434	Bacteria (100)	Actinobacteria (100)	Actinobacteria (100)	Corynebacteriales (100)	Corynebacteriaceae (96)	*Corynebacterium (94)*
OTU0435	Bacteria (100)	Actinobacteria (100)	Actinobacteria (100)	Micrococcales (100)	Microbacteriaceae (100)	Unclassified (<80)
OTU0436	Bacteria (100)	Proteobacteria (100)	Gammaproteobacteria (100)	Pseudomonadales (99)	Pseudomonadaceae (99)	*Pseudomonas (87)*
OTU0437	Bacteria (100)	Firmicutes (100)	Bacilli (100)	Bacillales (100)	Staphylococcaceae (100)	*Staphylococcus (100)*
OTU0438	Bacteria (100)	Actinobacteria (100)	Actinobacteria (100)	Micrococcales (100)	Microbacteriaceae (100)	Unclassified (<80)
OTU0439	Bacteria (100)	Proteobacteria (100)	Gammaproteobacteria (100)	Pseudomonadales (100)	Pseudomonadaceae (100)	*Pseudomonas (99)*
OTU0440	Bacteria (100)	Firmicutes (100)	Bacilli (100)	Bacillales (99)	Planococcaceae (99)	Unclassified (<80)
OTU0441	Bacteria (100)	Proteobacteria (100)	Gammaproteobacteria (100)	Alteromonadales (100)	Pseudoalteromonadace ae (100)	*Pseudoalteromonas (100)*
OTU0442	Bacteria (100)	Actinobacteria (100)	Actinobacteria (100)	Propionibacteriales (100)	Propionibacteriaceae (100)	*Propionibacterium (100)*
OTU0443	Bacteria (100)	Firmicutes (100)	Bacilli (83)	Unclassified (<80)	Unclassified (<80)	Unclassified (<80)
OTU0444	Bacteria (100)	Firmicutes (100)	Bacilli (100)	Bacillales (99)	Staphylococcaceae (97)	*Staphylococcus (89)*
OTU0445	Bacteria (100)	Proteobacteria (100)	Gammaproteobacteria (100)	Xanthomonadales (100)	Xanthomonadaceae (100)	*Stenotrophomonas (88)*
OTU0446	Bacteria (100)	Firmicutes (100)	Bacilli (100)	Bacillales (100)	Bacillales incertae sedis XII (100)	*Exiguobacterium (100)*
OTU0447	Bacteria (100)	Proteobacteria (100)	Gammaproteobacteria (100)	Enterobacteriales (100)	Enterobacteriaceae (100)	*Pantoea (94)*
OTU0448	Bacteria (100)	Actinobacteria (100)	Actinobacteria (100)	Corynebacteriales (100)	Corynebacteriaceae (99)	*Corynebacterium (99)*
OTU0449	Bacteria (100)	Proteobacteria (100)	Gammaproteobacteria (100)	Pseudomonadales (98)	Pseudomonadaceae (98)	*Pseudomonas (81)*
OTU0450	Bacteria (100)	Actinobacteria (100)	Actinobacteria (100)	Micrococcales (100)	Microbacteriaceae (100)	*Microbacterium (87)*
OTU0451	Bacteria (100)	Actinobacteria (100)	Actinobacteria (100)	Corynebacteriales (100)	Corynebacteriaceae (100)	*Corynebacterium (100)*
OTU0452	Bacteria (100)	Proteobacteria (100)	Gammaproteobacteria (100)	Enterobacteriales (100)	Enterobacteriaceae (100)	Unclassified (<80)
OTU0453	Bacteria (100)	Proteobacteria (100)	Alphaproteobacteria (100)	Sphingomonadales (97)	Sphingomonadaceae (97)	Unclassified (<80)
OTU0454	Bacteria (100)	Actinobacteria (100)	Actinobacteria (100)	Corynebacteriales (100)	Unclassified (<80)	Unclassified (<80)
OTU0455	Bacteria (100)	Actinobacteria (100)	Actinobacteria (100)	Pseudonocardiales (100)	Pseudonocardiaceae (100)	*Pseudonocardia (91)*
[illegible]	Bacteria [illegible]	Proteobacteria [illegible]	Alphaproteobacteria [illegible]	Rhizobiales [illegible]	Hyphomicrobiaceae [illegible]	*Devosia* [illegible]

#OTU	DOMAIN (%)	PHYLUM (%)	CLASS/SUBCLASS (%)	ORDER (%)	FAMILY (%)	*GENUS (%)*
OTU0470	Bacteria (100)	Firmicutes (100)	Bacilli (100)	Lactobacillales (99)	Aerococcaceae (83)	Unclassified (<80)
OTU0471	Bacteria (100)	Firmicutes (100)	Bacilli (100)	Lactobacillales (100)	Enterococcaceae (100)	*Enterococcus (83)*
OTU0472	Bacteria (100)	Actinobacteria (100)	Actinobacteria (100)	Micromonosporales (100)	Unclassified (<80)	Unclassified (<80)
OTU0473	Bacteria (100)	Firmicutes (100)	Bacilli (100)	Lactobacillales (95)	Unclassified (<80)	Unclassified (<80)
OTU0474	Bacteria (100)	Actinobacteria (100)	Actinobacteria (100)	Jiangellales (100)	Unclassified (<80)	Unclassified (<80)
OTU0475	Bacteria (100)	Proteobacteria (100)	Gammaproteobacteria (100)	Pseudomonadales (100)	Pseudomonadaceae (100)	Unclassified (<80)
OTU0476	Bacteria (100)	Proteobacteria (100)	Alphaproteobacteria (100)	Rhodobacterales (98)	Rhodobacteraceae (98)	*Paracoccus (91)*
OTU0477	Bacteria (100)	Actinobacteria (100)	Actinobacteria (100)	Micrococcales (99)	Dermabacteraceae (97)	Unclassified (<80)
OTU0478	Bacteria (100)	Actinobacteria (100)	Actinobacteria (100)	Micrococcales (99)	Micrococcaceae (96)	*Kocuria (88)*
OTU0479	Bacteria (100)	Actinobacteria (100)	Actinobacteria (100)	Micrococcales (100)	Micrococcaceae (99)	*Kocuria (87)*
OTU0480	Bacteria (100)	Actinobacteria (100)	Actinobacteria (100)	Micrococcales (100)	Brevibacteriaceae (100)	*Brevibacterium (100)*
OTU0481	Bacteria (100)	Proteobacteria (100)	Gammaproteobacteria (100)	Pseudomonadales (100)	Moraxellaceae (100)	*Acinetobacter (100)*
OTU0482	Bacteria (100)	Proteobacteria (100)	Gammaproteobacteria (100)	Pseudomonadales (98)	Pseudomonadaceae (98)	Unclassified (<80)
OTU0483	Bacteria (100)	Actinobacteria (100)	Actinobacteria (100)	Pseudonocardiales (100)	Pseudonocardiaceae (100)	*Actinoalloteichus (91)*
OTU0484	Bacteria (100)	Proteobacteria (100)	Gammaproteobacteria (100)	Enterobacteriales (100)	Enterobacteriaceae (100)	Unclassified (<80)
OTU0485	Bacteria (100)	Actinobacteria (100)	Actinobacteria (100)	Pseudonocardiales (100)	Pseudonocardiaceae (100)	*Actinomycetospora (100)*
OTU0486	Bacteria (100)	Proteobacteria (100)	Gammaproteobacteria (100)	Enterobacteriales (100)	Enterobacteriaceae (100)	*Pantoea (84)*
OTU0487	Bacteria (100)	Actinobacteria (100)	Actinobacteria (100)	Pseudonocardiales (100)	Pseudonocardiaceae (100)	*Actinokineospora (94)*
OTU0488	Bacteria (100)	Firmicutes (100)	Bacilli (100)	Bacillales (100)	Bacillales incertae sedis XII (99)	*Exiguobacterium (99)*
OTU0489	Bacteria (100)	Proteobacteria (100)	Gammaproteobacteria (100)	Vibrionales (96)	Vibrionaceae (96)	Unclassified (<80)
OTU0490	Bacteria (100)	Proteobacteria (100)	Gammaproteobacteria (100)	Pseudomonadales (100)	Pseudomonadaceae (100)	*Pseudomonas (93)*
OTU0491	Bacteria (100)	Firmicutes (100)	Bacilli (100)	Bacillales (100)	Planococcaceae (86)	Unclassified (<80)
OTU0492	Bacteria (100)	Proteobacteria (100)	Betaproteobacteria (100)	Methylophilales (100)	Methylophilaceae (100)	*Methylophilus (100)*
OTU0493	Bacteria (100)	Proteobacteria (100)	Gammaproteobacteria (100)	Enterobacteriales (100)	Enterobacteriaceae (100)	Unclassified (<80)
OTU0494	Bacteria (100)	Actinobacteria (100)	Actinobacteria (100)	Pseudonocardiales (100)	Pseudonocardiaceae (100)	*Saccharomonospora (98)*
OTU0495	Bacteria (100)	Firmicutes (100)	Bacilli (100)	Lactobacillales (100)	Enterococcaceae (97)	*Vagococcus (83)*
OTU0496	Bacteria (100)	Firmicutes (100)	Bacilli (100)	Lactobacillales (100)	Carnobacteriaceae (100)	*Alkalibacterium (100)*
OTU0497	Bacteria (100)	Proteobacteria (100)	Gammaproteobacteria (100)	Pseudomonadales (100)	Pseudomonadaceae (100)	*Pseudomonas (94)*
OTU0498	Bacteria (100)	Firmicutes (100)	Bacilli (100)	Bacillales (100)	Bacillaceae 1 (82)	*Bacillus (80)*
OTU0499	Bacteria (100)	Firmicutes (100)	Bacilli (100)	Bacillales (100)	Planococcaceae (80)	Unclassified (<80)
OTU0500	Bacteria (100)	Actinobacteria (100)	Actinobacteria (100)	Corynebacteriales (100)	Corynebacteriaceae (100)	*Corynebacterium (100)*
OTU0501	Bacteria (100)	Proteobacteria (100)	Epsilonproteobacteria (100)	Campylobacterales (100)	Campylobacteraceae (100)	*Arcobacter (100)*
OTU0502	Bacteria (100)	Proteobacteria (100)	Gammaproteobacteria (100)	Enterobacteriales (100)	Enterobacteriaceae (100)	Unclassified (<80)
OTU0503	Bacteria (100)	Actinobacteria (100)	Actinobacteria (100)	Micrococcales (100)	Microbacteriaceae (100)	Unclassified (<80)
[illegible]	Bacteria [illegible]	Firmicutes [illegible]	Bacilli [illegible]	Bacillales [illegible]	Planococcaceae [illegible]	*Lysinibacillus* [illegible]

#OTU	DOMAIN (%)	PHYLUM (%)	CLASS/SUBCLASS (%)	ORDER (%)	FAMILY (%)	*GENUS (%)*
OTU0515	Bacteria (100)	Firmicutes (100)	Bacilli (100)	Bacillales (100)	Bacillaceae 1 (95)	Unclassified (<80)
OTU0516	Bacteria (100)	Proteobacteria (100)	Gammaproteobacteria (100)	Vibrionales (100)	Vibrionaceae (100)	*Vibrio (90)*
OTU0517	Bacteria (100)	Proteobacteria (100)	Gammaproteobacteria (100)	Pseudomonadales (99)	Moraxellaceae (99)	*Acinetobacter (96)*
OTU0518	Bacteria (100)	Firmicutes (100)	Bacilli (99)	Bacillales (98)	Unclassified (<80)	Unclassified (<80)
OTU0519	Bacteria (100)	Proteobacteria (100)	Gammaproteobacteria (100)	Pseudomonadales (84)	Pseudomonadaceae (83)	Unclassified (<80)
OTU0520	Bacteria (100)	Actinobacteria (100)	Actinobacteria (100)	Pseudonocardiales (100)	Pseudonocardiaceae (100)	*Saccharopolyspora (93)*
OTU0521	Bacteria (100)	Actinobacteria (100)	Actinobacteria (100)	Micrococcales (100)	Microbacteriaceae (99)	Unclassified (<80)
OTU0522	Bacteria (100)	Actinobacteria (100)	Actinobacteria (100)	Micrococcales (100)	Micrococcaceae (100)	*Arthrobacter (88)*
OTU0523	Bacteria (100)	Proteobacteria (100)	Gammaproteobacteria (100)	Pseudomonadales (98)	Pseudomonadaceae (98)	*Pseudomonas (87)*
OTU0524	Bacteria (100)	Proteobacteria (100)	Gammaproteobacteria (100)	Pseudomonadales (99)	Pseudomonadaceae (99)	Unclassified (<80)
OTU0525	Bacteria (100)	Proteobacteria (100)	Gammaproteobacteria (100)	Enterobacteriales (100)	Enterobacteriaceae (100)	*Proteus (83)*
OTU0526	Bacteria (100)	Firmicutes (100)	Bacilli (100)	Bacillales (100)	Unclassified (<80)	Unclassified (<80)
OTU0527	Bacteria (100)	Firmicutes (100)	Bacilli (100)	Bacillales (100)	Planococcaceae (99)	Unclassified (<80)
OTU0528	Bacteria (100)	Proteobacteria (100)	Gammaproteobacteria (100)	Pseudomonadales (100)	Moraxellaceae (100)	*Acinetobacter (100)*
OTU0529	Bacteria (100)	Firmicutes (100)	Bacilli (100)	Bacillales (100)	Planococcaceae (99)	Unclassified (<80)
OTU0530	Bacteria (100)	Actinobacteria (100)	Actinobacteria (100)	Micrococcales (100)	Micrococcaceae (100)	*Micrococcus (99)*
OTU0531	Bacteria (100)	Proteobacteria (100)	Betaproteobacteria (100)	Burkholderiales (100)	Burkholderiaceae (100)	*Burkholderia (100)*
OTU0532	Bacteria (100)	Proteobacteria (100)	Gammaproteobacteria (100)	Enterobacteriales (100)	Enterobacteriaceae (100)	*Escherichia/Shigella (90)*
OTU0533	Bacteria (100)	Actinobacteria (100)	Actinobacteria (100)	Micrococcales (100)	Micrococcaceae (96)	*Kocuria (89)*
OTU0534	Bacteria (100)	Firmicutes (100)	Bacilli (100)	Lactobacillales (100)	Enterococcaceae (95)	*Vagococcus (95)*
OTU0535	Bacteria (100)	Proteobacteria (100)	Betaproteobacteria (100)	Unclassified (<80)	Unclassified (<80)	Unclassified (<80)
OTU0536	Bacteria (100)	Proteobacteria (100)	Gammaproteobacteria (100)	Xanthomonadales (100)	Xanthomonadaceae (100)	*Stenotrophomonas (100)*
OTU0537	Bacteria (100)	Firmicutes (100)	Bacilli (100)	Bacillales (100)	Planococcaceae (100)	*Planococcaceae ince (96)*
OTU0538	Bacteria (100)	Proteobacteria (100)	Gammaproteobacteria (100)	Aeromonadales (100)	Aeromonadaceae (100)	*Oceanisphaera (96)*
OTU0539	Bacteria (100)	Firmicutes (99)	Bacilli (97)	Bacillales (87)	Planococcaceae (81)	Unclassified (<80)
OTU0540	Bacteria (100)	Proteobacteria (100)	Gammaproteobacteria (100)	Pseudomonadales (100)	Moraxellaceae (100)	*Acinetobacter (100)*
OTU0541	Bacteria (100)	Proteobacteria (100)	Alphaproteobacteria (100)	Rhizobiales (100)	Bradyrhizobiaceae (100)	*Bradyrhizobium (100)*
OTU0542	Bacteria (100)	Firmicutes (100)	Bacilli (100)	Bacillales (99)	Bacillaceae 1 (95)	*Bacillus (92)*
OTU0543	Bacteria (100)	Firmicutes (100)	Bacilli (100)	Bacillales (99)	Unclassified (<80)	Unclassified (<80)
OTU0544	Bacteria (100)	Proteobacteria (100)	Betaproteobacteria (100)	Methylophilales (100)	Methylophilaceae (100)	*Methylophilus (100)*
OTU0545	Bacteria (100)	Firmicutes (100)	Bacilli (100)	Bacillales (99)	Planococcaceae (89)	*Lysinibacillus (81)*
OTU0546	Bacteria (100)	Firmicutes (100)	Clostridia (100)	Clostridiales (100)	Peptostreptococcaceae (100)	*Tepidibacter (100)*
OTU0547	Bacteria (100)	Proteobacteria (100)	Alphaproteobacteria (100)	Rhizobiales (100)	Unclassified (<80)	Unclassified (<80)
OTU0548	Bacteria (100)	Firmicutes (100)	Bacilli (100)	Lactobacillales (91)	Unclassified (<80)	Unclassified (<80)
[illegible]	Bacteria [illegible]	Proteobacteria [illegible]	Gammaproteobacteria [illegible]	Pseudomonadales [illegible]	Moraxellaceae [illegible]	*Acinetobacter* [illegible]

#OTU	DOMAIN (%)	PHYLUM (%)	CLASS/SUBCLASS (%)	ORDER (%)	FAMILY (%)	*GENUS (%)*
**OTU0562**	**Bacteria (100)**	**Proteobacteria (100)**	**Gammaproteobacteria (100)**	**Pseudomonadales (100)**	**Moraxellaceae (100)**	***Acinetobacter (99)***
**OTU0563**	**Bacteria (100)**	**Firmicutes (100)**	**Bacilli (100)**	**Bacillales (100)**	**Unclassified (<80)**	**Unclassified (<80)**
**OTU0564**	**Bacteria (100)**	**Firmicutes (98)**	**Bacilli (97)**	**Bacillales (91)**	**Bacillaceae 2 (86)**	**Unclassified (<80)**
**OTU0565**	**Bacteria (100)**	**Proteobacteria (100)**	**Alphaproteobacteria (100)**	**Sphingomonadales (100)**	**Sphingomonadaceae (100)**	***Sphingobium (89)***
**OTU0566**	**Bacteria (100)**	**Proteobacteria (100)**	**Gammaproteobacteria (100)**	**Pseudomonadales (93)**	**Moraxellaceae (93)**	**Unclassified (<80)**
**OTU0567**	**Bacteria (100)**	**Actinobacteria (100)**	**Actinobacteria (100)**	**Propionibacteriales (100)**	**Propionibacteriaceae (100)**	***Propionibacterium (100)***
**OTU0568**	**Bacteria (100)**	**Actinobacteria (100)**	**Actinobacteria (100)**	**Micrococcales (100)**	**Micrococcaceae (100)**	***Kocuria (91)***
**OTU0569**	**Bacteria (100)**	**Proteobacteria (100)**	**Alphaproteobacteria (100)**	**Sphingomonadales (99)**	**Sphingomonadaceae (99)**	**Unclassified (<80)**
**OTU0570**	**Bacteria (100)**	**Proteobacteria (100)**	**Gammaproteobacteria (100)**	**Chromatiales (98)**	**Chromatiaceae (98)**	***Rheinheimera (98)***
**OTU0571**	**Bacteria (100)**	**Proteobacteria (100)**	**Gammaproteobacteria (100)**	**Pseudomonadales (96)**	**Pseudomonadaceae (96)**	**Unclassified (<80)**
**OTU0572**	**Bacteria (100)**	**Firmicutes (100)**	**Bacilli (100)**	**Bacillales (100)**	**Unclassified (<80)**	**Unclassified (<80)**
**OTU0573**	**Bacteria (100)**	**Firmicutes (100)**	**Bacilli (100)**	**Lactobacillales (100)**	**Enterococcaceae (100)**	***Enterococcus (100)***
**OTU0574**	**Bacteria (100)**	**Firmicutes (100)**	**Bacilli (100)**	**Bacillales (99)**	**Unclassified (<80)**	**Unclassified (<80)**
**OTU0575**	**Bacteria (100)**	**Firmicutes (100)**	**Bacilli (100)**	**Bacillales (100)**	**Unclassified (<80)**	**Unclassified (<80)**
**OTU0576**	**Bacteria (100)**	**Firmicutes (99)**	**Bacilli (99)**	**Lactobacillales (99)**	**Aerococcaceae (98)**	***Aerococcus (97)***
**OTU0577**	**Bacteria (100)**	**Proteobacteria (98)**	**Alphaproteobacteria (98)**	**Caulobacterales (81)**	**Caulobacteraceae (81)**	***Brevundimonas (81)***
**OTU0578**	**Bacteria (100)**	**Actinobacteria (100)**	**Actinobacteria (100)**	**Jiangellales (100)**	**Jiangellaceae (95)**	***Jiangella (91)***
**OTU0579**	**Bacteria (100)**	**Actinobacteria (100)**	**Actinobacteria (100)**	**Micrococcales (100)**	**Micrococcaceae (100)**	**Unclassified (<80)**
**OTU0580**	**Bacteria (100)**	**Proteobacteria (100)**	**Gammaproteobacteria (100)**	**Aeromonadales (100)**	**Aeromonadaceae (100)**	***Oceanisphaera (100)***
**OTU0581**	**Bacteria (100)**	**Firmicutes (100)**	**Bacilli (100)**	**Lactobacillales (100)**	**Enterococcaceae (82)**	**Unclassified (<80)**
**OTU0582**	**Bacteria (100)**	**Proteobacteria (100)**	**Gammaproteobacteria (100)**	**Oceanospirillales (100)**	**Halomonadaceae (100)**	***Halomonas (100)***
**OTU0583**	**Bacteria (100)**	**Actinobacteria (100)**	**Actinobacteria (100)**	**Pseudonocardiales (100)**	**Pseudonocardiaceae (100)**	***Pseudonocardia (87)***
**OTU0584**	**Bacteria (100)**	**Actinobacteria (100)**	**Actinobacteria (100)**	**Micrococcales (100)**	**Microbacteriaceae (98)**	**Unclassified (<80)**
**OTU0585**	**Bacteria (100)**	**Proteobacteria (100)**	**Gammaproteobacteria (100)**	**Xanthomonadales (100)**	**Xanthomonadaceae (100)**	***Stenotrophomonas (97)***
**OTU0586**	**Bacteria (100)**	**Proteobacteria (100)**	**Alphaproteobacteria (100)**	**Rhodospirillales (100)**	**Rhodospirillaceae (100)**	***Thalassospira (100)***
**OTU0587**	**Bacteria (100)**	**Acidobacteria (100)**	**Acidobacteria_Gp4 (100)**	**--------†**	**--------†**	***Blastocatella (81)***
**OTU0588**	**Bacteria (100)**	**Firmicutes (100)**	**Bacilli (100)**	**Bacillales (100)**	**Paenibacillaceae 1 (100)**	***Paenibacillus (100)***
**OTU0589**	**Bacteria (100)**	**Actinobacteria (100)**	**Actinobacteria (100)**	**Micrococcales (100)**	**Micrococcaceae (100)**	***Arthrobacter (90)***
**OTU0590**	**Bacteria (100)**	**Actinobacteria (100)**	**Actinobacteria (100)**	**Micrococcales (100)**	**Dermatophilaceae (82)**	***Piscicoccus (80)***
**OTU0591**	**Bacteria (100)**	**Actinobacteria (100)**	**Actinobacteria (100)**	**Pseudonocardiales (100)**	**Pseudonocardiaceae (100)**	***Saccharopolyspora (81)***
**OTU0592**	**Bacteria (100)**	**Firmicutes (100)**	**Bacilli (100)**	**Bacillales (100)**	**Bacillaceae 2 (100)**	***Virgibacillus (82)***
**OTU0593**	**Bacteria (100)**	**Proteobacteria (100)**	**Gammaproteobacteria (100)**	**Oceanospirillales (100)**	**Alcanivoracaceae (100)**	***Alcanivorax (100)***
**OTU0594**	**Bacteria (100)**	**Firmicutes (100)**	**Bacilli (100)**	**Bacillales (100)**	**Paenibacillaceae 1 (93)**	**Unclassified (<80)**

#OTU	DOMAIN (%)	PHYLUM (%)	CLASS/SUBCLASS (%)	ORDER (%)	FAMILY (%)	*GENUS (%)*
OTU0609	Bacteria (100)	Proteobacteria (100)	Gammaproteobacteria (100)	Xanthomonadales (100)	Xanthomonadaceae (100)	*Stenotrophomonas (100)*
OTU0610	Bacteria (100)	Firmicutes (100)	Bacilli (100)	Lactobacillales (100)	Leuconostocaceae (100)	*Weissella (100)*
OTU0611	Bacteria (100)	Proteobacteria (100)	Betaproteobacteria (100)	Burkholderiales (99)	Burkholderiaceae (99)	*Burkholderia (99)*
OTU0612	Bacteria (100)	Actinobacteria (100)	Actinobacteria (100)	Pseudonocardiales (100)	Pseudonocardiaceae (100)	Unclassified (<80)
OTU0613	Bacteria (100)	Actinobacteria (100)	Actinobacteria (100)	Corynebacteriales (100)	Nocardiaceae (98)	*Gordonia (97)*
OTU0614	Bacteria (100)	Proteobacteria (100)	Alphaproteobacteria (100)	Rhodobacterales (100)	Rhodobacteraceae (100)	Unclassified (<80)
OTU0615	Bacteria (100)	Proteobacteria (100)	Gammaproteobacteria (100)	Enterobacteriales (100)	Enterobacteriaceae (100)	Unclassified (<80)
OTU0616	Bacteria (100)	Actinobacteria (100)	Actinobacteria (100)	Micrococcales (100)	Micrococcaceae (99)	*Kocuria (85)*
OTU0617	Bacteria (100)	Proteobacteria (100)	Alphaproteobacteria (100)	Rhizobiales (100)	Methylobacteriaceae (99)	*Methylobacterium (99)*
OTU0618	Bacteria (100)	Proteobacteria (100)	Alphaproteobacteria (100)	Sphingomonadales (97)	Sphingomonadaceae (97)	*Sphingomonas (88)*
OTU0619	Bacteria (100)	Actinobacteria (100)	Actinobacteria (100)	Bifidobacteriales (100)	Bifidobacteriaceae (100)	*Bifidobacterium (100)*
OTU0620	Bacteria (100)	Actinobacteria (100)	Actinobacteria (100)	Pseudonocardiales (100)	Pseudonocardiaceae (96)	Unclassified (<80)
OTU0621	Bacteria (100)	Firmicutes (100)	Bacilli (100)	Lactobacillales (87)	Enterococcaceae (80)	Unclassified (<80)
OTU0622	Bacteria (100)	Bacteroidetes (100)	Cytophagia (94)	Cytophagales (94)	Unclassified (<80)	Unclassified (<80)
OTU0623	Bacteria (100)	Proteobacteria (100)	Gammaproteobacteria (100)	Enterobacteriales (100)	Enterobacteriaceae (100)	Unclassified (<80)
OTU0624	Bacteria (100)	Proteobacteria (100)	Gammaproteobacteria (100)	Enterobacteriales (100)	Enterobacteriaceae (100)	Unclassified (<80)
OTU0625	Bacteria (100)	Proteobacteria (100)	Gammaproteobacteria (100)	Enterobacteriales (100)	Enterobacteriaceae (100)	Unclassified (<80)
OTU0626	Bacteria (100)	Firmicutes (100)	Bacilli (100)	Unclassified (<80)	Unclassified (<80)	Unclassified (<80)
OTU0627	Bacteria (100)	Firmicutes (100)	Bacilli (100)	Bacillales (100)	Bacillaceae 1 (98)	*Anoxybacillus (84)*
OTU0628	Bacteria (100)	Firmicutes (100)	Bacilli (100)	Bacillales (99)	Planococcaceae (93)	Unclassified (<80)
OTU0629	Bacteria (100)	Firmicutes (100)	Bacilli (100)	Bacillales (100)	Bacillaceae 2 (95)	Unclassified (<80)
OTU0630	Bacteria (100)	Firmicutes (100)	Bacilli (100)	Bacillales (100)	Planococcaceae (95)	Unclassified (<80)
OTU0631	Bacteria (100)	Firmicutes (100)	Bacilli (100)	Bacillales (95)	Unclassified (<80)	Unclassified (<80)
OTU0632	Bacteria (100)	Firmicutes (100)	Bacilli (100)	Bacillales (100)	Bacillaceae 2 (100)	*Oceanobacillus (100)*
OTU0633	Bacteria (100)	Actinobacteria (100)	Actinobacteria (100)	Pseudonocardiales (100)	Pseudonocardiaceae (100)	*Actinomycetospora (100)*
OTU0634	Bacteria (100)	Proteobacteria (100)	Gammaproteobacteria (100)	Pseudomonadales (99)	Moraxellaceae (99)	*Acinetobacter (91)*
OTU0635	Bacteria (100)	Actinobacteria (100)	Actinobacteria (100)	Micrococcales (100)	Micrococcaceae (100)	*Kocuria (98)*
OTU0636	Bacteria (100)	Firmicutes (100)	Bacilli (100)	Bacillales (100)	Unclassified (<80)	Unclassified (<80)
OTU0637	Bacteria (100)	Firmicutes (100)	Bacilli (100)	Lactobacillales (91)	Unclassified (<80)	Unclassified (<80)
OTU0638	Bacteria (100)	Actinobacteria (100)	Actinobacteria (100)	Pseudonocardiales (100)	Pseudonocardiaceae (100)	*Saccharopolyspora (91)*
OTU0639	Bacteria (100)	Firmicutes (100)	Clostridia (100)	Clostridiales (100)	Clostridiaceae 1 (100)	*Clostridium sensu st (86)*
OTU0640	Bacteria (100)	Actinobacteria (100)	Actinobacteria (100)	Pseudonocardiales (100)	Pseudonocardiaceae (100)	Unclassified (<80)
OTU0641	Bacteria (100)	Actinobacteria (100)	Actinobacteria (100)	Micrococcales (100)	Dermabacteraceae (100)	*Brachybacterium (100)*

#OTU	DOMAIN (%)	PHYLUM (%)	CLASS/SUBCLASS (%)	ORDER (%)	FAMILY (%)	*GENUS (%)*
**OTU0656**	**Bacteria (100)**	**Actinobacteria (100)**	**Actinobacteria (100)**	**Micrococcales (100)**	**Micrococcaceae (100)**	***Kocuria (93)***
**OTU0657**	**Bacteria (100)**	**Actinobacteria (100)**	**Actinobacteria (100)**	**Pseudonocardiales (100)**	**Pseudonocardiaceae (97)**	**Unclassified (<80)**
**OTU0658**	**Bacteria (100)**	**Actinobacteria (100)**	**Actinobacteria (100)**	**Pseudonocardiales (100)**	**Pseudonocardiaceae (100)**	***Pseudonocardia (96)***
**OTU0659**	**Bacteria (100)**	**Proteobacteria (100)**	**Alphaproteobacteria (100)**	**Sphingomonadales (100)**	**Sphingomonadaceae (99)**	**Unclassified (<80)**
**OTU0660**	**Bacteria (100)**	**Proteobacteria (100)**	**Alphaproteobacteria (100)**	**Rhizobiales (100)**	**Unclassified (<80)**	**Unclassified (<80)**
**OTU0661**	**Bacteria (100)**	**Firmicutes (100)**	**Bacilli (92)**	**Unclassified (<80)**	**Unclassified (<80)**	**Unclassified (<80)**
**OTU0662**	**Bacteria (100)**	**Firmicutes (100)**	**Negativicutes (100)**	**Selenomonadales (100)**	**Veillonellaceae (100)**	***Dialister (100)***
**OTU0663**	**Bacteria (100)**	**Proteobacteria (100)**	**Gammaproteobacteria (100)**	**Alteromonadales (96)**	**Shewanellaceae (94)**	***Shewanella (94)***
**OTU0664**	**Bacteria (100)**	**Proteobacteria (100)**	**Gammaproteobacteria (100)**	**Aeromonadales (100)**	**Aeromonadaceae (100)**	***Oceanimonas (100)***
**OTU0665**	**Bacteria (100)**	**Bacteroidetes (100)**	**Bacteroidia (100)**	**Bacteroidales (100)**	**Prevotellaceae (100)**	***Prevotella (100)***
**OTU0666**	**Bacteria (100)**	**Bacteroidetes (100)**	**Unclassified (<80)**	**Unclassified (<80)**	**Unclassified (<80)**	**Unclassified (<80)**
**OTU0667**	**Bacteria (100)**	**Proteobacteria (100)**	**Alphaproteobacteria (100)**	**Rhodobacterales (100)**	**Rhodobacteraceae (100)**	**Unclassified (<80)**
**OTU0668**	**Bacteria (100)**	**Firmicutes (100)**	**Bacilli (100)**	**Lactobacillales (99)**	**Enterococcaceae (99)**	***Enterococcus (98)***
**OTU0669**	**Bacteria (100)**	**Proteobacteria (100)**	**Gammaproteobacteria (100)**	**Pseudomonadales (99)**	**Pseudomonadaceae (99)**	***Pseudomonas (98)***
**OTU0670**	**Bacteria (100)**	**Proteobacteria (100)**	**Gammaproteobacteria (100)**	**Pseudomonadales (100)**	**Pseudomonadaceae (100)**	***Pseudomonas (93)***
**OTU0671**	**Bacteria (100)**	**Firmicutes (100)**	**Bacilli (100)**	**Bacillales (100)**	**Bacillaceae 2 (100)**	***Virgibacillus (97)***
**OTU0672**	**Bacteria (100)**	**Firmicutes (100)**	**Bacilli (99)**	**Bacillales (98)**	**Paenibacillaceae 1 (98)**	***Brevibacillus (96)***
**OTU0673**	**Bacteria (100)**	**Actinobacteria (100)**	**Actinobacteria (100)**	**Micrococcales (100)**	**Micrococcaceae (100)**	**Unclassified (<80)**
**OTU0674**	**Bacteria (100)**	**Actinobacteria (100)**	**Actinobacteria (100)**	**Propionibacteriales (100)**	**Nocardiopsaceae (100)**	**Unclassified (<80)**
**OTU0675**	**Bacteria (100)**	**Actinobacteria (100)**	**Actinobacteria (100)**	**Micrococcales (100)**	**Brevibacteriaceae (100)**	***Brevibacterium (100)***
**OTU0676**	**Bacteria (100)**	**Proteobacteria (100)**	**Gammaproteobacteria (100)**	**Pseudomonadales (93)**	**Pseudomonadaceae (93)**	**Unclassified (<80)**
**OTU0677**	**Bacteria (100)**	**Firmicutes (100)**	**Bacilli (100)**	**Bacillales (100)**	**Planococcaceae (99)**	***Sporosarcina (99)***
**OTU0678**	**Bacteria (100)**	**Proteobacteria (100)**	**Gammaproteobacteria (99)**	**Enterobacteriales (98)**	**Enterobacteriaceae (98)**	**Unclassified (<80)**
**OTU0679**	**Bacteria (100)**	**Actinobacteria (100)**	**Actinobacteria (100)**	**Actinomycetales (100)**	**Actinomycetaceae (100)**	***Actinomyces (100)***
**OTU0680**	**Bacteria (100)**	**Proteobacteria (100)**	**Gammaproteobacteria (100)**	**Pseudomonadales (98)**	**Pseudomonadaceae (98)**	**Unclassified (<80)**
**OTU0681**	**Bacteria (100)**	**Proteobacteria (100)**	**Alphaproteobacteria (100)**	**Rhizobiales (99)**	**Rhizobiaceae (92)**	**Unclassified (<80)**
**OTU0682**	**Bacteria (100)**	**Firmicutes (100)**	**Bacilli (100)**	**Bacillales (96)**	**Staphylococcaceae (94)**	***Staphylococcus (88)***
**OTU0683**	**Bacteria (100)**	**Proteobacteria (100)**	**Betaproteobacteria (100)**	**Burkholderiales (95)**	**Burkholderiaceae (95)**	***Burkholderia (92)***
**OTU0684**	**Bacteria (100)**	**Proteobacteria (100)**	**Gammaproteobacteria (100)**	**Enterobacteriales (100)**	**Enterobacteriaceae (100)**	**Unclassified (<80)**
**OTU0685**	**Bacteria (100)**	**Firmicutes (100)**	**Bacilli (100)**	**Bacillales (98)**	**Bacillaceae 1 (98)**	**Unclassified (<80)**
**OTU0686**	**Bacteria (100)**	**Actinobacteria (100)**	**Actinobacteria (100)**	**Pseudonocardiales (100)**	**Pseudonocardiaceae (100)**	**Unclassified (<80)**
**OTU0687**	**Bacteria (100)**	**Firmicutes (100)**	**Bacilli (100)**	**Bacillales (90)**	**Unclassified (<80)**	**Unclassified (<80)**
**OTU0688**	**Bacteria (100)**	**Firmicutes (100)**	**Bacilli (100)**	**Bacillales (100)**	**Planococcaceae (99)**	***Lysinibacillus (94)***

#OTU	DOMAIN (%)	PHYLUM (%)	CLASS/SUBCLASS (%)	ORDER (%)	FAMILY (%)	*GENUS (%)*
**OTU0703**	**Bacteria (100)**	**Proteobacteria (100)**	**Gammaproteobacteria (100)**	**Alteromonadales (100)**	**Alteromonadaceae (100)**	***Alteromonas (90)***
**OTU0704**	**Bacteria (100)**	**Actinobacteria (100)**	**Actinobacteria (100)**	**Pseudonocardiales (100)**	**Pseudonocardiaceae (100)**	***Saccharopolyspora (92)***
**OTU0705**	**Bacteria (100)**	**Firmicutes (100)**	**Bacilli (93)**	**Bacillales (90)**	**Unclassified (<80)**	**Unclassified (<80)**
**OTU0706**	**Bacteria (100)**	**Actinobacteria (100)**	**Actinobacteria (100)**	**Micrococcales (100)**	**Micrococcaceae (100)**	***Arthrobacter (85)***
**OTU0707**	**Bacteria (100)**	**Proteobacteria (100)**	**Gammaproteobacteria (100)**	**Aeromonadales (100)**	**Aeromonadaceae (100)**	***Oceanisphaera (100)***
**OTU0708**	**Bacteria (100)**	**Proteobacteria (100)**	**Alphaproteobacteria (100)**	**Rhizobiales (100)**	**Hyphomicrobiaceae (86)**	***Devosia (86)***
**OTU0709**	**Bacteria (100)**	**Unclassified (<80)**	**Unclassified (<80)**	**Unclassified (<80)**	**Unclassified (<80)**	**Unclassified (<80)**
**OTU0710**	**Bacteria (100)**	**Actinobacteria (100)**	**Actinobacteria (100)**	**Pseudonocardiales (100)**	**Pseudonocardiaceae (100)**	***Pseudonocardia (85)***
**OTU0711**	**Bacteria (100)**	**Proteobacteria (100)**	**Gammaproteobacteria (100)**	**Pseudomonadales (100)**	**Moraxellaceae (100)**	***Acinetobacter (100)***
**OTU0712**	**Bacteria (100)**	**Proteobacteria (100)**	**Gammaproteobacteria (100)**	**Pseudomonadales (100)**	**Pseudomonadaceae (100)**	***Pseudomonas (98)***
**OTU0713**	**Bacteria (100)**	**Bacteroidetes (100)**	**Flavobacteriia (100)**	**Flavobacteriales (100)**	**Flavobacteriaceae (100)**	***Chryseobacterium (100)***
**OTU0714**	**Bacteria (100)**	**Firmicutes (100)**	**Bacilli (99)**	**Bacillales (98)**	**Unclassified (<80)**	**Unclassified (<80)**
**OTU0715**	**Bacteria (100)**	**Cyanobac./Chloroplast (100)**	**--------**	**--------**	**--------**	**--------**
**OTU0716**	**Bacteria (100)**	**Proteobacteria (100)**	**Alphaproteobacteria (100)**	**Sphingomonadales (100)**	**Sphingomonadaceae (100)**	***Sphingomonas (100)***
**OTU0717**	**Bacteria (100)**	**Firmicutes (100)**	**Bacilli (100)**	**Bacillales (99)**	**Unclassified (<80)**	**Unclassified (<80)**
**OTU0718**	**Bacteria (100)**	**Proteobacteria (100)**	**Gammaproteobacteria (100)**	**Pseudomonadales (99)**	**Pseudomonadaceae (99)**	**Unclassified (<80)**
**OTU0719**	**Bacteria (100)**	**Firmicutes (100)**	**Bacilli (94)**	**Bacillales (92)**	**Unclassified (<80)**	**Unclassified (<80)**
**OTU0720**	**Bacteria (100)**	**Proteobacteria (100)**	**Alphaproteobacteria (100)**	**Sphingomonadales (100)**	**Unclassified (<80)**	**Unclassified (<80)**
**OTU0721**	**Bacteria (100)**	**Proteobacteria (100)**	**Gammaproteobacteria (100)**	**Enterobacteriales (100)**	**Enterobacteriaceae (100)**	**Unclassified (<80)**
**OTU0722**	**Bacteria (100)**	**Proteobacteria (100)**	**Gammaproteobacteria (100)**	**Enterobacteriales (100)**	**Enterobacteriaceae (100)**	**Unclassified (<80)**
**OTU0723**	**Bacteria (100)**	**Proteobacteria (100)**	**Gammaproteobacteria (100)**	**Alteromonadales (98)**	**Alteromonadaceae (98)**	***Alishewanella (98)***
**OTU0724**	**Bacteria (100)**	**Proteobacteria (100)**	**Alphaproteobacteria (100)**	**Sphingomonadales (95)**	**Sphingomonadaceae (95)**	**Unclassified (<80)**
**OTU0725**	**Bacteria (100)**	**Proteobacteria (100)**	**Gammaproteobacteria (100)**	**Vibrionales (100)**	**Vibrionaceae (100)**	***Vibrio (100)***
**OTU0726**	**Bacteria (100)**	**Firmicutes (100)**	**Bacilli (100)**	**Bacillales (98)**	**Bacillaceae 1 (98)**	***Bacillus (81)***
**OTU0727**	**Bacteria (100)**	**Proteobacteria (100)**	**Alphaproteobacteria (100)**	**Sphingomonadales (100)**	**Sphingomonadaceae (100)**	***Sphingomonas (97)***
**OTU0728**	**Bacteria (100)**	**Proteobacteria (100)**	**Gammaproteobacteria (100)**	**Oceanospirillales (100)**	**Halomonadaceae (100)**	**Unclassified (<80)**
**OTU0729**	**Bacteria (100)**	**Firmicutes (100)**	**Bacilli (100)**	**Bacillales (100)**	**Unclassified (<80)**	**Unclassified (<80)**
**OTU0730**	**Bacteria (100)**	**Proteobacteria (100)**	**Gammaproteobacteria (100)**	**Pseudomonadales (100)**	**Moraxellaceae (100)**	***Enhydrobacter (100)***
**OTU0731**	**Bacteria (100)**	**Proteobacteria (100)**	**Alphaproteobacteria (100)**	**Rhizobiales (100)**	**Methylobacteriaceae (100)**	***Methylobacterium (96)***
**OTU0732**	**Bacteria (100)**	**Firmicutes (100)**	**Bacilli (100)**	**Bacillales (100)**	**Unclassified (<80)**	**Unclassified (<80)**
**OTU0733**	**Bacteria (100)**	**Actinobacteria (100)**	**Actinobacteria (100)**	**Pseudonocardiales (100)**	**Pseudonocardiaceae (100)**	***Pseudonocardia (99)***
**OTU0734**	**Bacteria (100)**	**Proteobacteria (100)**	**Gammaproteobacteria (100)**	**Alteromonadales (96)**	**Shewanellaceae (95)**	***Shewanella (95)***
**OTU0735**	**Bacteria (100)**	**Proteobacteria (100)**	**Betaproteobacteria (100)**	**Unclassified (<80)**	**Unclassified (<80)**	**Unclassified (<80)**

#OTU	DOMAIN (%)	PHYLUM (%)	CLASS/SUBCLASS (%)	ORDER (%)	FAMILY (%)	*GENUS (%)*
OTU0745	Bacteria (100)	Proteobacteria (100)	Betaproteobacteria (100)	Burkholderiales (89)	Oxalobacteraceae (89)	Unclassified (<80)
OTU0746	Bacteria (100)	Proteobacteria (100)	Betaproteobacteria (100)	Burkholderiales (100)	Burkholderiaceae (100)	*Burkholderia (98)*
OTU0747	Bacteria (100)	Proteobacteria (100)	Alphaproteobacteria (100)	Rhodospirillales (100)	Acetobacteraceae (100)	*Roseomonas (100)*
OTU0748	Bacteria (100)	Actinobacteria (100)	Actinobacteria (100)	Micrococcales (100)	Micrococcaceae (86)	Unclassified (<80)
OTU0749	Bacteria (100)	Firmicutes (100)	Bacilli (100)	Bacillales (100)	Unclassified (<80)	Unclassified (<80)
OTU0750	Bacteria (100)	Proteobacteria (100)	Gammaproteobacteria (100)	Pseudomonadales (99)	Moraxellaceae (99)	Unclassified (<80)
OTU0751	Bacteria (100)	Proteobacteria (100)	Betaproteobacteria (100)	Neisseriales (100)	Neisseriaceae (100)	Unclassified (<80)
OTU0752	Bacteria (100)	Firmicutes (99)	Bacilli (99)	Bacillales (88)	Staphylococcaceae (82)	Unclassified (<80)
OTU0753	Bacteria (100)	Proteobacteria (100)	Alphaproteobacteria (100)	Rhizobiales (98)	Methylobacteriaceae (98)	*Microvirga (96)*
OTU0754	Bacteria (100)	Actinobacteria (100)	Actinobacteria (100)	Micrococcales (100)	Brevibacteriaceae (95)	Unclassified (<80)
OTU0755	Bacteria (100)	Proteobacteria (100)	Gammaproteobacteria (100)	Enterobacteriales (98)	Enterobacteriaceae (98)	Unclassified (<80)
OTU0756	Bacteria (100)	Proteobacteria (100)	Gammaproteobacteria (100)	Pseudomonadales (100)	Moraxellaceae (100)	*Acinetobacter (100)*
OTU0757	Bacteria (100)	Actinobacteria (100)	Actinobacteria (100)	Micrococcales (100)	Micrococcaceae (100)	*Micrococcus (96)*
OTU0758	Bacteria (100)	Firmicutes (100)	Bacilli (97)	Bacillales (91)	Planococcaceae (82)	Unclassified (<80)
OTU0759	Bacteria (100)	Proteobacteria (100)	Alphaproteobacteria (99)	Rhizobiales (97)	Methylobacteriaceae (80)	Unclassified (<80)
OTU0760	Bacteria (100)	Firmicutes (100)	Bacilli (100)	Lactobacillales (99)	Aerococcaceae (98)	*Aerococcus (93)*
OTU0761	Bacteria (100)	Firmicutes (100)	Bacilli (100)	Bacillales (100)	Bacillaceae 1 (99)	Unclassified (<80)
OTU0762	Bacteria (100)	Actinobacteria (100)	Actinobacteria (100)	Micrococcales (100)	Intrasporangiaceae (100)	Unclassified (<80)
OTU0763	Bacteria (100)	Proteobacteria (100)	Alphaproteobacteria (100)	Rhizobiales (100)	Phyllobacteriaceae (99)	*Chelativorans (97)*
OTU0764	Bacteria (100)	Firmicutes (100)	Bacilli (100)	Lactobacillales (100)	Carnobacteriaceae (94)	*Granulicatella (88)*
OTU0765	Bacteria (100)	Proteobacteria (100)	Gammaproteobacteria (100)	Pasteurellales (100)	Pasteurellaceae (100)	Unclassified (<80)
OTU0766	Bacteria (100)	Proteobacteria (100)	Gammaproteobacteria (100)	Pseudomonadales (98)	Moraxellaceae (98)	Unclassified (<80)
OTU0767	Bacteria (100)	Firmicutes (100)	Bacilli (100)	Bacillales (100)	Staphylococcaceae (99)	*Staphylococcus (98)*
OTU0768	Bacteria (100)	Proteobacteria (100)	Betaproteobacteria (99)	Neisseriales (91)	Neisseriaceae (91)	Unclassified (<80)
OTU0769	Bacteria (100)	Proteobacteria (100)	Gammaproteobacteria (100)	Enterobacteriales (100)	Enterobacteriaceae (100)	Unclassified (<80)
OTU0770	Bacteria (100)	Proteobacteria (100)	Alphaproteobacteria (100)	Sphingomonadales (98)	Sphingomonadaceae (98)	Unclassified (<80)
OTU0771	Bacteria (100)	Firmicutes (100)	Bacilli (100)	Bacillales (100)	Planococcaceae (84)	Unclassified (<80)
OTU0772	Bacteria (100)	Actinobacteria (91)	Actinobacteria (91)	Corynebacteriales (91)	Unclassified (<80)	Unclassified (<80)
OTU0773	Bacteria (100)	Firmicutes (100)	Bacilli (100)	Bacillales (100)	Staphylococcaceae (100)	*Staphylococcus (100)*
OTU0774	Bacteria (100)	Actinobacteria (100)	Actinobacteria (100)	Pseudonocardiales (100)	Pseudonocardiaceae (100)	Unclassified (<80)
OTU0775	Bacteria (100)	Proteobacteria (100)	Gammaproteobacteria (100)	Xanthomonadales (100)	Xanthomonadaceae (100)	*Vulcaniibacterium (100)*
OTU0776	Bacteria (100)	Actinobacteria (100)	Actinobacteria (100)	Propionibacteriales (100)	Propionibacteriaceae (100)	*Propionibacterium (100)*
OTU0777	Bacteria (100)	Proteobacteria (100)	Alphaproteobacteria (100)	Sphingomonadales (97)	Sphingomonadaceae (95)	*Sphingomonas (90)*
OTU0778	Bacteria (100)	Actinobacteria (100)	Actinobacteria (100)	Micrococcales (100)	Dermacoccaceae (86)	*Kytococcus (84)*
[illegible]	Bacteria	Proteobacteria	Alphaproteobacteria	Rhodospirillales	Acetobacteraceae	Unclassified

#OTU	DOMAIN (%)	PHYLUM (%)	CLASS/SUBCLASS (%)	ORDER (%)	FAMILY (%)	*GENUS (%)*
OTU0791	Bacteria (100)	Actinobacteria (100)	Actinobacteria (100)	Propionibacteriales (100)	Propionibacteriaceae (99)	*Propionibacterium (86)*
OTU0792	Bacteria (100)	Proteobacteria (100)	Gammaproteobacteria (100)	Enterobacteriales (99)	Enterobacteriaceae (99)	Unclassified (<80)
OTU0793	Bacteria (100)	Actinobacteria (100)	Actinobacteria (100)	Corynebacteriales (100)	Unclassified (<80)	Unclassified (<80)
OTU0794	Bacteria (100)	Proteobacteria (100)	Betaproteobacteria (90)	Burkholderiales (90)	Burkholderiales incertae sedis (85)	*Aquabacterium (81)*
OTU0795	Bacteria (100)	Proteobacteria (100)	Gammaproteobacteria (100)	Chromatiales (100)	Chromatiaceae (100)	*Rheinheimera (100)*
OTU0796	Bacteria (100)	Proteobacteria (100)	Gammaproteobacteria (100)	Xanthomonadales (100)	Xanthomonadaceae (100)	*Lysobacter (88)*
OTU0797	Bacteria (100)	Actinobacteria (100)	Actinobacteria (100)	Corynebacteriales (100)	Corynebacteriaceae (93)	*Corynebacterium (90)*
OTU0798	Bacteria (100)	Actinobacteria (100)	Actinobacteria (100)	Micrococcales (100)	Micrococcaceae (99)	*Kocuria (87)*
OTU0799	Bacteria (100)	Firmicutes (100)	Bacilli (100)	Bacillales (85)	Unclassified (<80)	Unclassified (<80)
OTU0800	Bacteria (100)	Firmicutes (100)	Bacilli (100)	Bacillales (99)	Bacillaceae 2 (95)	Unclassified (<80)
OTU0801	Bacteria (100)	Proteobacteria (100)	Gammaproteobacteria (100)	Pseudomonadales (100)	Moraxellaceae (100)	*Acinetobacter (100)*
OTU0802	Bacteria (100)	Proteobacteria (100)	Gammaproteobacteria (99)	Pseudomonadales (86)	Pseudomonadaceae (86)	Unclassified (<80)
OTU0803	Bacteria (100)	Proteobacteria (100)	Gammaproteobacteria (100)	Pseudomonadales (100)	Pseudomonadaceae (100)	*Pseudomonas (94)*
OTU0804	Bacteria (100)	Proteobacteria (100)	Gammaproteobacteria (100)	Enterobacteriales (100)	Enterobacteriaceae (100)	Unclassified (<80)
OTU0805	Bacteria (100)	Actinobacteria (100)	Actinobacteria (100)	Propionibacteriales (100)	Propionibacteriaceae (100)	*Propionibacterium (100)*
OTU0806	Bacteria (100)	Actinobacteria (100)	Actinobacteria (100)	Corynebacteriales (100)	Corynebacteriaceae (100)	*Corynebacterium (100)*
OTU0807	Bacteria (100)	Actinobacteria (100)	Actinobacteria (100)	Corynebacteriales (100)	Unclassified (<80)	Unclassified (<80)
OTU0808	Bacteria (100)	Actinobacteria (100)	Actinobacteria (100)	Corynebacteriales (98)	Unclassified (<80)	Unclassified (<80)
OTU0809	Bacteria (100)	Proteobacteria (100)	Alphaproteobacteria (100)	Rhodospirillales (100)	Acetobacteraceae (100)	Unclassified (<80)
OTU0810	Bacteria (100)	Firmicutes (100)	Bacilli (100)	Bacillales (100)	Staphylococcaceae (90)	*Staphylococcus (89)*
OTU0811	Bacteria (100)	Firmicutes (100)	Bacilli (99)	Bacillales (99)	Staphylococcaceae (95)	*Staphylococcus (93)*
OTU0812	Bacteria (100)	Actinobacteria (100)	Actinobacteria (100)	Propionibacteriales (100)	Nocardioidaceae (100)	*Nocardioides (100)*
OTU0813	Bacteria (100)	Actinobacteria (100)	Actinobacteria (100)	Pseudonocardiales (100)	Pseudonocardiaceae (100)	*Actinomycetospora (100)*
OTU0814	Bacteria (100)	Proteobacteria (100)	Alphaproteobacteria (100)	Rhizobiales (100)	Hyphomicrobiaceae (97)	*Devosia (97)*
OTU0815	Bacteria (100)	Firmicutes (97)	Bacilli (97)	Bacillales (95)	Staphylococcaceae (89)	*Staphylococcus (81)*
OTU0816	Bacteria (100)	Proteobacteria (100)	Gammaproteobacteria (100)	Pseudomonadales (100)	Moraxellaceae (100)	*Enhydrobacter (100)*
OTU0817	Bacteria (100)	Firmicutes (100)	Bacilli (100)	Lactobacillales (100)	Enterococcaceae (100)	*Enterococcus (100)*
OTU0818	Bacteria (100)	Actinobacteria (100)	Actinobacteria (100)	Corynebacteriales (100)	Corynebacteriaceae (100)	*Corynebacterium (100)*
OTU0819	Bacteria (100)	Firmicutes (100)	Clostridia (100)	Clostridiales (100)	Clostridiaceae 1 (94)	*Unclassified (<80)*
OTU0820	Bacteria (100)	Firmicutes (100)	Bacilli (99)	Bacillales (94)	Staphylococcaceae (87)	*Staphylococcus (86)*
OTU0821	Bacteria (100)	Firmicutes (100)	Bacilli (100)	Lactobacillales (100)	Aerococcaceae (100)	*Aerococcus (100)*
OTU0822	Bacteria (100)	Actinobacteria (100)	Actinobacteria (100)	Propionibacteriales (100)	Propionibacteriaceae (100)	*Propionibacterium (100)*
OTU0823	Bacteria (100)	Firmicutes (100)	Bacilli (100)	Bacillales (100)	Staphylococcaceae (100)	*Staphylococcus (98)*
OTU0824	Bacteria (100)	Firmicutes (99)	Bacilli (96)	Bacillales (81)	Unclassified (<80)	Unclassified (<80)
[illegible]	Bacteria [illegible]	Firmicutes [illegible]	Bacilli [illegible]	Bacillales [illegible]	Staphylococcaceae [illegible]	*Staphylococcus* [illegible]

#OTU	DOMAIN (%)	PHYLUM (%)	CLASS/SUBCLASS (%)	ORDER (%)	FAMILY (%)	*GENUS (%)*
OTU0838	Bacteria (100)	Proteobacteria (100)	Alphaproteobacteria (100)	Sphingomonadales (97)	Sphingomonadaceae (97)	Unclassified (<80)
OTU0839	Bacteria (100)	Firmicutes (100)	Clostridia (100)	Clostridiales (100)	Clostridiales incertae sedis XI (100)	*Finegoldia (100)*
OTU0840	Bacteria (100)	Bacteroidetes (95)	Unclassified (<80)	Unclassified (<80)	Unclassified (<80)	Unclassified (<80)
OTU0841	Bacteria (100)	Firmicutes (100)	Bacilli (100)	Bacillales (99)	Bacillaceae 2 (98)	Unclassified (<80)
OTU0842	Bacteria (100)	Firmicutes (100)	Bacilli (100)	Bacillales (97)	Staphylococcaceae (96)	*Staphylococcus (91)*
OTU0843	Bacteria (100)	Proteobacteria (100)	Betaproteobacteria (100)	Burkholderiales (100)	Oxalobacteraceae (98)	Unclassified (<80)
OTU0844	Bacteria (100)	Firmicutes (100)	Clostridia (100)	Clostridiales (100)	Clostridiaceae 1 (100)	*Hathewaya (100)*
OTU0845	Bacteria (100)	Actinobacteria (100)	Actinobacteria (100)	Pseudonocardiales (100)	Unclassified (<80)	Unclassified (<80)
OTU0846	Bacteria (100)	Proteobacteria (100)	Alphaproteobacteria (100)	Caulobacterales (100)	Caulobacteraceae (100)	*Brevundimonas (100)*
OTU0847	Bacteria (100)	Actinobacteria (100)	Actinobacteria (100)	Micrococcales (100)	Micrococcaceae (100)	*Arthrobacter (87)*
OTU0848	Bacteria (100)	Proteobacteria (100)	Alphaproteobacteria (100)	Sphingomonadales (100)	Sphingomonadaceae (100)	*Sphingomonas (100)*
OTU0849	Bacteria (100)	Firmicutes (100)	Bacilli (93)	Bacillales (85)	Unclassified (<80)	Unclassified (<80)
OTU0850	Bacteria (100)	Firmicutes (100)	Bacilli (100)	Bacillales (100)	Bacillales incertae sedis XII (99)	*Exiguobacterium (99)*
OTU0851	Bacteria (100)	Proteobacteria (100)	Gammaproteobacteria (100)	Enterobacteriales (100)	Enterobacteriaceae (100)	Unclassified (<80)
OTU0852	Bacteria (100)	Proteobacteria (100)	Gammaproteobacteria (100)	Enterobacteriales (99)	Enterobacteriaceae (99)	Unclassified (<80)
OTU0853	Bacteria (100)	Actinobacteria (100)	Actinobacteria (100)	Pseudonocardiales (100)	Pseudonocardiaceae (100)	*Saccharopolyspora (99)*
OTU0854	Bacteria (100)	Proteobacteria (100)	Gammaproteobacteria (100)	Enterobacteriales (100)	Enterobacteriaceae (100)	Unclassified (<80)
OTU0855	Bacteria (100)	Proteobacteria (100)	Gammaproteobacteria (100)	Enterobacteriales (100)	Enterobacteriaceae (100)	Unclassified (<80)
OTU0856	Bacteria (100)	Proteobacteria (100)	Gammaproteobacteria (100)	Vibrionales (100)	Vibrionaceae (100)	*Vibrio (100)*
OTU0857	Bacteria (100)	Proteobacteria (100)	Gammaproteobacteria (100)	Xanthomonadales (100)	Xanthomonadaceae (100)	*Stenotrophomonas (96)*
OTU0858	Bacteria (100)	Proteobacteria (100)	Gammaproteobacteria (100)	Pseudomonadales (95)	Pseudomonadaceae (95)	Unclassified (<80)
OTU0859	Bacteria (100)	Firmicutes (100)	Bacilli (99)	Bacillales (98)	Unclassified (<80)	Unclassified (<80)
OTU0860	Bacteria (100)	Actinobacteria (100)	Actinobacteria (100)	Streptomycetales (100)	Streptomycetaceae (100)	Unclassified (<80)
OTU0861	Bacteria (100)	Firmicutes (100)	Bacilli (100)	Bacillales (100)	Bacillaceae 2 (99)	*Ornithinibacillus (86)*
OTU0862	Bacteria (100)	Proteobacteria (100)	Alphaproteobacteria (100)	Rhodobacterales (100)	Rhodobacteraceae (100)	Unclassified (<80)
OTU0863	Bacteria (100)	Proteobacteria (100)	Betaproteobacteria (100)	Burkholderiales (99)	Unclassified (<80)	Unclassified (<80)
OTU0864	Bacteria (100)	Firmicutes (100)	Bacilli (100)	Bacillales (100)	Unclassified (<80)	Unclassified (<80)
OTU0865	Bacteria (100)	Proteobacteria (100)	Betaproteobacteria (100)	Unclassified (<80)	Unclassified (<80)	Unclassified (<80)
OTU0866	Bacteria (100)	Actinobacteria (100)	Actinobacteria (100)	Pseudonocardiales (100)	Pseudonocardiaceae (98)	Unclassified (<80)
OTU0867	Bacteria (100)	Proteobacteria (100)	Gammaproteobacteria (100)	Pseudomonadales (100)	Pseudomonadaceae (100)	Unclassified (<80)
OTU0868	Bacteria (100)	Actinobacteria (100)	Actinobacteria (100)	Micrococcales (100)	Microbacteriaceae (100)	Unclassified (<80)
OTU0869	Bacteria (100)	Acidobacteria (100)	Acidobacteria_Gp4 (100)	--------†	--------†	*Aridibacter (100)*
OTU0870	Bacteria (100)	Firmicutes (100)	Bacilli (100)	Bacillales (100)	Staphylococcaceae (100)	*Staphylococcus (86)*

#OTU	DOMAIN (%)	PHYLUM (%)	CLASS/SUBCLASS (%)	ORDER (%)	FAMILY (%)	*GENUS (%)*
OTU0884	Bacteria (100)	Firmicutes (100)	Bacilli (100)	Lactobacillales (100)	Enterococcaceae (100)	Enterococcus (99)
OTU0885	Bacteria (100)	Actinobacteria (100)	Actinobacteria (100)	Micrococcales (100)	Microbacteriaceae (100)	*Agromyces (100)*
OTU0886	Bacteria (100)	Proteobacteria (100)	Gammaproteobacteria (100)	Oceanospirillales (100)	Oceanospirillaceae (100)	*Marinobacterium (99)*
OTU0887	Bacteria (100)	Cyanobac./Chloroplast (100)	-------	-------	-------	-------
OTU0888	Bacteria (100)	Proteobacteria (100)	Gammaproteobacteria (100)	Pseudomonadales (100)	Pseudomonadaceae (100)	*Pseudomonas (96)*
OTU0889	Bacteria (100)	Firmicutes (100)	Bacilli (93)	Bacillales (89)	Unclassified (<80)	Unclassified (<80)
OTU0890	Bacteria (100)	Actinobacteria (100)	Actinobacteria (100)	Micrococcales (100)	Brevibacteriaceae (100)	*Brevibacterium (100)*
OTU0891	Bacteria (100)	Proteobacteria (100)	Gammaproteobacteria (100)	Pseudomonadales (100)	Pseudomonadaceae (100)	*Azotobacter (100)*
OTU0892	Bacteria (100)	Proteobacteria (100)	Gammaproteobacteria (100)	Xanthomonadales (100)	Xanthomonadaceae (100)	*Pseudoxanthomonas (100)*
OTU0893	Bacteria (100)	Actinobacteria (100)	Actinobacteria (100)	Corynebacteriales (100)	Dietziaceae (97)	*Dietzia (97)*
OTU0894	Bacteria (100)	Proteobacteria (100)	Betaproteobacteria (100)	Burkholderiales (99)	Comamonadaceae (98)	*Schlegelella (95)*
OTU0895	Bacteria (100)	Proteobacteria (100)	Gammaproteobacteria (100)	Oceanospirillales (100)	Halomonadaceae (100)	*Halomonas (96)*
OTU0896	Bacteria (100)	Proteobacteria (100)	Gammaproteobacteria (99)	Pseudomonadales (99)	Pseudomonadaceae (99)	*Pseudomonas (97)*
OTU0897	Bacteria (100)	Proteobacteria (100)	Gammaproteobacteria (100)	Aeromonadales (100)	Aeromonadaceae (100)	*Zobellella (97)*
OTU0898	Bacteria (100)	Proteobacteria (100)	Gammaproteobacteria (100)	Pseudomonadales (100)	Pseudomonadaceae (100)	*Pseudomonas (98)*
OTU0899	Bacteria (100)	Actinobacteria (100)	Actinobacteria (100)	Pseudonocardiales (100)	Pseudonocardiaceae (100)	*Pseudonocardia (99)*
OTU0900	Bacteria (100)	Firmicutes (100)	Bacilli (100)	Bacillales (100)	Bacillaceae 1 (94)	*Bacillus (80)*
OTU0901	Bacteria (100)	Proteobacteria (100)	Alphaproteobacteria (100)	Rhizobiales (100)	Methylobacteriaceae (100)	*Microvirga (100)*
OTU0902	Bacteria (100)	Proteobacteria (100)	Alphaproteobacteria (100)	Sphingomonadales (100)	Sphingomonadaceae (100)	*Sphingomonas (98)*
OTU0903	Bacteria (100)	Actinobacteria (100)	Actinobacteria (100)	Micrococcales (100)	Micrococcaceae (99)	*Kocuria (96)*
OTU0904	Bacteria (100)	Proteobacteria (99)	Alphaproteobacteria (99)	Unclassified (<80)	Unclassified (<80)	Unclassified (<80)
OTU0905	Bacteria (100)	Actinobacteria (100)	Actinobacteria (100)	Micrococcales (100)	Microbacteriaceae (100)	*Microbacterium (91)*
OTU0906	Bacteria (100)	Firmicutes (100)	Bacilli (100)	Bacillales (100)	Paenibacillaceae 1 (100)	*Paenibacillus (96)*
OTU0907	Bacteria (100)	Deinococcus-Thermus (100)	Deinococci (100)	Deinococcales (100)	Deinococcaceae (100)	*Deinococcus (100)*
OTU0908	Bacteria (100)	Firmicutes (100)	Bacilli (100)	Bacillales (100)	Bacillaceae 2 (100)	*Oceanobacillus (100)*
OTU0909	Bacteria (100)	Proteobacteria (100)	Betaproteobacteria (100)	Burkholderiales (98)	Unclassified (<80)	Unclassified (<80)
OTU0910	Bacteria (100)	Actinobacteria (100)	Actinobacteria (100)	Pseudonocardiales (100)	Pseudonocardiaceae (100)	Unclassified (<80)
OTU0911	Bacteria (100)	Firmicutes (100)	Bacilli (100)	Bacillales (100)	Staphylococcaceae (100)	*Nosocomiicoccus (99)*
OTU0912	Bacteria (100)	Proteobacteria (100)	Alphaproteobacteria (100)	Sphingomonadales (100)	Sphingomonadaceae (100)	*Sphingomonas (100)*
OTU0913	Bacteria (100)	Proteobacteria (100)	Alphaproteobacteria (100)	Sphingomonadales (100)	Sphingomonadaceae (100)	*Sphingomonas (100)*
OTU0914	Bacteria (100)	Proteobacteria (100)	Gammaproteobacteria (100)	Pseudomonadales (100)	Pseudomonadaceae (100)	*Pseudomonas (82)*
OTU0915	Bacteria (100)	Firmicutes (100)	Bacilli (100)	Bacillales (100)	Bacillaceae 2 (100)	*Gracilibacillus (99)*
OTU0916	Bacteria (100)	Proteobacteria (100)	Alphaproteobacteria (100)	Sphingomonadales (100)	Sphingomonadaceae (100)	Unclassified (<80)
OTU0917	Bacteria (100)	Actinobacteria (100)	Actinobacteria (100)	Propionibacteriales (100)	Nocardiopsaceae (100)	*Nocardiopsis (94)*
[illegible]	Bacteria	Firmicutes	Bacilli	Bacillales	Planococcaceae	Unclassified

#OTU	DOMAIN (%)	PHYLUM (%)	CLASS/SUBCLASS (%)	ORDER (%)	FAMILY (%)	*GENUS (%)*
OTU0931	Bacteria (100)	Proteobacteria (100)	Alphaproteobacteria (100)	Unclassified (<80)	Unclassified (<80)	Unclassified (<80)
OTU0932	Bacteria (100)	Firmicutes (100)	Bacilli (100)	Lactobacillales (100)	Enterococcaceae (100)	*Enterococcus (100)*
OTU0933	Bacteria (100)	Firmicutes (100)	Bacilli (100)	Lactobacillales (100)	Carnobacteriaceae (97)	*Atopostipes (82)*
OTU0934	Bacteria (100)	Proteobacteria (100)	Gammaproteobacteria (100)	Pseudomonadales (100)	Moraxellaceae (100)	*Acinetobacter (100)*
OTU0935	Bacteria (100)	Firmicutes (100)	Bacilli (100)	Bacillales (100)	Bacillaceae 1 (87)	Unclassified (<80)
OTU0936	Bacteria (100)	Firmicutes (100)	Bacilli (100)	Lactobacillales (100)	Carnobacteriaceae (93)	*Atopostipes (92)*
OTU0937	Bacteria (100)	Firmicutes (100)	Bacilli (100)	Lactobacillales (100)	Streptococcaceae (100)	*Lactococcus (100)*
OTU0938	Bacteria (96)	Unclassified (<80)	Unclassified (<80)	Unclassified (<80)	Unclassified (<80)	Unclassified (<80)
OTU0939	Bacteria (100)	Actinobacteria (100)	Actinobacteria (100)	Micrococcales (100)	Micrococcaceae (99)	*Rothia (99)*
OTU0940	Bacteria (100)	Firmicutes (100)	Bacilli (100)	Lactobacillales (94)	Unclassified (<80)	Unclassified (<80)
OTU0941	Bacteria (100)	Proteobacteria (100)	Gammaproteobacteria (100)	Alteromonadales (100)	Psychromonadaceae (100)	*Psychromonas (100)*
OTU0942	Bacteria (100)	Proteobacteria (100)	Gammaproteobacteria (100)	Vibrionales (100)	Vibrionaceae (100)	*Photobacterium (100)*
OTU0943	Bacteria (100)	Actinobacteria (100)	Actinobacteria (100)	Propionibacteriales (100)	Nocardioidaceae (100)	*Nocardioides (99)*
OTU0944	Bacteria (100)	Firmicutes (100)	Bacilli (100)	Bacillales (100)	Planococcaceae (100)	Unclassified (<80)
OTU0945	Bacteria (100)	Proteobacteria (100)	Gammaproteobacteria (100)	Enterobacteriales (97)	Enterobacteriaceae (97)	Unclassified (<80)
OTU0946	Bacteria (100)	Firmicutes (100)	Bacilli (100)	Lactobacillales (99)	Unclassified (<80)	Unclassified (<80)
OTU0947	Bacteria (100)	Proteobacteria (100)	Betaproteobacteria (100)	Burkholderiales (98)	Burkholderiaceae (98)	*Burkholderia (93)*
OTU0948	Bacteria (100)	Firmicutes (100)	Bacilli (100)	Bacillales (99)	Bacillaceae 1 (89)	*Bacillus (89)*
OTU0949	Bacteria (99)	Cyanobac./Chloroplast (100)	-------	-------	-------	-------
OTU0950	Bacteria (100)	Firmicutes (100)	Bacilli (100)	Bacillales (99)	Staphylococcaceae (99)	Unclassified (<80)
OTU0951	Bacteria (100)	Firmicutes (100)	Bacilli (100)	Bacillales (100)	Bacillaceae 1 (90)	Unclassified (<80)
OTU0952	Bacteria (100)	Proteobacteria (100)	Gammaproteobacteria (100)	Enterobacteriales (100)	Enterobacteriaceae (100)	*Escherichia/Shigella (82)*
OTU0953	Bacteria (100)	Proteobacteria (99)	Gammaproteobacteria (99)	Pseudomonadales (98)	Moraxellaceae (98)	Unclassified (<80)
OTU0954	Bacteria (100)	Proteobacteria (100)	Gammaproteobacteria (100)	Enterobacteriales (100)	Enterobacteriaceae (100)	Unclassified (<80)
OTU0955	Bacteria (100)	Proteobacteria (100)	Gammaproteobacteria (100)	Oceanospirillales (100)	Halomonadaceae (100)	*Salinicola (94)*
OTU0956	Bacteria (100)	Proteobacteria (100)	Alphaproteobacteria (100)	Rhizobiales (100)	Hyphomicrobiaceae (100)	*Devosia (100)*
OTU0957	Bacteria (100)	Actinobacteria (100)	Actinobacteria (100)	Micrococcales (100)	Microbacteriaceae (100)	Unclassified (<80)
OTU0958	Bacteria (100)	Firmicutes (100)	Clostridia (100)	Clostridiales (100)	Clostridiaceae 1 (99)	*Clostridium sensu st (98)*
OTU0959	Bacteria (100)	Proteobacteria (100)	Gammaproteobacteria (100)	Enterobacteriales (100)	Enterobacteriaceae (100)	*Escherichia/Shigella (82)*
OTU0960	Bacteria (100)	Firmicutes (100)	Clostridia (100)	Clostridiales (100)	Peptostreptococcaceae (100)	Unclassified (<80)
OTU0961	Bacteria (100)	Proteobacteria (100)	Alphaproteobacteria (100)	Rhizobiales (100)	Methylobacteriaceae (98)	*Methylobacterium (98)*
OTU0962	Bacteria (100)	Proteobacteria (100)	Alphaproteobacteria (100)	Sphingomonadales (100)	Sphingomonadaceae (100)	*Sphingomonas (100)*
OTU0963	Bacteria (100)	Proteobacteria (100)	Betaproteobacteria (100)	Methylophilales (100)	Methylophilaceae (100)	Unclassified (<80)
OTU0964	Bacteria (100)	Proteobacteria (100)	Betaproteobacteria (100)	Burkholderiales (100)	Oxalobacteraceae (100)	*Janthinobacterium (94)*
[illegible]	Bacteria [illegible]	Proteobacteria [illegible]	Gammaproteobacteria [illegible]	Enterobacteriales [illegible]	Enterobacteriaceae [illegible]	*Escherichia/Shigella* [illegible]

#OTU	DOMAIN (%)	PHYLUM (%)	CLASS/SUBCLASS (%)	ORDER (%)	FAMILY (%)	*GENUS (%)*
OTU0977	Bacteria (100)	Proteobacteria (100)	Gammaproteobacteria (100)	Pasteurellales (100)	Pasteurellaceae (100)	Unclassified (<80)
OTU0978	Bacteria (100)	Firmicutes (100)	Bacilli (100)	Bacillales (100)	Bacillaceae 1 (86)	Unclassified (<80)
OTU0979	Bacteria (100)	Proteobacteria (100)	Betaproteobacteria (100)	Burkholderiales (82)	Unclassified (<80)	Unclassified (<80)
OTU0980	Bacteria (100)	Proteobacteria (100)	Gammaproteobacteria (100)	Enterobacteriales (100)	Enterobacteriaceae (100)	*Escherichia/Shigella (83)*
OTU0981	Bacteria (100)	Proteobacteria (100)	Alphaproteobacteria (100)	Sphingomonadales (100)	Sphingomonadaceae (100)	*Sphingomonas (98)*
OTU0982	Bacteria (100)	Proteobacteria (100)	Gammaproteobacteria (100)	Xanthomonadales (100)	Xanthomonadaceae (100)	*Lysobacter (100)*
OTU0983	Bacteria (100)	Proteobacteria (100)	Alphaproteobacteria (100)	Rhizobiales (100)	Methylobacteriaceae (100)	*Methylobacterium (99)*
OTU0984	Bacteria (100)	Firmicutes (100)	Clostridia (100)	Clostridiales (100)	Clostridiales incertae sedis XI (100)	*Anaerococcus (100)*
OTU0985	Bacteria (100)	Proteobacteria (100)	Alphaproteobacteria (100)	Rhodobacterales (99)	Rhodobacteraceae (99)	Unclassified (<80)
OTU0986	Bacteria (100)	Proteobacteria (100)	Gammaproteobacteria (100)	Pseudomonadales (100)	Moraxellaceae (100)	*Acinetobacter (100)*
OTU0987	Bacteria (100)	Actinobacteria (100)	Actinobacteria (100)	Propionibacteriales (100)	Propionibacteriaceae (100)	*Propionibacterium (100)*
OTU0988	Bacteria (100)	Proteobacteria (100)	Gammaproteobacteria (100)	Xanthomonadales (100)	Xanthomonadaceae (100)	*Stenotrophomonas (100)*
OTU0989	Bacteria (100)	Proteobacteria (100)	Deltaproteobacteria (100)	Desulfobacterales (100)	Desulfobacteraceae (100)	Unclassified (<80)
OTU0990	Bacteria (100)	Firmicutes (100)	Bacilli (100)	Lactobacillales (97)	Enterococcaceae (80)	Unclassified (<80)
OTU0991	Bacteria (100)	Firmicutes (100)	Bacilli (100)	Lactobacillales (100)	Streptococcaceae (100)	*Streptococcus (100)*
OTU0992	Bacteria (100)	Proteobacteria (100)	Gammaproteobacteria (100)	Enterobacteriales (98)	Enterobacteriaceae (98)	Unclassified (<80)
OTU0993	Bacteria (100)	Actinobacteria (100)	Actinobacteria (100)	Propionibacteriales (100)	Propionibacteriaceae (100)	*Tessaracoccus (95)*
OTU0994	Bacteria (100)	Actinobacteria (100)	Actinobacteria (100)	Pseudonocardiales (100)	Pseudonocardiaceae (100)	*Pseudonocardia (96)*
OTU0995	Bacteria (100)	Actinobacteria (100)	Actinobacteria (100)	Propionibacteriales (100)	Nocardioidaceae (100)	*Nocardioides (95)*
OTU0996	Bacteria (100)	Unclassified (<80)	Unclassified (<80)	Unclassified (<80)	Unclassified (<80)	Unclassified (<80)
OTU0997	Bacteria (100)	Actinobacteria (100)	Actinobacteria (100)	Pseudonocardiales (100)	Pseudonocardiaceae (100)	*Amycolatopsis (92)*
OTU0998	Bacteria (100)	Actinobacteria (100)	Actinobacteria (100)	Propionibacteriales (100)	Promicromonosporaceae (95)	Unclassified (<80)
OTU0999	Bacteria (100)	Firmicutes (100)	Bacilli (100)	Bacillales (100)	Unclassified (<80)	Unclassified (<80)
OTU1000	Bacteria (100)	Proteobacteria (100)	Gammaproteobacteria (100)	Enterobacteriales (100)	Enterobacteriaceae (100)	Unclassified (<80)
OTU1001	Bacteria (100)	Proteobacteria (100)	Gammaproteobacteria (99)	Pseudomonadales (97)	Pseudomonadaceae (97)	*Pseudomonas (86)*
OTU1002	Bacteria (100)	Proteobacteria (100)	Alphaproteobacteria (100)	Rhodobacterales (100)	Rhodobacteraceae (100)	Unclassified (<80)
OTU1003	Bacteria (100)	Proteobacteria (100)	Alphaproteobacteria (100)	Rhizobiales (100)	Hyphomicrobiaceae (100)	*Devosia (91)*
OTU1004	Bacteria (100)	Proteobacteria (100)	Gammaproteobacteria (100)	Enterobacteriales (100)	Enterobacteriaceae (100)	Unclassified (<80)
OTU1005	Bacteria (94)	Unclassified (<80)	Unclassified (<80)	Unclassified (<80)	Unclassified (<80)	Unclassified (<80)
OTU1006	Bacteria (100)	Proteobacteria (100)	Gammaproteobacteria (100)	Oceanospirillales (100)	Halomonadaceae (100)	*Halomonas (99)*
OTU1007	Bacteria (100)	Firmicutes (100)	Bacilli (100)	Bacillales (94)	Staphylococcaceae (94)	*Staphylococcus (94)*
OTU1008	Bacteria (100)	Proteobacteria (100)	Alphaproteobacteria (100)	Sphingomonadales (100)	Sphingomonadaceae (100)	*Novosphingobium (91)*
OTU1009	Bacteria (100)	Proteobacteria (100)	Alphaproteobacteria (100)	Sphingomonadales (100)	Sphingomonadaceae (100)	*Sphingomonas (97)*
OTU1010	Bacteria (100)	Actinobacteria (100)	Actinobacteria (100)	Propionibacteriales (100)	Nocardioidaceae (100)	*Nocardioides (100)*
[illegible]	Bacteria [illegible]	Firmicutes [illegible]	Bacilli [illegible]	Bacillales [illegible]	Planococcaceae [illegible]	Unclassified [illegible]

#OTU	DOMAIN (%)	PHYLUM (%)	CLASS/SUBCLASS (%)	ORDER (%)	FAMILY (%)	*GENUS (%)*
OTU1025	Bacteria (100)	Firmicutes (100)	Bacilli (100)	Lactobacillales (96)	Unclassified (<80)	Unclassified (<80)
OTU1026	Bacteria (100)	Firmicutes (100)	Bacilli (100)	Lactobacillales (87)	Unclassified (<80)	Unclassified (<80)
OTU1027	Bacteria (100)	Firmicutes (100)	Bacilli (100)	Lactobacillales (94)	Unclassified (<80)	Unclassified (<80)
OTU1028	Bacteria (100)	Proteobacteria (100)	Gammaproteobacteria (99)	Enterobacteriales (96)	Enterobacteriaceae (96)	Unclassified (<80)
OTU1029	Bacteria (100)	Proteobacteria (100)	Gammaproteobacteria (100)	Enterobacteriales (100)	Enterobacteriaceae (100)	*Escherichia/Shigella (83)*
OTU1030	Bacteria (100)	Proteobacteria (100)	Gammaproteobacteria (100)	Enterobacteriales (100)	Enterobacteriaceae (100)	*Escherichia/Shigella (83)*
OTU1031	Bacteria (100)	Actinobacteria (100)	Actinobacteria (100)	Corynebacteriales (100)	Corynebacteriaceae (100)	*Corynebacterium (100)*
OTU1032	Bacteria (100)	Proteobacteria (100)	Gammaproteobacteria (100)	Enterobacteriales (99)	Enterobacteriaceae (99)	*Unclassified (<80)*
OTU1033	Bacteria (100)	Proteobacteria (100)	Gammaproteobacteria (100)	Oceanospirillales (100)	Halomonadaceae (100)	*Salinicola (97)*
OTU1034	Bacteria (100)	Firmicutes (100)	Bacilli (100)	Bacillales (100)	Bacillaceae 2 (100)	*Oceanobacillus (92)*
OTU1035	Bacteria (100)	Proteobacteria (100)	Gammaproteobacteria (100)	Enterobacteriales (100)	Enterobacteriaceae (100)	Unclassified (<80)
OTU1036	Bacteria (100)	Proteobacteria (100)	Gammaproteobacteria (100)	Enterobacteriales (100)	Enterobacteriaceae (100)	Unclassified (<80)
OTU1037	Bacteria (100)	Proteobacteria (100)	Alphaproteobacteria (100)	Sphingomonadales (99)	Sphingomonadaceae (99)	*Sphingomonas (95)*
OTU1038	Bacteria (100)	Proteobacteria (100)	Gammaproteobacteria (100)	Xanthomonadales (100)	Xanthomonadaceae (100)	*Thermomonas (99)*
OTU1039	Bacteria (100)	Actinobacteria (100)	Actinobacteria (100)	Corynebacteriales (100)	Dietziaceae (100)	*Dietzia (100)*
OTU1040	Bacteria (100)	Proteobacteria (100)	Gammaproteobacteria (100)	Pseudomonadales (100)	Pseudomonadaceae (100)	*Azotobacter (99)*
OTU1041	Bacteria (100)	Proteobacteria (100)	Gammaproteobacteria (100)	Alteromonadales (100)	Shewanellaceae (100)	*Shewanella (100)*
OTU1042	Bacteria (100)	Actinobacteria (100)	Actinobacteria (100)	Micrococcales (100)	Unclassified (<80)	Unclassified (<80)
OTU1043	Bacteria (100)	Proteobacteria (100)	Alphaproteobacteria (100)	Rhodobacterales (83)	Rhodobacteraceae (83)	Unclassified (<80)
OTU1044	Bacteria (100)	Firmicutes (100)	Bacilli (98)	Bacillales (94)	Staphylococcaceae (88)	*Staphylococcus (82)*
OTU1045	Bacteria (100)	Proteobacteria (100)	Gammaproteobacteria (100)	Enterobacteriales (100)	Enterobacteriaceae (100)	*Escherichia/Shigella (80)*
OTU1046	Bacteria (100)	Proteobacteria (100)	Gammaproteobacteria (100)	Oceanospirillales (100)	Halomonadaceae (100)	*Halomonas (100)*
OTU1047	Bacteria (100)	Proteobacteria (100)	Betaproteobacteria (100)	Burkholderiales (100)	Burkholderiaceae (98)	*Burkholderia (95)*
OTU1048	Bacteria (100)	Proteobacteria (100)	Betaproteobacteria (100)	Burkholderiales (99)	Burkholderiaceae (99)	*Burkholderia (97)*
OTU1049	Bacteria (100)	Firmicutes (100)	Bacilli (100)	Bacillales (100)	Planococcaceae (92)	*Lysinibacillus (84)*
OTU1050	Bacteria (100)	Firmicutes (100)	Bacilli (100)	Bacillales (100)	Unclassified (<80)	Unclassified (<80)
OTU1051	Bacteria (100)	Proteobacteria (100)	Alphaproteobacteria (100)	Sphingomonadales (94)	Sphingomonadaceae (93)	Unclassified (<80)
OTU1052	Bacteria (100)	Proteobacteria (100)	Gammaproteobacteria (100)	Enterobacteriales (100)	Enterobacteriaceae (100)	*Escherichia/Shigella (84)*
OTU1053	Bacteria (100)	Actinobacteria (100)	Actinobacteria (100)	Propionibacteriales (100)	Propionibacteriaceae (100)	*Propionibacterium (100)*
OTU1054	Bacteria (100)	Proteobacteria (100)	Alphaproteobacteria (100)	Rhodobacterales (100)	Rhodobacteraceae (100)	*Oceanicola (90)*
OTU1055	Bacteria (100)	Firmicutes (100)	Bacilli (100)	Bacillales (100)	Planococcaceae (100)	*Chryseomicrobium (88)*
OTU1056	Bacteria (100)	Proteobacteria (100)	Alphaproteobacteria (100)	Unclassified (<80)	Unclassified (<80)	Unclassified (<80)
OTU1057	Bacteria (100)	Proteobacteria (100)	Gammaproteobacteria (100)	Enterobacteriales (100)	Enterobacteriaceae (100)	Unclassified (<80)
OTU1058	Bacteria (100)	Firmicutes (100)	Bacilli (100)	Lactobacillales (100)	Carnobacteriaceae (100)	*Atopostipes (99)*
[illegible]	Bacteria [illegible]	Actinobacteria [illegible]	Actinobacteria [illegible]	Propionibacteriales [illegible]	Nocardiopsaceae [illegible]	*Nocardiopsis* [illegible]

#OTU	DOMAIN (%)	PHYLUM (%)	CLASS/SUBCLASS (%)	ORDER (%)	FAMILY (%)	*GENUS (%)*
OTU1072	Bacteria (100)	Proteobacteria (100)	Gammaproteobacteria (100)	Pseudomonadales (93)	Pseudomonadaceae (93)	Unclassified (<80)
OTU1073	Bacteria (100)	Bacteroidetes (100)	Flavobacteriia (100)	Flavobacteriales (100)	Flavobacteriaceae (100)	*Chryseobacterium (100)*
OTU1074	Bacteria (100)	Proteobacteria (100)	Alphaproteobacteria (100)	Sphingomonadales (100)	Sphingomonadaceae (100)	*Sphingomonas (97)*
OTU1075	Bacteria (100)	Proteobacteria (100)	Gammaproteobacteria (100)	Chromatiales (100)	Chromatiaceae (100)	*Rheinheimera (100)*
OTU1076	Bacteria (100)	Firmicutes (100)	Bacilli (100)	Bacillales (100)	Unclassified (<80)	Unclassified (<80)
OTU1077	Bacteria (100)	Firmicutes (100)	Bacilli (100)	Lactobacillales (100)	Streptococcaceae (99)	*Streptococcus (99)*
OTU1078	Bacteria (100)	Actinobacteria (100)	Actinobacteria (100)	Pseudonocardiales (100)	Pseudonocardiaceae (100)	Unclassified (<80)
OTU1079	Bacteria (100)	Actinobacteria (100)	Actinobacteria (100)	Pseudonocardiales (100)	Pseudonocardiaceae (100)	*Pseudonocardia (99)*
OTU1080	Bacteria (100)	Proteobacteria (100)	Gammaproteobacteria (100)	Enterobacteriales (100)	Enterobacteriaceae (100)	Unclassified (<80)
OTU1081	Bacteria (100)	Proteobacteria (100)	Betaproteobacteria (100)	Burkholderiales (100)	Oxalobacteraceae (100)	*Massilia (97)*
OTU1082	Bacteria (100)	Firmicutes (100)	Bacilli (100)	Bacillales (100)	Unclassified (<80)	Unclassified (<80)
OTU1083	Bacteria (100)	Bacteroidetes (100)	Cytophagia (100)	Cytophagales (100)	Cytophagaceae (100)	*Fibrella (100)*
OTU1084	Bacteria (100)	Proteobacteria (100)	Alphaproteobacteria (100)	Rhizobiales (100)	Rhizobiaceae (97)	Unclassified (<80)
OTU1085	Bacteria (100)	Proteobacteria (99)	Gammaproteobacteria (99)	Pseudomonadales (82)	Pseudomonadaceae (82)	Unclassified (<80)
OTU1086	Bacteria (100)	Proteobacteria (100)	Gammaproteobacteria (100)	Oceanospirillales (100)	Halomonadaceae (100)	Unclassified (<80)
OTU1087	Bacteria (100)	Firmicutes (100)	Bacilli (100)	Bacillales (96)	Unclassified (<80)	Unclassified (<80)
OTU1088	Bacteria (100)	Actinobacteria (100)	Actinobacteria (100)	Micrococcales (100)	Microbacteriaceae (100)	*Cryobacterium (93)*
OTU1089	Bacteria (100)	Proteobacteria (100)	Alphaproteobacteria (100)	Sphingomonadales (100)	Sphingomonadaceae (100)	*Sphingomonas (95)*
OTU1090	Bacteria (100)	Proteobacteria (100)	Alphaproteobacteria (100)	Rhizobiales (100)	Methylobacteriaceae (100)	*Methylobacterium (99)*
OTU1091	Bacteria (100)	Proteobacteria (100)	Alphaproteobacteria (100)	Sphingomonadales (100)	Sphingomonadaceae (100)	*Sphingomonas (95)*
OTU1092	Bacteria (100)	Firmicutes (100)	Bacilli (100)	Bacillales (100)	Bacillaceae 2 (100)	*Oceanobacillus (100)*
OTU1093	Bacteria (100)	Proteobacteria (100)	Gammaproteobacteria (100)	Pseudomonadales (98)	Pseudomonadaceae (98)	Unclassified (<80)
OTU1094	Bacteria (100)	Firmicutes (100)	Bacilli (100)	Bacillales (100)	Planococcaceae (100)	*Lysinibacillus (83)*
OTU1095	Bacteria (100)	Actinobacteria (100)	Actinobacteria (100)	Pseudonocardiales (100)	Pseudonocardiaceae (99)	Unclassified (<80)
OTU1096	Bacteria (100)	Proteobacteria (100)	Betaproteobacteria (100)	Burkholderiales (100)	Burkholderiaceae (100)	*Burkholderia (100)*
OTU1097	Bacteria (100)	Proteobacteria (100)	Betaproteobacteria (100)	Burkholderiales (100)	Burkholderiaceae (98)	*Burkholderia (92)*
OTU1098	Bacteria (100)	Firmicutes (100)	Bacilli (99)	Bacillales (98)	Unclassified (<80)	Unclassified (<80)
OTU1099	Bacteria (100)	Actinobacteria (100)	Actinobacteria (100)	Micrococcales (100)	Micrococcaceae (100)	*Rothia (100)*
OTU1100	Bacteria (100)	Proteobacteria (100)	Betaproteobacteria (100)	Burkholderiales (100)	Unclassified (<80)	Unclassified (<80)
OTU1101	Bacteria (100)	Firmicutes (100)	Bacilli (100)	Bacillales (100)	Staphylococcaceae (100)	*Staphylococcus (100)*
OTU1102	Bacteria (100)	Firmicutes (100)	Bacilli (100)	Bacillales (100)	Staphylococcaceae (100)	*Staphylococcus (100)*
OTU1103	Bacteria (100)	Proteobacteria (100)	Betaproteobacteria (98)	Burkholderiales (98)	Unclassified (<80)	Unclassified (<80)
OTU1104	Bacteria (100)	Actinobacteria (100)	Actinobacteria (100)	Pseudonocardiales (100)	Pseudonocardiaceae (98)	Unclassified (<80)

#OTU	DOMAIN (%)	PHYLUM (%)	CLASS/SUBCLASS (%)	ORDER (%)	FAMILY (%)	*GENUS (%)*
OTU1118	Bacteria (100)	Proteobacteria (100)	Betaproteobacteria (100)	Burkholderiales (100)	Oxalobacteraceae (100)	*Janthinobacterium (96)*
OTU1119	Bacteria (100)	Firmicutes (100)	Bacilli (100)	Bacillales (100)	Planococcaceae (88)	Unclassified (<80)
OTU1120	Bacteria (100)	Firmicutes (100)	Bacilli (100)	Bacillales (98)	Staphylococcaceae (98)	*Staphylococcus (96)*
OTU1121	Bacteria (100)	Firmicutes (100)	Bacilli (100)	Bacillales (100)	Paenibacillaceae 1 (100)	*Paenibacillus (99)*
OTU1122	Bacteria (100)	Proteobacteria (100)	Betaproteobacteria (100)	Burkholderiales (100)	Oxalobacteraceae (100)	*Undibacterium (99)*
OTU1123	Bacteria (100)	Firmicutes (100)	Clostridia (100)	Clostridiales (100)	Lachnospiraceae (100)	*Lachnoanaerobaculu (100)*
OTU1124	Bacteria (100)	Actinobacteria (100)	Actinobacteria (100)	Geodermatophilales (100)	Geodermatophilaceae (100)	*Modestobacter (98)*
OTU1125	Bacteria (100)	Proteobacteria (100)	Gammaproteobacteria (100)	Pseudomonadales (100)	Pseudomonadaceae (100)	*Pseudomonas (96)*
OTU1126	Bacteria (100)	Actinobacteria (100)	Actinobacteria (100)	Micrococcales (100)	Microbacteriaceae (100)	*Curtobacterium (90)*
OTU1127	Bacteria (100)	Actinobacteria (100)	Actinobacteria (100)	Corynebacteriales (100)	Corynebacteriaceae (100)	*Corynebacterium (100)*
OTU1128	Bacteria (100)	Proteobacteria (100)	Betaproteobacteria (100)	Methylophilales (100)	Methylophilaceae (100)	Unclassified (<80)
OTU1129	Bacteria (100)	Firmicutes (100)	Bacilli (100)	Bacillales (98)	Unclassified (<80)	Unclassified (<80)
OTU1130	Bacteria (100)	Proteobacteria (100)	Alphaproteobacteria (100)	Rhizobiales (100)	Unclassified (<80)	Unclassified (<80)
OTU1131	Bacteria (100)	Firmicutes (100)	Bacilli (100)	Bacillales (100)	Planococcaceae (97)	Unclassified (<80)
OTU1132	Bacteria (100)	Cyanobac./Chloroplast (100)	-------	-------	-------	-------
OTU1133	Bacteria (100)	Firmicutes (100)	Bacilli (98)	Bacillales (97)	Bacillaceae 2 (85)	Unclassified (<80)
OTU1134	Bacteria (100)	Proteobacteria (100)	Gammaproteobacteria (100)	Enterobacteriales (100)	Enterobacteriaceae (100)	*Escherichia/Shigella (87)*
OTU1135	Bacteria (100)	Proteobacteria (100)	Betaproteobacteria (100)	Rhodocyclales (84)	Rhodocyclaceae (84)	Unclassified (<80)
OTU1136	Bacteria (100)	Proteobacteria (100)	Alphaproteobacteria (100)	Sphingomonadales (100)	Sphingomonadaceae (100)	Unclassified (<80)
OTU1137	Bacteria (100)	Proteobacteria (100)	Betaproteobacteria (100)	Burkholderiales (100)	Burkholderiaceae (99)	*Burkholderia (98)*
OTU1138	Bacteria (100)	Proteobacteria (100)	Gammaproteobacteria (100)	Pseudomonadales (97)	Pseudomonadaceae (97)	Unclassified (<80)
OTU1139	Bacteria (100)	Firmicutes (100)	Bacilli (100)	Bacillales (100)	Unclassified (<80)	Unclassified (<80)
OTU1140	Bacteria (100)	Firmicutes (100)	Bacilli (100)	Bacillales (100)	Bacillaceae 1 (94)	*Anoxybacillus (93)*
OTU1141	Bacteria (100)	Actinobacteria (100)	Actinobacteria (100)	Streptomycetales (100)	Streptomycetaceae (100)	Unclassified (<80)
OTU1142	Bacteria (100)	Proteobacteria (100)	Gammaproteobacteria (100)	Pseudomonadales (100)	Moraxellaceae (100)	*Acinetobacter (100)*
OTU1143	Bacteria (100)	Firmicutes (100)	Bacilli (100)	Bacillales (100)	Bacillales incertae sedis XI (100)	*Gemella (100)*
OTU1144	Bacteria (100)	Firmicutes (100)	Bacilli (100)	Bacillales (100)	Unclassified (<80)	Unclassified (<80)
OTU1145	Bacteria (100)	Firmicutes (100)	Bacilli (100)	Bacillales (100)	Planococcaceae (99)	*Sporosarcina (99)*
OTU1146	Bacteria (100)	Proteobacteria (100)	Alphaproteobacteria (100)	Rhizobiales (100)	Rhizobiaceae (100)	*Rhizobium (100)*
OTU1147	Bacteria (100)	Firmicutes (100)	Bacilli (100)	Lactobacillales (100)	Unclassified (<80)	Unclassified (<80)
OTU1148	Bacteria (100)	Bacteroidetes (100)	Sphingobacteriia (100)	Sphingobacteriales (100)	Sphingobacteriaceae (100)	*Pedobacter (100)*
OTU1149	Bacteria (100)	Actinobacteria (100)	Actinobacteria (100)	Micrococcales (100)	Microbacteriaceae (100)	Unclassified (<80)
OTU1150	Bacteria (100)	Actinobacteria (100)	Actinobacteria (100)	Micrococcales (100)	Microbacteriaceae (100)	Unclassified (<80)

#OTU	DOMAIN (%)	PHYLUM (%)	CLASS/SUBCLASS (%)	ORDER (%)	FAMILY (%)	*GENUS (%)*
OTU1165	Bacteria (100)	Proteobacteria (99)	Gammaproteobacteria (99)	Oceanospirillales (96)	Halomonadaceae (95)	Unclassified (<80)
OTU1166	Bacteria (100)	Proteobacteria (100)	Gammaproteobacteria (100)	Pseudomonadales (100)	Pseudomonadaceae (100)	*Pseudomonas (100)*
OTU1167	Bacteria (100)	Firmicutes (100)	Bacilli (100)	Lactobacillales (100)	Enterococcaceae (100)	*Pilibacter (100)*
OTU1168	Bacteria (100)	Proteobacteria (100)	Gammaproteobacteria (100)	Xanthomonadales (100)	Xanthomonadaceae (100)	*Luteimonas (96)*
OTU1169	Bacteria (100)	Proteobacteria (100)	Gammaproteobacteria (100)	Pasteurellales (100)	Pasteurellaceae (100)	Unclassified (<80)
OTU1170	Bacteria (100)	Proteobacteria (100)	Alphaproteobacteria (100)	Caulobacterales (100)	Caulobacteraceae (100)	*Brevundimonas (100)*
OTU1171	Bacteria (100)	Actinobacteria (100)	Actinobacteria (100)	Micrococcales (100)	Micrococcaceae (100)	*Arthrobacter (98)*
OTU1172	Bacteria (100)	Firmicutes (100)	Bacilli (100)	Lactobacillales (100)	Enterococcaceae (98)	Unclassified (<80)
OTU1173	Bacteria (100)	Actinobacteria (100)	Actinobacteria (100)	Frankiales (100)	Acidothermaceae (100)	*Acidothermus (100)*
OTU1174	Bacteria (100)	Proteobacteria (100)	Betaproteobacteria (100)	Methylophilales (100)	Methylophilaceae (100)	Unclassified (<80)
OTU1175	Bacteria (100)	Actinobacteria (100)	Actinobacteria (100)	Micrococcales (100)	Microbacteriaceae (100)	Unclassified (<80)
OTU1176	Bacteria (100)	Firmicutes (100)	Bacilli (100)	Bacillales (100)	Bacillaceae 2 (96)	Unclassified (<80)
OTU1177	Bacteria (100)	Firmicutes (100)	Bacilli (100)	Lactobacillales (100)	Enterococcaceae (100)	*Enterococcus (99)*
OTU1178	Bacteria (100)	Actinobacteria (100)	Actinobacteria (100)	Pseudonocardiales (100)	Pseudonocardiaceae (100)	Unclassified (<80)
OTU1179	Bacteria (100)	Proteobacteria (100)	Gammaproteobacteria (100)	Pseudomonadales (100)	Moraxellaceae (100)	*Acinetobacter (100)*
OTU1180	Bacteria (100)	Firmicutes (100)	Bacilli (100)	Bacillales (100)	Planococcaceae (86)	Unclassified (<80)
OTU1181	Bacteria (100)	Proteobacteria (100)	Alphaproteobacteria (100)	Rhizobiales (100)	Rhizobiaceae (100)	*Shinella (86)*
OTU1182	Bacteria (100)	Firmicutes (100)	Bacilli (100)	Lactobacillales (100)	Enterococcaceae (100)	*Enterococcus (100)*
OTU1183	Bacteria (100)	Proteobacteria (100)	Gammaproteobacteria (100)	Pseudomonadales (100)	Pseudomonadaceae (100)	*Pseudomonas (87)*
OTU1184	Bacteria (100)	Proteobacteria (100)	Gammaproteobacteria (99)	Unclassified (<80)	Unclassified (<80)	Unclassified (<80)
OTU1185	Bacteria (100)	Firmicutes (100)	Bacilli (100)	Bacillales (100)	Bacillaceae 1 (95)	Unclassified (<80)
OTU1186	Bacteria (100)	Firmicutes (100)	Bacilli (100)	Lactobacillales (100)	Streptococcaceae (100)	*Streptococcus (100)*
OTU1187	Bacteria (100)	Actinobacteria (100)	Actinobacteria (100)	Micrococcales (100)	Microbacteriaceae (98)	Unclassified (<80)
OTU1188	Bacteria (100)	Proteobacteria (100)	Alphaproteobacteria (100)	Rhodospirillales (100)	Rhodospirillaceae (100)	*Caenispirillum (100)*
OTU1189	Bacteria (100)	Proteobacteria (100)	Gammaproteobacteria (100)	Pseudomonadales (99)	Pseudomonadaceae (99)	*Pseudomonas (86)*
OTU1190	Bacteria (100)	Actinobacteria (100)	Actinobacteria (100)	Streptomycetales (100)	Unclassified (<80)	Unclassified (<80)
OTU1191	Bacteria (100)	Proteobacteria (100)	Gammaproteobacteria (100)	Enterobacteriales (100)	Enterobacteriaceae (100)	*Buttiauxella (85)*
OTU1192	Bacteria (100)	Proteobacteria (100)	Gammaproteobacteria (100)	Pseudomonadales (100)	Pseudomonadaceae (100)	*Pseudomonas (99)*
OTU1193	Bacteria (100)	Firmicutes (100)	Clostridia (100)	Clostridiales (100)	Lachnospiraceae (100)	*Clostridium XlVa (98)*
OTU1194	Bacteria (100)	Firmicutes (100)	Clostridia (100)	Clostridiales (100)	Lachnospiraceae (99)	Unclassified (<80)
OTU1195	Bacteria (100)	Proteobacteria (100)	Betaproteobacteria (100)	Burkholderiales (100)	Burkholderiaceae (100)	*Burkholderia (100)*
OTU1196	Bacteria (100)	Firmicutes (100)	Bacilli (100)	Bacillales (100)	Bacillales incertae sedis XII (99)	*Exiguobacterium (99)*
OTU1197	Bacteria (100)	Gemmatimonadetes (97)	Gemmatimonadetes (97)	Gemmatimonadales (97)	Gemmatimonadaceae (97)	*Gemmatimonas (97)*

#OTU	DOMAIN (%)	PHYLUM (%)	CLASS/SUBCLASS (%)	ORDER (%)	FAMILY (%)	*GENUS (%)*
OTU1212	Bacteria (100)	Proteobacteria (100)	Gammaproteobacteria (100)	Pseudomonadales (81)	Pseudomonadaceae (81)	Unclassified (<80)
OTU1213	Bacteria (100)	Proteobacteria (100)	Betaproteobacteria (100)	Neisseriales (100)	Neisseriaceae (100)	Unclassified (<80)
OTU1214	Bacteria (100)	Firmicutes (100)	Bacilli (100)	Lactobacillales (100)	Leuconostocaceae (100)	*Leuconostoc (97)*
OTU1215	Bacteria (100)	Firmicutes (100)	Bacilli (100)	Bacillales (100)	Planococcaceae (97)	Unclassified (<80)
OTU1216	Bacteria (100)	Firmicutes (100)	Bacilli (100)	Bacillales (100)	Planococcaceae (97)	*Planomicrobium (96)*
OTU1217	Bacteria (100)	Proteobacteria (100)	Gammaproteobacteria (100)	Pseudomonadales (100)	Pseudomonadaceae (100)	*Pseudomonas (99)*
OTU1218	Bacteria (100)	Firmicutes (100)	Bacilli (100)	Bacillales (100)	Unclassified (<80)	Unclassified (<80)
OTU1219	Bacteria (100)	Actinobacteria (100)	Actinobacteria (100)	Propionibacteriales (100)	Propionibacteriaceae (100)	*Propionibacterium (94)*
OTU1220	Bacteria (100)	Proteobacteria (100)	Betaproteobacteria (100)	Burkholderiales (99)	Comamonadaceae (83)	*Comamonas (80)*
OTU1221	Bacteria (100)	Actinobacteria (100)	Actinobacteria (100)	Micrococcales (100)	Microbacteriaceae (100)	Unclassified (<80)
OTU1222	Archaea (100)	Euryarchaeota (100)	Halobacteria (100)	Halobacteriales (100)	Halobacteriaceae (100)	*Halobacterium (100)*
OTU1223	Bacteria (100)	Firmicutes (99)	Bacilli (94)	Bacillales (88)	Unclassified (<80)	Unclassified (<80)
OTU1224	Bacteria (100)	Proteobacteria (100)	Gammaproteobacteria (100)	Alteromonadales (100)	Alteromonadaceae (99)	*Alishewanella (99)*
OTU1225	Bacteria (100)	Proteobacteria (100)	Gammaproteobacteria (100)	Enterobacteriales (99)	Enterobacteriaceae (99)	*Proteus (99)*
OTU1226	Bacteria (100)	Firmicutes (100)	Bacilli (100)	Bacillales (100)	Unclassified (<80)	Unclassified (<80)
OTU1227	Bacteria (100)	Firmicutes (100)	Bacilli (90)	Bacillales (90)	Unclassified (<80)	Unclassified (<80)
OTU1228	Bacteria (100)	Proteobacteria (100)	Alphaproteobacteria (100)	Rhizobiales (100)	Phyllobacteriaceae (100)	*Aminobacter (100)*
OTU1229	Bacteria (100)	Cyanobac./Chloroplast (100)	-------	-------	-------	-------
OTU1230	Bacteria (100)	Actinobacteria (100)	Actinobacteria (100)	Pseudonocardiales (100)	Pseudonocardiaceae (100)	*Saccharopolyspora (92)*
OTU1231	Bacteria (100)	Firmicutes (100)	Bacilli (100)	Lactobacillales (98)	Enterococcaceae (98)	Unclassified (<80)
OTU1232	Bacteria (100)	Firmicutes (100)	Bacilli (100)	Lactobacillales (99)	Unclassified (<80)	Unclassified (<80)
OTU1233	Bacteria (100)	Firmicutes (100)	Bacilli (100)	Lactobacillales (94)	Unclassified (<80)	Unclassified (<80)
OTU1234	Bacteria (100)	Proteobacteria (100)	Gammaproteobacteria (100)	Enterobacteriales (100)	Enterobacteriaceae (100)	Unclassified (<80)
OTU1235	Bacteria (100)	Proteobacteria (100)	Betaproteobacteria (100)	Burkholderiales (100)	Comamonadaceae (100)	*Comamonas (96)*
OTU1236	Bacteria (100)	Proteobacteria (100)	Gammaproteobacteria (100)	Enterobacteriales (100)	Enterobacteriaceae (100)	*Pantoea (94)*
OTU1237	Bacteria (100)	Proteobacteria (100)	Gammaproteobacteria (100)	Enterobacteriales (100)	Enterobacteriaceae (100)	Unclassified (<80)
OTU1238	Bacteria (100)	Firmicutes (100)	Negativicutes (100)	Selenomonadales (100)	Veillonellaceae (100)	*Veillonella (100)*
OTU1239	Bacteria (100)	Proteobacteria (97)	Gammaproteobacteria (95)	Enterobacteriales (94)	Enterobacteriaceae (94)	Unclassified (<80)
OTU1240	Bacteria (100)	Proteobacteria (100)	Alphaproteobacteria (100)	Rhodospirillales (100)	Rhodospirillaceae (100)	*Caenispirillum (100)*
OTU1241	Bacteria (100)	Proteobacteria (100)	Gammaproteobacteria (100)	Enterobacteriales (100)	Enterobacteriaceae (100)	*Pantoea (83)*
OTU1242	Bacteria (100)	Firmicutes (100)	Bacilli (100)	Bacillales (100)	Bacillaceae 1 (84)	*Bacillus (80)*
OTU1243	Bacteria (100)	Proteobacteria (100)	Alphaproteobacteria (100)	Rhizobiales (99)	Methylobacteriaceae (97)	*Methylobacterium (97)*
OTU1244	Bacteria (100)	Proteobacteria (100)	Alphaproteobacteria (100)	Rhizobiales (100)	Methylobacteriaceae (100)	*Methylobacterium (100)*

#OTU	DOMAIN (%)	PHYLUM (%)	CLASS/SUBCLASS (%)	ORDER (%)	FAMILY (%)	*GENUS (%)*
OTU1259	Bacteria (100)	Firmicutes (100)	Bacilli (100)	Bacillales (100)	Bacillales incertae sedis XI (100)	*Gemella (100)*
OTU1260	Bacteria (100)	Proteobacteria (100)	Gammaproteobacteria (100)	Pseudomonadales (97)	Pseudomonadaceae (97)	Unclassified (<80)
OTU1261	Bacteria (100)	Proteobacteria (100)	Gammaproteobacteria (100)	Pseudomonadales (97)	Pseudomonadaceae (97)	Unclassified (<80)
OTU1262	Bacteria (100)	Firmicutes (100)	Bacilli (100)	Bacillales (100)	Planococcaceae (100)	Unclassified (<80)
OTU1263	Bacteria (100)	Firmicutes (100)	Clostridia (100)	Clostridiales (100)	Peptostreptococcaceae (100)	*RombOTUsia (98)*
OTU1264	Bacteria (100)	Actinobacteria (100)	Actinobacteria (100)	Micrococcales (100)	Micrococcaceae (99)	Unclassified (<80)
OTU1265	Bacteria (100)	Actinobacteria (100)	Actinobacteria (100)	Micrococcales (100)	Brevibacteriaceae (100)	*Brevibacterium (100)*
OTU1266	Bacteria (100)	Actinobacteria (100)	Actinobacteria (100)	Micrococcales (100)	Micrococcaceae (98)	*Rothia (98)*
OTU1267	Bacteria (100)	Firmicutes (100)	Bacilli (100)	Bacillales (100)	Unclassified (<80)	Unclassified (<80)
OTU1268	Bacteria (100)	Actinobacteria (100)	Actinobacteria (100)	Streptosporangiales (100)	Allonocardiopsis (84)	*Allonocardiopsis (84)*
OTU1269	Bacteria (100)	Firmicutes (100)	Bacilli (100)	Bacillales (100)	Bacillaceae 1 (92)	*Bacillus (92)*
OTU1270	Bacteria (100)	Actinobacteria (100)	Actinobacteria (100)	Pseudonocardiales (100)	Pseudonocardiaceae (100)	*Pseudonocardia (85)*
OTU1271	Bacteria (100)	Proteobacteria (100)	Gammaproteobacteria (100)	Xanthomonadales (100)	Xanthomonadaceae (100)	*Lysobacter (100)*
OTU1272	Bacteria (100)	Firmicutes (100)	Bacilli (100)	Bacillales (100)	Bacillales incertae sedis XII (100)	*Exiguobacterium (100)*
OTU1273	Bacteria (100)	Actinobacteria (100)	Actinobacteria (100)	Micrococcales (100)	Dermabacteraceae (100)	*Brachybacterium (100)*
OTU1274	Bacteria (100)	Proteobacteria (100)	Gammaproteobacteria (100)	Vibrionales (99)	Vibrionaceae (99)	Unclassified (<80)
OTU1275	Bacteria (100)	Firmicutes (100)	Bacilli (100)	Bacillales (100)	Bacillaceae 2 (100)	Unclassified (<80)
OTU1276	Bacteria (100)	Proteobacteria (100)	Gammaproteobacteria (100)	Enterobacteriales (100)	Enterobacteriaceae (100)	Unclassified (<80)
OTU1277	Bacteria (100)	Firmicutes (100)	Bacilli (100)	Lactobacillales (88)	Unclassified (<80)	Unclassified (<80)
OTU1278	Bacteria (100)	Proteobacteria (100)	Betaproteobacteria (100)	Burkholderiales (100)	Oxalobacteraceae (100)	*Massilia (100)*
OTU1279	Bacteria (100)	Proteobacteria (100)	Gammaproteobacteria (100)	Pseudomonadales (100)	Pseudomonadaceae (100)	*Pseudomonas (96)*
OTU1280	Bacteria (100)	Cyanobact./Chloroplast (100)	-------	-------	-------	-------
OTU1281	Bacteria (100)	Acidobacteria (100)	Acidobacteria_Gp4 (100)	Unclassified (<80)	Unclassified (<80)	Unclassified (<80)
OTU1282	Bacteria (100)	Firmicutes (100)	Bacilli (100)	Bacillales (97)	Unclassified (<80)	Unclassified (<80)
OTU1283	Bacteria (100)	Actinobacteria (100)	Actinobacteria (100)	Micrococcales (100)	Microbacteriaceae (100)	Unclassified (<80)
OTU1284	Bacteria (100)	Actinobacteria (100)	Actinobacteria (100)	Micrococcales (100)	Micrococcaceae (100)	*Micrococcus (100)*
OTU1285	Bacteria (100)	Firmicutes (100)	Bacilli (100)	Bacillales (100)	Planococcaceae (98)	Unclassified (<80)
OTU1286	Bacteria (100)	Proteobacteria (100)	Gammaproteobacteria (100)	Pasteurellales (100)	Pasteurellaceae (100)	*Haemophilus (81)*
OTU1287	Bacteria (100)	Firmicutes (100)	Bacilli (100)	Lactobacillales (100)	Streptococcaceae (100)	*Streptococcus (100)*
OTU1288	Bacteria (100)	Firmicutes (100)	Bacilli (100)	Lactobacillales (100)	Streptococcaceae (100)	*Streptococcus (100)*
OTU1289	Bacteria (100)	Proteobacteria (100)	Gammaproteobacteria (100)	Enterobacteriales (100)	Enterobacteriaceae (100)	*Escherichia/Shigella (82)*
OTU1290	Bacteria (100)	Firmicutes (100)	Bacilli (100)	Bacillales (97)	Unclassified (<80)	Unclassified (<80)
OTU1291	Bacteria (100)	Proteobacteria (100)	Gammaproteobacteria (100)	Pasteurellales (100)	Pasteurellaceae (100)	*Haemophilus (89)*

#OTU	DOMAIN (%)	PHYLUM (%)	CLASS/SUBCLASS (%)	ORDER (%)	FAMILY (%)	*GENUS (%)*
OTU1306	Bacteria (100)	Proteobacteria (100)	Betaproteobacteria (100)	Burkholderiales (100)	Burkholderiaceae (100)	*Burkholderia (100)*
OTU1307	Bacteria (100)	Proteobacteria (100)	Unclassified (<80)	Unclassified (<80)	Unclassified (<80)	Unclassified (<80)
OTU1308	Bacteria (100)	Proteobacteria (100)	Alphaproteobacteria (100)	Rhizobiales (100)	Rhizobiaceae (97)	Unclassified (<80)
OTU1309	Bacteria (100)	Actinobacteria (100)	Actinobacteria (100)	Streptomycetales (100)	Streptomycetaceae (100)	*Streptomyces (95)*
OTU1310	Bacteria (100)	Firmicutes (100)	Bacilli (95)	Bacillales (86)	Unclassified (<80)	Unclassified (<80)
OTU1311	Bacteria (100)	Firmicutes (100)	Bacilli (100)	Bacillales (100)	Bacillaceae 2 (100)	*Virgibacillus (90)*
OTU1312	Bacteria (100)	Proteobacteria (100)	Gammaproteobacteria (100)	Pseudomonadales (100)	Moraxellaceae (100)	*Acinetobacter (98)*
OTU1313	Bacteria (100)	Firmicutes (100)	Bacilli (100)	Bacillales (99)	Bacillaceae 2 (89)	Unclassified (<80)
OTU1314	Bacteria (100)	Firmicutes (100)	Bacilli (92)	Bacillales (86)	Unclassified (<80)	Unclassified (<80)
OTU1315	Bacteria (100)	Proteobacteria (100)	Gammaproteobacteria (100)	Alteromonadales (99)	Idiomarinaceae (95)	*Aliidiomarina (91)*
OTU1316	Bacteria (100)	Actinobacteria (100)	Actinobacteria (100)	Micrococcales (100)	Micrococcaceae (100)	*Arthrobacter (100)*
OTU1317	Bacteria (100)	Chloroflexi (98)	Anaerolineae (95)	Anaerolineales (95)	Anaerolineaceae (95)	Unclassified (<80)
OTU1318	Bacteria (100)	Proteobacteria (100)	Alphaproteobacteria (100)	Sphingomonadales (100)	Sphingomonadaceae (100)	Unclassified (<80)
OTU1319	Bacteria (100)	Actinobacteria (100)	Actinobacteria (100)	Propionibacteriales (100)	Propionibacteriaceae (100)	*Propionibacterium (100)*
OTU1320	Bacteria (100)	Proteobacteria (100)	Gammaproteobacteria (100)	Enterobacteriales (99)	Enterobacteriaceae (99)	Unclassified (<80)
OTU1321	Bacteria (100)	Firmicutes (100)	Bacilli (100)	Bacillales (100)	Planococcaceae (96)	*Sporosarcina (85)*
OTU1322	Bacteria (100)	Firmicutes (100)	Bacilli (100)	Bacillales (100)	Planococcaceae (91)	Unclassified (<80)
OTU1323	Bacteria (100)	Proteobacteria (100)	Gammaproteobacteria (100)	Pseudomonadales (100)	Moraxellaceae (100)	*Acinetobacter (100)*
OTU1324	Bacteria (100)	Proteobacteria (100)	Gammaproteobacteria (100)	Enterobacteriales (100)	Enterobacteriaceae (100)	Unclassified (<80)
OTU1325	Bacteria (100)	Firmicutes (100)	Clostridia (100)	Clostridiales (100)	Clostridiaceae 1 (97)	*Clostridium sensu str (97)*
OTU1326	Bacteria (100)	Proteobacteria (100)	Betaproteobacteria (100)	Burkholderiales (100)	Burkholderiaceae (100)	*Burkholderia (100)*
OTU1327	Bacteria (100)	Firmicutes (100)	Bacilli (100)	Bacillales (100)	Bacillales incertae sedis XII (92)	*Exiguobacterium (92)*
OTU1328	Bacteria (100)	Proteobacteria (100)	Betaproteobacteria (100)	Burkholderiales (100)	Comamonadaceae (100)	*Comamonas (94)*
OTU1329	Bacteria (100)	Firmicutes (100)	Bacilli (100)	Bacillales (100)	Planococcaceae (97)	*Planomicrobium (84)*
OTU1330	Bacteria (100)	Proteobacteria (100)	Gammaproteobacteria (100)	Enterobacteriales (100)	Enterobacteriaceae (100)	Unclassified (<80)
OTU1331	Bacteria (100)	Proteobacteria (100)	Gammaproteobacteria (100)	Enterobacteriales (100)	Enterobacteriaceae (100)	Unclassified (<80)
OTU1332	Bacteria (100)	Firmicutes (100)	Bacilli (100)	Lactobacillales (100)	Enterococcaceae (100)	*Enterococcus (100)*
OTU1333	Bacteria (100)	Bacteroidetes (100)	Bacteroidia (100)	Bacteroidales (100)	Prevotellaceae (100)	*Prevotella (100)*
OTU1334	Bacteria (100)	Firmicutes (100)	Negativicutes (100)	Selenomonadales (100)	Veillonellaceae (100)	*Veillonella (100)*
OTU1335	Bacteria (100)	Verrucomicrobia (100)	Spartobacteria (100)	Spartobacteria genera incertae sedis (98)	--------	--------
OTU1336	Bacteria (100)	Firmicutes (100)	Clostridia (100)	Clostridiales (100)	Lachnospiraceae (100)	Unclassified (<80)
OTU1337	Bacteria (100)	Firmicutes (100)	Bacilli (100)	Bacillales (100)	Bacillaceae 1 (95)	*Bacillus (87)*
OTU1338	Bacteria (100)	Proteobacteria (100)	Gammaproteobacteria (100)	Enterobacteriales (100)	Enterobacteriaceae (100)	Unclassified (<80)

#OTU	DOMAIN (%)	PHYLUM (%)	CLASS/SUBCLASS (%)	ORDER (%)	FAMILY (%)	*GENUS (%)*
OTU1352	Bacteria (100)	Actinobacteria (100)	Actinobacteria (100)	Micrococcales (100)	Micrococcaceae (100)	Unclassified (<80)
OTU1353	Bacteria (100)	Firmicutes (100)	Bacilli (100)	Bacillales (98)	Bacillaceae 2 (98)	*Halolactibacillus (87)*
OTU1354	Bacteria (100)	Firmicutes (100)	Bacilli (100)	Bacillales (99)	Unclassified (<80)	Unclassified (<80)
OTU1355	Bacteria (100)	Actinobacteria (100)	Actinobacteria (100)	Propionibacteriales (100)	Nocardioidaceae (100)	*Nocardioides (100)*
OTU1356	Bacteria (100)	Actinobacteria (100)	Actinobacteria (100)	Pseudonocardiales (100)	Pseudonocardiaceae (99)	Unclassified (<80)
OTU1357	Bacteria (100)	Proteobacteria (100)	Gammaproteobacteria (100)	Pseudomonadales (100)	Pseudomonadaceae (100)	Unclassified (<80)
OTU1358	Bacteria (100)	Actinobacteria (100)	Actinobacteria (100)	Streptomycetales (100)	Streptomycetaceae (83)	Unclassified (<80)
OTU1359	Bacteria (100)	Bacteroidetes (100)	Bacteroidia (100)	Bacteroidales (100)	Porphyromonadaceae (97)	Unclassified (<80)
OTU1360	Bacteria (100)	Firmicutes (100)	Bacilli (100)	Bacillales (90)	Bacillaceae 1 (80)	Unclassified (<80)
OTU1361	Bacteria (100)	Proteobacteria (100)	Gammaproteobacteria (100)	Enterobacteriales (100)	Enterobacteriaceae (100)	Unclassified (<80)
OTU1362	Bacteria (100)	Firmicutes (100)	Bacilli (100)	Bacillales (100)	Bacillales incertae sedis XII (99)	*Exiguobacterium (99)*
OTU1363	Bacteria (100)	Firmicutes (100)	Bacilli (100)	Bacillales (100)	Unclassified (<80)	Unclassified (<80)
OTU1364	Bacteria (99)	Firmicutes (95)	Bacilli (89)	Bacillales (89)	Unclassified (<80)	Unclassified (<80)
OTU1365	Bacteria (100)	Proteobacteria (100)	Gammaproteobacteria (100)	Enterobacteriales (98)	Enterobacteriaceae (98)	Unclassified (<80)
OTU1366	Bacteria (100)	Proteobacteria (100)	Alphaproteobacteria (100)	Sphingomonadales (100)	Sphingomonadaceae (100)	*Sphingopyxis (100)*
OTU1367	Bacteria (100)	Firmicutes (100)	Bacilli (100)	Bacillales (100)	Bacillaceae 1 (85)	*Bacillus (83)*
OTU1368	Bacteria (100)	Firmicutes (100)	Bacilli (100)	Bacillales (100)	Bacillaceae 1 (97)	*Anoxybacillus (96)*
OTU1369	Bacteria (100)	Firmicutes (100)	Bacilli (100)	Bacillales (100)	Planococcaceae (89)	Unclassified (<80)
OTU1370	Bacteria (100)	Proteobacteria (100)	Alphaproteobacteria (100)	Rhizobiales (100)	Rhizobiaceae (100)	Unclassified (<80)
OTU1371	Bacteria (100)	Cyanobact./Chloroplast (100)	-------	-------	-------	-------
OTU1372	Bacteria (100)	Proteobacteria (100)	Alphaproteobacteria (100)	Rhizobiales (100)	Bradyrhizobiaceae (99)	*Bradyrhizobium (86)*
OTU1373	Bacteria (100)	Proteobacteria (100)	Betaproteobacteria (100)	Burkholderiales (100)	Burkholderiaceae (90)	*Limnobacter (85)*
OTU1374	Bacteria (100)	Firmicutes (100)	Bacilli (100)	Bacillales (97)	Bacillaceae 1 (87)	*Bacillus (86)*
OTU1375	Bacteria (100)	Actinobacteria (100)	Actinobacteria (100)	Micrococcales (100)	Cellulomonadaceae (83)	*Cellulomonas (80)*
OTU1376	Bacteria (100)	Firmicutes (100)	Bacilli (100)	Lactobacillales (100)	Enterococcaceae (100)	*Enterococcus (99)*
OTU1377	Bacteria (100)	Actinobacteria (100)	Actinobacteria (100)	Pseudonocardiales (100)	Unclassified (<80)	Unclassified (<80)
OTU1378	Bacteria (100)	Proteobacteria (100)	Alphaproteobacteria (100)	Rhizobiales (100)	Bradyrhizobiaceae (100)	*Bradyrhizobium (100)*
OTU1379	Bacteria (100)	Firmicutes (100)	Bacilli (100)	Lactobacillales (98)	Enterococcaceae (98)	Unclassified (<80)
OTU1380	Bacteria (100)	Proteobacteria (100)	Gammaproteobacteria (100)	Xanthomonadales (100)	Xanthomonadaceae (100)	*Stenotrophomonas (94)*
OTU1381	Bacteria (100)	Proteobacteria (100)	Betaproteobacteria (100)	Burkholderiales (100)	Comamonadaceae (96)	Unclassified (<80)
OTU1382	Bacteria (100)	Firmicutes (100)	Clostridia (100)	Clostridiales (100)	Lachnospiraceae (99)	*Clostridium (83) XlVa*
OTU1383	Bacteria (100)	Firmicutes (100)	Clostridia (100)	Clostridiales (100)	Clostridiaceae 1 (100)	*Clostridium sensu st*[illegible] *(100)*
OTU1384	Bacteria (100)	Firmicutes (100)	Bacilli (100)	Bacillales (100)	Planococcaceae (97)	*Planomicrobium (80)*
OTU1385	Bacteria (100)	Proteobacteria (100)	Alphaproteobacteria (100)	Sphingomonadales (100)	Sphingomonadaceae (100)	*Sphingomonas (100)*
[illegible]	Bacteria [illegible]	Proteobacteria [illegible]	Gammaproteobacteria [illegible]	Enterobacteriales [illegible]	Enterobacteriaceae [illegible]	Unclassified [illegible]

#OTU	DOMAIN (%)	PHYLUM (%)	CLASS/SUBCLASS (%)	ORDER (%)	FAMILY (%)	*GENUS (%)*
**OTU1399**	**Bacteria (100)**	**Actinobacteria (100)**	**Actinobacteria (100)**	**Pseudonocardiales (100)**	**Pseudonocardiaceae (100)**	***Pseudonocardia (96)***
**OTU1400**	**Bacteria (100)**	**Bacteroidetes (100)**	**Sphingobacteriia (100)**	**Sphingobacteriales (100)**	**Sphingobacteriaceae (100)**	***Sphingobacterium (100)***
**OTU1401**	**Bacteria (100)**	**Proteobacteria (100)**	**Gammaproteobacteria (100)**	**Aeromonadales (100)**	**Aeromonadaceae (100)**	***Aeromonas (100)***
**OTU1402**	**Bacteria (100)**	**Actinobacteria (100)**	**Actinobacteria (100)**	**Micrococcales (100)**	**Microbacteriaceae (100)**	***Herbiconiux (87)***

† = taxonomic rank not yet been defined
