# Supplementary material for: Three ancient documents solve the jigsaw of the parchment purple spot deterioration and validate the microbial succession model
Source: Sci Rep. 2019 Feb 7;9:1623. doi: 10.1038/s41598-018-37651-y (PMC6367363; doi:10.1038/s41598-018-37651-y)
Supplement: Supplementary file 3 — Number of sequences found in each replicate sample from the three parchments [file 41598_2018_37651_MOESM3_ESM.pdf]

Three ancient documents solve the jigsaw of the parchment purple spot deterioration and validate the microbial succession model

Migliore L., PeriniN., Mercuri F., Orlanducci S., Rubechini A. & Thaller M.C.

Table S2. Number of sequences found in each replicate sample from the three parchments

#OTU	AP1	AP2	AP3	BP1	BP2	BP3	CP1	CP2	CP3	AU1	AU2	AU3	BU1	BU2	BU3	CU1	CU2	CU3
**OTU0001**	0	1	0	0	0	0	0	0	0	0	0	0	0	0	0	0	0	0
**OTU0002**	0	0	0	0	0	0	0	0	0	1	0	0	0	0	5	0	0	0
**OTU0003**	0	1	0	0	0	0	0	0	0	1	0	0	0	0	0	0	0	0
**OTU0004**	0	0	0	0	0	0	0	0	0	1	0	0	0	0	0	1	0	0
**OTU0005**	8	19	16	2	3	0	4	9	1	5	22	1	2	3	4	6	4	4
**OTU0006**	57	679	185	5	3	2	8	4	2	11	229	10	2	9	2	17	6	20
**OTU0007**	0	0	3	0	1	0	0	1	0	4	4	0	0	2	0	0	0	0
**OTU0008**	2	0	0	0	0	0	0	0	1	0	1	0	0	1	0	0	0	0
**OTU0009**	1	0	0	0	0	0	1	0	0	0	0	0	0	0	0	0	0	0
**OTU0010**	0	0	1	0	1	0	1	0	1	0	0	1	0	0	0	0	0	1
**OTU0011**	0	6	1	2	1	1	0	0	0	1	1	1	0	0	0	0	1	2
**OTU0012**	0	1	0	1	0	0	0	0	0	0	0	0	0	0	0	0	0	0
**OTU0013**	0	1	1	0	0	0	0	0	0	0	0	0	1	0	1	1	0	0
**OTU0014**	1	0	3	0	0	0	0	0	0	0	0	0	0	0	0	0	1	0
**OTU0015**	13	12	7	5	5	4	9	7	4	17	28	2	3	9	1	3	7	6
**OTU0016**	0	0	0	0	0	0	0	1	0	1	0	1	0	0	0	0	0	0
**OTU0017**	56	84	93	22	33	16	59	30	11	101	93	62	19	41	8	26	48	30
**OTU0018**	1	4	0	1	0	0	1	0	1	1	2	0	0	0	0	0	0	0
**OTU0019**	11	26	45	15	19	6	19	9	6	22	52	18	4	17	5	6	21	17
**OTU0020**	2	1	3	1	1	0	1	1	0	4	0	0	0	0	0	1	1	1
**OTU0021**	0	0	1	0	0	0	0	0	0	0	1	0	0	0	0	0	0	0
**OTU0022**	0	0	0	0	0	0	0	2	0	0	5	0	0	0	0	0	1	5
**OTU0023**	0	1	0	1	0	0	0	0	0	0	0	0	0	0	0	0	0	0
**OTU0024**	0	2	0	0	0	1	0	0	0	0	0	0	0	1	0	0	0	0
**OTU0025**	0	0	0	0	0	0	0	0	0	0	0	0	0	0	0	1	0	0
**OTU0026**	0	0	0	1	0	0	0	0	0	0	0	0	0	0	0	1	0	0
**OTU0027**	0	0	2	0	0	0	0	0	0	0	0	0	1	0	0	0	0	0
**OTU0028**	3	7	62	0	0	1	1	3	3	16	37	1	72	46	53	2	4	0
**OTU0029**	0	0	0	0	0	0	0	0	0	0	0	0	0	1	0	0	0	0
**OTU0030**	9	9	36	0	6	1	0	2	0	51	25	17	5	1	1	0	0	0
**OTU0031**	0	0	1	0	0	0	0	0	1	3	1	0	0	0	0	1	0	1
**OTU0032**	4	16	36	1	2	5	29	3	6	9	12	10	4	37	3	13	3	0
**OTU0033**	1	0	0	0	0	0	1	1	1	0	2	1	1	1	0	1	0	0
**OTU0034**	0	6	15	11	1	1	2	0	1	10	9	4	2	5	2	25	2	0

**OTU0035**	3	2	0	1	0	0	1	2	1	0	4	1	0	0	2	0	0	0
**OTU0036**	2	4	2	0	1	0	0	0	0	1	0	0	0	0	17	0	0	3
**OTU0037**	0	0	1	0	0	0	0	0	0	0	2	0	0	0	4	0	0	0
**OTU0038**	0	0	0	0	0	0	0	1	0	0	0	0	0	0	0	0	0	0
**OTU0039**	0	1	0	0	0	0	0	0	0	0	0	0	0	0	0	0	0	0
**OTU0040**	2	7	6	1	2	0	1	0	0	5	4	2	0	1	0	0	2	1
**OTU0041**	4	5	3	0	2	0	6	0	2	2	3	1	1	3	1	5	1	6
**OTU0042**	1	1	1	1	0	0	0	0	0	0	0	0	0	1	0	1	0	0
**OTU0043**	0	0	0	0	0	0	0	0	0	0	1	0	0	0	0	0	1	0
**OTU0044**	2153	3136	3742	1173	1021	471	1817	1153	692	2821	4610	1808	558	1483	449	1291	1758	1485
**OTU0045**	5	10	496	0	2	2	2	4	7	476	348	3	31	64	24	2	0	1
**OTU0046**	16	28	36	4	11	0	10	5	4	16	30	9	2	3	245	7	5	9
**OTU0047**	2	1	0	0	0	0	0	0	0	0	1	0	0	0	0	0	0	0
**OTU0048**	1	0	0	0	0	0	0	0	0	0	0	0	0	0	0	1	0	0
**OTU0049**	0	1	1	0	0	0	0	0	0	1	1	0	0	0	2	0	0	0
**OTU0050**	3	2	1	0	0	0	0	0	0	2	6	3	0	0	0	4	1	3
**OTU0051**	1	5	1	1	1	0	2	0	1	4	2	1	0	0	0	2	0	1
**OTU0052**	0	1	1	0	0	0	1	0	0	3	1	0	0	0	0	0	0	0
**OTU0053**	1	0	10	0	0	0	0	0	0	0	0	0	0	0	0	0	0	0
**OTU0054**	0	0	1	0	0	0	0	0	0	1	0	0	0	0	0	0	0	0
**OTU0055**	0	0	0	0	0	1	0	0	1	0	0	0	0	0	0	0	0	0
**OTU0056**	0	2	0	0	0	0	0	0	0	0	0	0	0	0	0	0	0	0
**OTU0057**	10	6	1	0	0	0	0	0	0	5	4	0	0	0	0	0	0	0
**OTU0058**	0	0	0	0	0	0	3	0	2	0	0	4	1	0	0	0	7	8
**OTU0059**	0	0	0	0	0	0	0	0	0	0	0	0	0	0	0	0	1	1
**OTU0060**	0	1	2	0	0	0	0	1	0	0	0	0	0	0	0	0	0	0
**OTU0061**	6	8	6	0	1	0	21	1	1	2	3	5	0	1	1	0	0	1
**OTU0062**	7	19	22	12	7	2	12	5	3	22	27	6	2	12	1	5	12	15
**OTU0063**	1	1	0	0	0	0	0	0	0	0	0	0	0	0	0	0	0	0
**OTU0064**	0	1	2	0	0	0	4	0	0	0	0	0	0	2	0	1	0	0
**OTU0065**	1	0	1	1	0	0	1	0	1	1	1	0	0	0	0	0	2	1
**OTU0066**	0	1	0	0	0	0	0	0	0	0	1	0	0	0	0	1	0	0
**OTU0067**	0	0	0	0	0	0	1	0	0	0	1	0	0	0	0	0	0	0
**OTU0068**	1	0	0	0	0	0	0	0	0	1	0	0	0	0	0	0	0	0
**OTU0069**	0	0	0	0	0	0	0	0	1	0	3	0	0	0	0	0	0	0
**OTU0070**	1	0	0	0	0	0	2	0	0	1	0	0	0	0	0	0	0	0
**OTU0071**	3	5	8	1	4	2	5	3	1	10	9	20	0	5	0	6	4	4
**OTU0072**	0	2	3	1	1	2	0	0	0	3	1	1	1	0	1	1	0	1
**OTU0073**	0	0	0	0	1	0	1	0	0	0	0	0	0	0	0	0	0	0

**OTU0074**	0	2	2	0	0	0	0	0	0	0	0	0	0	0	0	0	0	0
**OTU0075**	0	0	0	0	0	0	0	0	1	0	3	0	0	0	0	0	0	0
**OTU0076**	1	9	12	2	1	0	1	1	0	8	7	5	3	0	6	2	1	2
**OTU0077**	18	33	33	3	10	3	21	10	6	9	30	16	3	11	5	24	12	23
**OTU0078**	0	0	3	0	0	0	0	0	0	1	0	0	0	0	0	0	0	1
**OTU0079**	1	0	1	0	0	0	3	0	0	0	0	1	0	1	0	0	0	0
**OTU0080**	1	0	0	0	0	0	1	0	1	0	1	1	0	0	0	1	0	0
**OTU0081**	27	92	74	40	22	5	36	15	21	110	87	23	12	29	15	49	27	39
**OTU0082**	1	1	3	0	0	0	0	0	0	9	4	2	0	1	0	0	1	3
**OTU0083**	2	0	0	0	2	0	1	0	0	0	2	1	0	3	2	0	1	0
**OTU0084**	5	9	39	1	1	1	3	3	4	12	6	10	0	1	0	6	4	4
**OTU0085**	0	1	0	0	0	0	0	0	0	0	1	1	0	0	0	0	0	0
**OTU0086**	0	6	2	0	0	0	2	0	0	2	0	0	0	2	0	1	0	0
**OTU0087**	1	3	0	0	2	0	2	0	0	4	1	2	0	0	0	0	5	0
**OTU0088**	0	0	0	1	0	0	0	0	0	0	1	0	0	0	0	0	0	0
**OTU0089**	0	0	1	0	0	0	0	0	0	1	0	0	1	0	0	0	0	0
**OTU0090**	0	0	4	1	0	0	1	0	0	1	1	1	0	1	0	2	2	1
**OTU0091**	5	0	0	0	0	0	0	0	1	0	3	0	0	0	0	2	0	0
**OTU0092**	39	66	61	15	26	10	33	13	14	56	67	31	12	25	380	21	19	23
**OTU0093**	1	1	4	0	2	0	3	0	0	1	2	0	1	1	0	1	1	1
**OTU0094**	0	0	0	0	0	0	0	0	0	1	0	0	0	0	0	0	0	0
**OTU0095**	1439	2610	3713	248	342	111	656	524	183	3276	4530	1177	133	370	90	746	971	737
**OTU0096**	0	0	0	1	0	0	9	0	0	0	0	0	0	0	0	0	0	0
**OTU0097**	0	2	0	0	0	0	0	1	0	0	0	0	0	0	2	0	0	0
**OTU0098**	0	1	0	0	0	0	0	0	0	0	1	0	0	0	0	0	0	0
**OTU0099**	1	0	1	3	0	0	2	0	0	1	0	0	0	2	0	1	0	2
**OTU0100**	43	79	68	34	26	18	54	37	16	58	101	40	10	33	11	35	45	33
**OTU0101**	0	0	0	0	0	0	0	1	0	0	0	1	0	1	0	0	0	0
**OTU0102**	1	0	0	0	0	0	0	0	0	2	0	0	0	0	0	3	0	0
**OTU0103**	1	0	0	0	0	0	0	1	0	0	0	0	0	0	0	0	0	0
**OTU0104**	0	0	1	0	0	0	0	0	0	1	0	0	0	0	0	0	0	0
**OTU0105**	9	22	30	5	2	2	15	4	0	4	30	8	4	3	4	1	0	4
**OTU0106**	9	7	6	1	1	1	19	2	2	4	3	6	0	1	0	0	0	2
**OTU0107**	1	0	0	0	0	0	0	0	0	3	2	3	0	1	1	2	0	0
**OTU0108**	1	0	0	0	0	0	1	0	0	0	0	0	0	0	0	1	0	0
**OTU0109**	2	2	6	0	1	0	4	1	0	0	174	1	0	3	1	0	1	2
**OTU0110**	1	0	0	0	0	0	0	0	0	0	0	0	0	0	0	0	0	0
**OTU0111**	41	107	177	14	27	27	100	22	7	57	134	60	32	38	10	9	29	21
**OTU0112**	1	0	0	0	0	0	214	0	0	2	0	0	0	0	0	0	0	1

**OTU0113**	3	2	12	0	0	0	0	4	0	1	3	0	0	0	0	2	15	4
**OTU0114**	1	0	0	0	0	0	0	1	0	0	0	0	0	0	0	0	0	0
**OTU0115**	2	0	0	0	0	0	1	0	1	0	0	0	0	1	0	0	0	0
**OTU0116**	0	1	1	0	0	0	0	1	0	0	0	2	0	0	0	2	0	0
**OTU0117**	1	0	1	0	0	0	0	1	0	2	0	3	0	0	0	13	4	4
**OTU0118**	0	0	0	0	0	0	0	0	0	0	1	0	1	0	0	1	0	1
**OTU0119**	0	0	0	1	0	0	0	0	0	0	1	1	0	0	0	0	0	0
**OTU0120**	0	0	6	0	0	1	1	0	0	5	7	0	0	2	0	0	0	0
**OTU0121**	1	0	0	0	0	0	0	0	0	1	0	0	0	0	0	0	0	0
**OTU0122**	0	1	0	0	0	0	0	0	0	1	0	2	0	1	0	0	0	0
**OTU0123**	0	0	0	0	0	0	0	0	0	0	1	2	0	0	0	1	0	1
**OTU0124**	182	243	360	56	37	27	147	75	41	232	328	158	29	64	17	107	135	124
**OTU0125**	0	0	0	0	0	0	0	0	0	1	2	0	0	0	1	0	0	0
**OTU0126**	1	0	24	0	0	0	0	0	0	0	1	0	0	0	0	0	0	0
**OTU0127**	4	1	0	1	0	0	3	3	0	1	3	1	1	3	0	1	0	0
**OTU0128**	0	0	0	0	2	0	0	0	0	3	0	0	0	0	0	0	0	0
**OTU0129**	0	0	0	0	0	0	1	0	0	0	0	2	0	0	0	0	0	0
**OTU0130**	0	0	0	0	0	0	0	0	0	0	0	0	0	0	0	0	1	1
**OTU0131**	0	0	0	0	0	0	1	0	0	1	1	0	0	0	0	0	0	0
**OTU0132**	28	31	31	1	11	8	20	20	8	10	30	25	6	4	7	2	3	0
**OTU0133**	0	0	1	0	0	0	0	0	0	0	0	0	0	0	0	0	0	0
**OTU0134**	185	133	74	12	7	2	629	11	14	54	91	85	0	23	81	21	21	26
**OTU0135**	40	61	60	25	9	12	43	15	9	51	80	32	13	30	11	17	29	22
**OTU0136**	0	3	0	0	0	0	0	0	0	0	0	0	1	0	0	0	0	0
**OTU0137**	4	7	6	0	2	0	1	1	0	3	9	2	3	2	1	1	1	1
**OTU0138**	1	0	8	2	1	1	6	0	5	0	1	2	2	0	2	2	7	15
**OTU0139**	6	86	17	0	0	0	0	1	1	10	9	1	0	0	0	6	0	1
**OTU0140**	0	0	0	0	0	0	0	1	0	1	0	0	0	0	0	1	0	0
**OTU0141**	14	53	60	4	2	7	20	13	5	53	104	17	4	12	6	22	81	49
**OTU0142**	0	0	0	1	0	0	2	0	0	0	0	0	0	0	0	0	0	0
**OTU0143**	0	6	1	0	0	0	0	0	0	0	0	0	0	0	0	0	0	1
**OTU0144**	0	3	1	2	1	0	2	0	0	1	4	0	1	0	1	1	2	1
**OTU0145**	0	0	0	0	0	0	0	0	0	0	0	2	0	0	0	0	0	1
**OTU0146**	0	2	3	1	2	0	0	0	0	2	2	0	0	1	0	1	2	0
**OTU0147**	8	14	8	2	1	1	1	1	0	5	6	4	1	2	0	1	5	3
**OTU0148**	0	14	0	0	0	0	0	1	0	0	4	0	0	0	0	0	0	0
**OTU0149**	1	4	5	0	0	1	0	1	0	1	10	5	0	0	90	1	0	0
**OTU0150**	2	5	10	1	0	1	4	0	4	3	9	2	1	3	0	1	2	2
**OTU0151**	1	3	1	1	0	1	4	1	0	4	3	3	1	2	0	5	1	1

**OTU0152**	2	3	1	0	0	0	0	0	0	0	3	1	0	0	0	7	0	4
**OTU0153**	0	2	12	1	1	0	7	4	1	9	4	0	0	1	0	3	2	0
**OTU0154**	3	3	4	0	1	0	2	0	1	2	8	1	0	0	33	0	0	2
**OTU0155**	2	4	9	5	1	2	7	0	0	9	4	0	0	0	0	1	1	0
**OTU0156**	3	5	4	0	0	0	0	0	0	0	0	0	1	0	0	3	5	2
**OTU0157**	1	0	1	0	0	0	3	0	0	0	1	0	0	0	0	0	0	0
**OTU0158**	1	0	2	0	0	0	0	0	0	2	1	1	0	0	0	0	0	0
**OTU0159**	0	0	0	0	0	0	0	0	0	0	0	1	0	0	0	2	0	2
**OTU0160**	0	0	0	0	0	0	0	1	0	0	0	0	1	0	2	1	0	0
**OTU0161**	1	7	3	1	3	3	2	1	1	3	7	1	3	2	0	2	1	2
**OTU0162**	0	0	1	0	0	0	0	1	0	0	0	0	0	0	0	0	0	0
**OTU0163**	1	46	20	0	0	0	0	1	0	7	0	0	0	0	0	0	0	0
**OTU0164**	0	0	0	0	0	0	0	1	0	0	0	0	0	0	0	0	7	1
**OTU0165**	6	7	6	4	7	2	11	3	1	5	9	5	8	7	1	2	6	0
**OTU0166**	0	0	1	0	0	0	2	2	0	0	0	0	0	0	0	0	0	0
**OTU0167**	0	6	1	1	1	0	0	0	1	0	1	1	3	0	2	1	0	3
**OTU0168**	0	0	0	1	1	0	0	0	0	1	1	2	0	0	33	0	1	0
**OTU0169**	0	0	4	0	0	0	1	0	0	0	2	0	0	1	0	2	0	2
**OTU0170**	0	1	1	0	0	0	0	0	0	0	0	0	0	0	0	0	0	1
**OTU0171**	1	1	3	0	0	0	2	1	2	2	4	1	1	1	2	1	1	2
**OTU0172**	1	0	0	0	0	0	1	0	0	2	1	0	0	0	0	1	1	0
**OTU0173**	0	0	0	0	0	0	0	2	0	0	0	0	0	0	1	0	0	0
**OTU0174**	9	21	43	14	5	1	15	7	5	33	27	12	3	11	2	19	13	17
**OTU0175**	0	0	0	0	0	1	0	0	0	0	0	0	0	1	0	1	1	1
**OTU0176**	0	1	0	0	0	0	0	1	0	0	1	1	0	0	0	0	0	0
**OTU0177**	1	2	2	0	0	0	0	0	0	4	6	4	0	0	0	10	1	5
**OTU0178**	0	0	3	0	0	0	12	26	4	1	2	0	0	3	0	2	0	0
**OTU0179**	0	0	0	0	0	0	0	0	0	0	0	0	1	0	3	0	0	0
**OTU0180**	138	1311	254	10	10	7	105	215	28	51	411	31	8	38	7	37	41	35
**OTU0181**	0	1	0	0	0	0	0	0	1	0	0	0	0	0	0	1	0	0
**OTU0182**	0	1	0	0	0	0	0	0	0	2	0	0	0	0	0	0	0	0
**OTU0183**	0	2	0	2	0	0	0	2	0	1	3	0	0	0	0	0	0	0
**OTU0184**	5	8	10	3	5	2	5	6	0	15	16	6	2	4	0	7	1	1
**OTU0185**	0	0	0	0	0	0	1	0	0	0	0	2	0	0	0	0	0	0
**OTU0186**	0	1	0	0	0	0	0	0	1	0	1	1	0	0	34	1	1	0
**OTU0187**	2	13	3	0	0	0	0	0	0	0	0	0	0	0	0	0	0	0
**OTU0188**	1	3	6	1	1	1	8	0	2	4	8	1	1	2	1	6	3	3
**OTU0189**	2	9	7	0	0	0	0	0	0	6	5	5	0	2	0	4	3	2
**OTU0190**	3	6	3	1	1	1	0	3	3	4	6	4	1	1	1	2	5	4

**OTU0191**	5	15	19	9	6	1	10	3	1	7	12	7	6	12	8	7	6	2
**OTU0192**	0	0	0	0	0	0	0	0	0	0	0	0	0	0	0	2	0	0
**OTU0193**	0	0	1	0	1	1	0	5	0	0	1	2	0	4	0	0	1	0
**OTU0194**	0	0	0	0	0	0	1	1	0	0	0	0	0	0	0	0	0	0
**OTU0195**	0	1	0	0	0	0	0	0	0	0	1	0	0	0	0	0	0	0
**OTU0196**	13	20	14	7	2	1	5	5	2	13	5	3	2	10	2	7	9	6
**OTU0197**	8	16	14	3	3	0	13	3	3	4	15	9	3	6	3	0	1	0
**OTU0198**	0	2	0	0	0	0	0	1	0	0	2	1	0	1	1	0	0	0
**OTU0199**	0	1	0	0	0	0	0	0	0	0	0	0	0	0	0	0	0	0
**OTU0200**	31	52	32	6	9	8	16	13	6	21	20	26	7	7	4	4	8	6
**OTU0201**	1	1	1	0	0	0	0	0	0	1	0	0	1	0	0	0	0	0
**OTU0202**	0	0	1	0	0	0	1	0	0	0	1	0	0	0	0	0	2	0
**OTU0203**	1	0	0	0	0	1	0	0	0	1	1	0	0	0	0	1	0	0
**OTU0204**	0	1	0	0	0	0	0	0	0	0	0	0	0	0	0	0	0	2
**OTU0205**	2	6	1	0	0	0	0	0	0	0	12	1	0	0	0	3	3	0
**OTU0206**	0	0	0	0	1	0	0	0	0	0	0	0	0	0	0	0	0	4
**OTU0207**	0	1	0	0	1	0	0	0	0	0	0	0	0	0	0	0	0	0
**OTU0208**	0	0	0	0	0	0	0	1	0	0	0	1	0	0	0	0	0	0
**OTU0209**	0	0	0	0	0	0	0	0	3	0	0	0	0	0	0	0	3	0
**OTU0210**	1	8	0	2	1	0	3	0	2	1	11	1	0	1	0	4	0	1
**OTU0211**	0	0	0	1	0	0	0	0	0	2	0	0	0	0	0	1	0	0
**OTU0212**	0	1	0	0	1	0	0	0	0	0	0	0	0	0	0	0	0	0
**OTU0213**	0	0	1	0	0	0	0	0	0	0	0	0	0	0	1	0	0	1
**OTU0214**	64	125	170	90	62	15	102	79	75	235	238	76	27	123	21	109	147	78
**OTU0215**	0	1	0	0	0	0	0	0	0	2	0	2	0	0	1	0	0	0
**OTU0216**	4	30	12	0	1	0	3	0	2	5	57	1	0	0	0	4	0	1
**OTU0217**	4	6	0	0	0	0	0	0	1	0	9	5	1	6	0	0	0	1
**OTU0218**	0	0	0	0	0	0	3	0	0	0	0	0	0	2	0	1	0	0
**OTU0219**	0	0	1	0	0	0	1	0	0	1	1	0	0	0	1	0	0	0
**OTU0220**	47	41	29	8	16	6	29	22	9	16	35	32	16	6	6	5	6	6
**OTU0221**	0	1	0	0	0	0	0	0	0	0	2	0	0	0	0	2	0	0
**OTU0222**	0	1	0	1	1	0	1	0	0	6	6	0	0	1	0	1	1	0
**OTU0223**	0	0	0	0	0	0	0	0	0	0	0	0	0	0	0	1	0	1
**OTU0224**	7	29	14	3	6	1	7	3	6	9	9	3	3	14	0	12	10	16
**OTU0225**	0	0	15	0	0	0	2	0	0	2	1	0	0	0	0	0	0	0
**OTU0226**	0	0	0	0	0	0	3	0	0	0	0	0	0	0	0	0	0	0
**OTU0227**	2	31	5	0	0	0	0	0	0	0	3	0	0	0	0	0	0	0
**OTU0228**	11	6	35	5	2	0	9	10	1	29	21	4	5	3	1	11	19	2
**OTU0229**	1	3	5	0	1	0	2	0	1	7	8	0	1	6	1	14	5	1

**OTU0230**	0	0	0	0	0	0	0	0	0	1	0	1	0	0	0	0	0	0
**OTU0231**	1	1	3	0	0	0	0	0	0	1	2	1	0	0	0	0	1	0
**OTU0232**	0	3	3	1	0	0	3	1	1	1	1	0	1	5	0	8	6	3
**OTU0233**	0	0	1	0	1	0	0	4	0	0	0	0	0	0	0	0	0	0
**OTU0234**	0	1	0	0	0	0	0	0	0	0	3	3	0	0	0	0	0	0
**OTU0235**	4	6	5	2	2	1	5	2	3	6	10	2	1	3	0	3	4	3
**OTU0236**	0	0	2	0	0	0	0	0	0	0	0	0	0	0	0	0	0	0
**OTU0237**	0	2	0	0	1	0	0	0	1	0	4	1	0	0	0	0	0	0
**OTU0238**	4	7	4	3	3	2	3	3	0	3	6	5	3	1	1	0	4	2
**OTU0239**	0	9	0	0	0	0	1	0	0	5	5	0	0	0	0	0	0	0
**OTU0240**	1	0	0	0	0	0	0	0	0	0	1	0	0	0	0	0	0	0
**OTU0241**	0	0	0	0	0	0	0	0	0	0	1	1	0	0	1	1	1	2
**OTU0242**	2	5	1	0	0	0	1	0	0	0	2	0	0	2	1	1	0	0
**OTU0243**	1	5	1	0	0	0	3	2	0	4	18	2	0	1	6	1	1	4
**OTU0244**	58	215	91	13	10	9	30	10	9	70	107	27	9	22	3	31	30	11
**OTU0245**	0	0	0	0	0	0	0	0	2	0	1	0	0	0	0	0	0	0
**OTU0246**	6	17	11	2	1	2	6	8	1	9	15	8	1	2	1	3	0	2
**OTU0247**	0	0	2	0	1	0	0	0	0	0	0	0	0	2	0	0	0	0
**OTU0248**	1	1	0	0	0	0	0	0	0	0	0	0	0	0	0	0	0	0
**OTU0249**	0	0	0	0	0	0	0	1	0	0	2	1	0	0	0	1	0	0
**OTU0250**	0	0	1	0	0	0	0	0	0	1	1	1	0	1	0	0	0	0
**OTU0251**	5	38	7	0	0	0	0	0	0	0	0	0	0	0	0	0	0	0
**OTU0252**	1	6	1	0	0	0	2	0	0	1	0	1	0	1	0	0	0	0
**OTU0253**	5	1	1	0	0	1	284	1	0	0	0	3	6	0	1	1	0	6
**OTU0254**	0	0	1	0	0	0	0	1	0	2	1	0	0	0	2	0	0	0
**OTU0255**	0	0	0	0	0	0	1	0	0	0	0	1	0	0	0	0	0	0
**OTU0256**	0	0	1	0	0	0	0	0	0	0	0	0	0	0	0	0	0	1
**OTU0257**	0	0	1	0	0	0	0	0	0	0	0	0	0	0	0	1	0	0
**OTU0258**	0	4	2	0	0	0	0	0	0	0	0	1	0	0	0	0	0	0
**OTU0259**	0	0	0	0	0	0	1	0	0	0	1	0	0	0	0	0	0	0
**OTU0260**	5	0	1	0	0	0	0	0	0	1	0	0	0	1	1	0	0	0
**OTU0261**	0	2	8	0	0	0	0	0	0	1	1	1	0	0	0	0	3	3
**OTU0262**	0	2	0	0	0	0	0	0	0	0	3	0	0	0	0	0	2	0
**OTU0263**	0	2	0	0	0	0	0	0	0	1	0	0	0	0	0	0	0	0
**OTU0264**	0	1	3	1	0	0	0	0	0	0	1	1	0	3	2	0	1	0
**OTU0265**	12	26	19	11	5	3	15	3	4	31	31	12	1	11	6	20	18	6
**OTU0266**	0	1	2	0	0	0	0	1	0	0	2	0	0	0	0	0	0	0
**OTU0267**	0	0	0	0	0	0	0	0	0	0	0	0	0	0	0	3	0	1
**OTU0268**	0	0	0	0	0	0	0	1	0	0	3	0	0	0	0	0	0	0

**OTU0269**	0	0	2	0	1	0	0	0	0	0	1	0	0	1	0	0	1	0
**OTU0270**	0	0	1	1	1	0	3	0	0	0	0	1	0	0	0	0	0	0
**OTU0271**	0	0	1	1	0	1	1	1	1	0	1	1	0	0	1	8	89	42
**OTU0272**	1	1	1	0	3	1	0	0	0	2	5	1	1	1	1	2	0	1
**OTU0273**	2	0	1	0	0	0	1	0	0	1	0	0	0	0	0	0	0	0
**OTU0274**	0	1	0	0	0	0	0	0	0	0	1	0	0	0	0	0	0	0
**OTU0275**	4	43	18	0	0	0	1	0	2	2	8	4	1	2	0	1	0	2
**OTU0276**	0	0	0	0	0	0	1	0	0	0	0	0	0	0	0	0	0	1
**OTU0277**	0	1	0	0	0	0	1	0	0	0	0	0	0	0	0	0	0	0
**OTU0278**	3	1	0	1	2	1	3	2	1	16	16	1	1	1	19	6	0	0
**OTU0279**	0	0	0	0	0	1	0	1	4	3	2	0	0	0	0	0	0	0
**OTU0280**	0	0	2	0	0	0	0	1	1	0	0	0	0	0	0	0	0	0
**OTU0281**	0	0	0	0	0	0	0	0	0	3	0	3	0	0	0	0	0	0
**OTU0282**	2	0	0	0	0	0	2	0	0	0	0	0	0	1	0	0	0	1
**OTU0283**	3	3	3	1	0	0	3	2	0	2	2	1	1	1	0	0	3	0
**OTU0284**	0	0	0	0	0	0	0	0	0	2	0	0	0	0	0	0	0	0
**OTU0285**	0	1	6	2	1	1	0	1	0	5	19	6	1	0	19	0	0	0
**OTU0286**	1	1	0	0	0	0	0	0	0	0	0	0	0	0	0	0	0	0
**OTU0287**	0	1	2	1	0	0	0	0	0	3	2	0	0	1	0	0	0	1
**OTU0288**	0	1	0	0	0	0	1	0	0	0	0	0	0	0	0	0	0	0
**OTU0289**	0	1	1	0	0	0	0	0	0	0	0	0	0	0	0	0	0	0
**OTU0290**	8	9	11	4	5	2	12	2	3	23	21	5	5	10	1	21	14	11
**OTU0291**	1	1	1	0	0	0	0	0	0	0	0	0	0	0	0	0	0	0
**OTU0292**	0	1	0	0	0	0	0	0	0	0	2	0	0	0	0	0	0	1
**OTU0293**	20	27	54	6	11	2	26	16	2	32	56	22	3	11	7	11	26	21
**OTU0294**	10	91	43	2	1	0	7	11	30	26	36	9	5	4	2	34	25	20
**OTU0295**	1	1	0	0	0	0	0	0	0	0	0	0	0	1	0	0	0	0
**OTU0296**	0	16	0	0	0	0	0	0	0	0	1	0	0	0	0	0	0	0
**OTU0297**	0	0	0	0	0	0	0	0	0	0	0	0	0	2	0	0	0	1
**OTU0298**	0	0	6	0	0	0	0	0	0	2	1	1	0	0	0	0	0	0
**OTU0299**	1	0	0	0	0	0	0	0	0	0	3	1	0	1	0	3	5	1
**OTU0300**	21	20	11	0	5	1	6	2	2	4	2	10	1	0	1	0	2	0
**OTU0301**	0	2	0	0	0	1	1	0	0	0	1	0	0	0	0	0	0	0
**OTU0302**	0	1	0	0	1	0	1	0	0	0	1	0	0	0	0	0	0	0
**OTU0303**	0	2	0	0	0	0	0	1	0	0	1	0	0	0	0	0	0	0
**OTU0304**	0	0	0	0	0	0	4	2	1	0	0	0	0	0	0	1	0	2
**OTU0305**	1	0	0	0	0	0	0	0	0	0	0	1	0	0	0	0	0	0
**OTU0306**	1	1	0	0	0	0	0	0	0	0	1	0	0	0	0	0	0	0
**OTU0307**	0	1	0	0	0	0	2	0	0	0	0	1	0	1	0	1	0	0

**OTU0308**	3	11	1	0	0	0	4	0	0	0	0	0	0	0	0	0	0	0
**OTU0309**	0	2	0	0	0	0	0	1	0	0	0	0	0	0	0	0	0	0
**OTU0310**	1	3	2	3	2	2	8	9	6	4	7	1	2	3	0	14	18	11
**OTU0311**	0	0	0	0	0	0	0	1	0	0	0	1	0	0	0	0	0	0
**OTU0312**	1	3	1	0	0	0	0	0	0	0	1	0	0	1	0	0	0	0
**OTU0313**	0	0	0	0	0	0	0	1	0	0	0	0	0	0	1	0	0	0
**OTU0314**	0	1	0	0	0	0	2	1	1	2	1	0	0	0	1	0	3	3
**OTU0315**	1	0	0	0	0	0	0	0	0	0	0	1	0	0	0	0	0	0
**OTU0316**	0	3	1	2	0	0	0	0	0	2	4	1	0	0	0	6	1	1
**OTU0317**	0	0	1	0	0	0	2	0	0	4	0	1	0	0	2	0	0	0
**OTU0318**	1	2	0	0	0	0	1	1	0	2	2	0	0	3	0	0	0	0
**OTU0319**	0	0	1	0	0	0	0	0	0	0	0	0	1	0	0	0	0	0
**OTU0320**	22	17	1717	3	7	7	7	14	7	562	1290	12	711	315	460	15	28	4
**OTU0321**	1	2	1	1	0	0	0	0	0	3	3	0	0	2	0	0	1	0
**OTU0322**	0	0	0	0	0	2	1	0	0	1	0	0	0	0	0	0	0	0
**OTU0323**	0	1	1	0	0	0	0	0	7	3	3	5	1	2	0	0	0	2
**OTU0324**	0	0	0	0	0	0	0	0	0	0	1	0	0	0	3	0	0	0
**OTU0325**	0	0	1	0	0	0	0	0	0	0	2	5	0	0	0	0	1	0
**OTU0326**	11	27	22	7	2	4	5	9	7	21	37	28	3	8	40	13	5	5
**OTU0327**	0	0	1	0	0	0	0	0	0	0	0	0	0	0	0	0	0	0
**OTU0328**	0	0	0	0	1	0	2	0	0	0	1	2	0	0	0	1	0	0
**OTU0329**	3	4	13	0	0	2	0	2	0	4	3	7	1	0	1	22	6	6
**OTU0330**	258	473	456	161	119	60	240	123	79	397	576	220	83	185	47	158	217	174
**OTU0331**	1	0	0	0	0	0	0	0	0	1	0	0	0	0	0	0	0	0
**OTU0332**	21	50	42	6	9	4	9	7	7	17	52	29	4	13	2	76	14	44
**OTU0333**	1	1	0	0	0	0	1	0	0	0	1	0	0	1	0	0	2	0
**OTU0334**	0	0	0	0	1	0	0	0	0	0	1	0	0	2	0	1	0	0
**OTU0335**	0	2	0	1	0	0	0	0	0	0	1	0	0	0	0	0	1	1
**OTU0336**	0	0	0	0	0	0	0	1	0	0	0	0	1	0	1	0	0	0
**OTU0337**	0	0	0	0	0	0	0	0	0	0	1	0	0	0	0	1	0	0
**OTU0338**	0	0	0	0	0	0	1	0	0	0	0	0	0	0	0	0	0	1
**OTU0339**	1	12	0	0	0	0	0	0	0	1	0	0	0	0	0	0	1	2
**OTU0340**	0	0	0	1	0	0	1	1	0	0	0	0	0	0	0	1	0	0
**OTU0341**	0	0	0	0	0	0	0	0	0	0	3	0	0	0	1	0	0	0
**OTU0342**	7	4	2	7	1	0	0	9	3	0	6	5	6	5	2	4	6	17
**OTU0343**	2	0	7	1	1	0	5	0	0	0	0	12	2	2	0	0	3	7
**OTU0344**	2	2	8	1	2	0	424	1	1	2	4	1	1	0	0	1	5	7
**OTU0345**	0	1	0	0	0	0	0	0	0	1	0	0	0	0	0	0	0	0
**OTU0346**	1	8	8	1	1	1	0	1	0	7	1	0	0	0	6	2	4	1

**OTU0347**	0	2	0	0	0	0	8	0	0	0	0	0	0	0	0	0	0	1
**OTU0348**	0	0	0	0	0	0	0	0	0	0	0	0	0	0	0	1	4	0
**OTU0349**	0	1	0	0	2	0	0	0	0	0	0	2	0	2	6	0	0	0
**OTU0350**	0	0	0	1	10	1	0	3	1	0	9	0	0	3	2	0	1	0
**OTU0351**	0	0	0	0	0	0	0	0	0	0	0	1	0	0	1	0	0	0
**OTU0352**	26	78	69	21	14	4	38	27	14	84	80	16	15	31	16	40	29	21
**OTU0353**	1	1	1	0	0	0	1	0	0	93	1	1	257	8	76	0	0	0
**OTU0354**	0	0	1	0	0	0	0	0	0	2	0	0	0	0	0	0	0	0
**OTU0355**	0	0	1	0	0	0	1	0	0	0	0	1	0	0	0	0	0	0
**OTU0356**	1	1	0	0	0	0	0	0	0	0	0	0	0	0	0	0	0	0
**OTU0357**	0	1	1	0	0	0	0	0	0	0	0	0	0	0	0	0	0	0
**OTU0358**	1	1	0	0	0	0	0	0	0	0	0	0	0	0	0	0	0	0
**OTU0359**	0	0	0	0	0	0	0	0	0	2	0	0	0	0	0	4	0	0
**OTU0360**	0	2	0	0	0	0	1	0	0	5	0	1	0	6	0	0	0	0
**OTU0361**	0	0	3	0	1	0	2	2	0	2	6	2	0	2	1	1	4	2
**OTU0362**	1	3	1	1	0	1	1	0	0	1	2	0	0	0	0	2	2	3
**OTU0363**	0	0	1	0	0	0	5	0	0	0	1	0	0	0	0	0	0	0
**OTU0364**	2	4	3	1	0	0	1	1	0	0	1	0	0	0	0	3	0	0
**OTU0365**	0	0	0	0	0	0	0	0	0	0	2	1	0	0	0	0	0	0
**OTU0366**	0	1	0	0	0	0	0	0	0	0	0	0	0	0	0	2	0	0
**OTU0367**	3	1	5	3	0	1	1	3	0	4	3	1	0	1	0	2	0	2
**OTU0368**	0	1	0	0	0	0	0	0	0	1	0	0	0	0	0	0	0	0
**OTU0369**	5	8	6	2	0	0	3	0	1	3	8	1	2	4	4	2	3	2
**OTU0370**	3	4	7	3	1	1	4	4	0	6	8	3	3	2	3	1	2	5
**OTU0371**	0	2	1	1	0	0	0	1	1	2	0	0	0	0	0	1	0	0
**OTU0372**	1	1	3	0	2	0	1	0	1	1	0	1	0	0	0	7	4	2
**OTU0373**	0	0	1	0	0	0	0	0	0	0	1	0	0	0	0	0	0	0
**OTU0374**	0	0	0	0	0	0	0	0	0	0	1	0	0	2	0	1	0	0
**OTU0375**	0	0	1	0	0	0	0	0	0	0	0	0	0	0	0	0	0	0
**OTU0376**	0	0	1	0	0	0	0	7	0	0	0	0	0	0	0	0	0	0
**OTU0377**	1	5	5	2	0	1	3	0	1	2	2	1	0	0	0	2	0	0
**OTU0378**	0	1	1	0	0	0	1	0	0	0	2	0	1	0	0	0	0	1
**OTU0379**	0	0	0	0	0	0	1	1	0	0	0	0	0	0	0	0	0	0
**OTU0380**	9	17	25	5	8	3	7	9	3	25	48	20	0	6	9	8	8	7
**OTU0381**	0	4	0	0	1	0	1	0	1	0	1	0	0	1	0	0	1	0
**OTU0382**	0	8	0	0	1	0	0	0	0	0	0	0	0	0	0	0	0	0
**OTU0383**	0	0	0	0	0	0	0	0	0	2	0	2	0	0	0	0	0	0
**OTU0384**	0	0	0	0	1	0	0	0	0	0	0	0	0	0	0	1	0	0
**OTU0385**	2	7	3	1	0	0	1	1	1	1	1	3	2	1	0	0	1	0

**OTU0386**	1	2	0	0	0	0	0	0	0	0	1	0	0	0	0	0	0	0
**OTU0387**	0	3	1	0	0	0	0	0	0	1	0	0	0	1	0	0	0	0
**OTU0388**	0	1	1	0	0	0	0	0	0	0	0	0	0	0	0	0	0	0
**OTU0389**	0	1	0	0	0	0	0	0	0	0	0	0	0	0	0	0	0	0
**OTU0390**	0	1	0	0	0	0	0	0	0	0	0	0	0	0	0	0	0	1
**OTU0391**	1	6	3	0	0	0	2	4	0	1	5	1	0	1	0	0	0	0
**OTU0392**	0	1	1	0	0	0	0	0	1	0	0	0	0	0	0	0	0	0
**OTU0393**	0	0	1	0	1	0	0	0	0	0	0	0	0	0	0	0	0	0
**OTU0394**	0	0	0	0	0	0	3	0	0	1	1	0	0	0	0	0	0	0
**OTU0395**	0	0	0	0	0	0	0	0	0	0	0	0	1	0	0	7	0	0
**OTU0396**	0	0	0	0	0	0	17	0	0	0	0	0	0	0	0	0	0	0
**OTU0397**	0	1	0	0	0	0	0	0	0	0	1	0	0	0	0	0	0	0
**OTU0398**	14	16	6	0	5	1	35	3	1	1	7	9	0	1	0	0	2	2
**OTU0399**	0	2	1	1	0	0	0	0	0	0	1	0	0	0	1	1	2	1
**OTU0400**	0	0	0	0	0	0	1	0	0	1	0	0	0	0	0	0	0	0
**OTU0401**	0	1	0	0	0	0	0	1	0	0	1	0	0	0	0	0	0	0
**OTU0402**	3	0	0	0	0	0	0	0	0	0	0	0	0	0	4	0	0	0
**OTU0403**	3	3	1	1	0	0	1	2	0	0	1	2	0	0	0	1	0	0
**OTU0404**	72	87	83	37	30	14	54	19	16	56	111	52	15	35	39	30	44	46
**OTU0405**	0	0	0	0	0	0	0	0	0	0	1	0	0	0	0	1	0	0
**OTU0406**	0	2	2	0	0	0	0	0	0	0	0	0	0	0	0	0	0	0
**OTU0407**	0	1	0	0	0	0	0	0	0	0	0	0	0	2	0	0	0	0
**OTU0408**	0	0	0	0	0	0	0	2	0	0	4	0	0	0	0	0	0	0
**OTU0409**	0	0	1	0	0	1	1	0	0	0	0	0	0	0	0	1	0	0
**OTU0410**	0	0	1	0	0	0	0	0	0	0	0	0	0	0	0	1	0	0
**OTU0411**	58	105	107	32	29	6	56	26	16	90	132	67	19	38	11	28	37	32
**OTU0412**	5	4	3	1	0	0	0	0	0	8	1	0	1	1	1	1	0	0
**OTU0413**	0	9	4	0	0	0	0	0	0	2	6	0	1	0	1	0	2	0
**OTU0414**	24	59	48	20	17	4	41	9	13	67	65	14	4	33	8	29	31	38
**OTU0415**	0	0	0	0	0	0	0	0	0	1	0	1	0	0	0	0	0	1
**OTU0416**	0	1	1	0	0	0	0	0	0	0	1	0	0	0	0	0	0	0
**OTU0417**	0	0	1	0	0	0	1	1	0	0	2	0	0	0	0	1	0	2
**OTU0418**	1	1	1	0	0	0	0	0	0	0	0	0	0	0	0	0	0	0
**OTU0419**	26	60	65	23	11	10	34	36	8	54	57	29	7	20	4	27	31	24
**OTU0420**	0	0	0	0	0	1	1	1	0	0	0	0	0	0	0	0	0	0
**OTU0421**	0	1	0	0	0	0	0	0	0	1	0	0	0	0	0	0	0	0
**OTU0422**	0	1	1	0	1	0	0	0	0	0	0	0	0	0	0	0	0	0
**OTU0423**	0	1	0	0	0	0	0	0	0	0	0	0	0	0	0	0	1	0
**OTU0424**	0	3	3	1	1	1	0	1	0	0	0	0	0	0	2	0	0	1

**OTU0425**	4	1	4	2	1	0	4	7	1	5	8	3	0	3	3	0	2	1
**OTU0426**	0	1	2	1	1	0	4	3	0	3	10	2	0	1	1	0	2	1
**OTU0427**	5	5	3	0	3	0	5	1	1	1	2	0	1	1	0	1	0	0
**OTU0428**	0	2	3	0	0	0	0	0	0	0	0	0	0	0	0	0	0	0
**OTU0429**	0	0	0	0	0	0	1	0	0	8	0	0	0	0	0	0	0	0
**OTU0430**	4	5	4	0	1	0	2	0	1	1	3	5	0	2	0	2	1	0
**OTU0431**	0	0	1	2	1	0	4	1	1	1	0	0	0	2	0	0	4	3
**OTU0432**	1	1	2	1	0	0	5	3	1	1	1	0	0	1	0	5	3	7
**OTU0433**	24	24	36	7	12	4	38	6	8	34	41	18	8	27	5	12	14	11
**OTU0434**	0	0	0	1	0	0	0	0	0	1	0	0	0	0	0	1	0	0
**OTU0435**	0	1	0	0	0	0	0	0	0	0	1	0	0	0	0	0	1	0
**OTU0436**	3	15	21	26	8	13	23	6	2	13	46	50	8	14	1	30	6	21
**OTU0437**	1	1	1	0	0	0	0	0	0	0	0	1	0	0	0	0	0	2
**OTU0438**	5	9	11	9	7	1	12	4	10	22	14	3	7	12	2	6	9	3
**OTU0439**	35	53	62	15	14	4	79	23	12	52	57	36	13	25	4	19	31	16
**OTU0440**	1	1	3	1	0	0	5	5	2	0	0	0	0	0	0	2	1	2
**OTU0441**	0	0	1	0	0	0	1	0	0	0	1	0	0	0	0	0	0	0
**OTU0442**	13	21	13	1	3	4	5	7	18	27	34	5	2	21	1	9	6	5
**OTU0443**	1	0	0	0	0	0	1	3	0	1	0	0	0	0	0	4	1	1
**OTU0444**	0	8	1	7	0	1	0	0	0	6	5	1	0	0	2	2	5	1
**OTU0445**	0	1	0	0	0	0	0	0	0	0	1	0	0	0	0	0	0	1
**OTU0446**	0	0	1	0	0	0	8	2	1	0	1	0	0	0	0	0	0	0
**OTU0447**	1	3	0	0	0	1	0	0	1	0	0	1	0	1	0	0	0	0
**OTU0448**	0	0	0	1	0	0	0	0	0	0	0	1	0	1	0	1	2	3
**OTU0449**	0	0	0	0	0	0	1	0	0	0	0	0	0	0	0	0	0	0
**OTU0450**	0	21	8	1	0	0	1	0	0	1	0	0	0	1	0	1	0	0
**OTU0451**	0	0	0	0	0	0	0	1	1	0	0	0	0	0	0	0	0	0
**OTU0452**	0	0	0	0	0	0	0	0	0	0	2	0	0	0	0	0	1	0
**OTU0453**	0	0	2	0	0	0	0	0	0	0	1	0	0	0	0	0	0	0
**OTU0454**	0	4	1	0	0	0	4	0	2	0	1	0	0	1	0	0	0	1
**OTU0455**	0	3	9	0	0	1	1	0	0	3	4	0	9	5	6	0	1	0
**OTU0456**	2	5	7	0	0	0	0	0	0	0	0	0	0	0	0	0	0	0
**OTU0457**	1	0	0	0	0	0	0	0	0	0	0	0	0	0	0	0	1	0
**OTU0458**	0	1	0	0	0	0	1	0	0	0	0	0	0	0	0	0	0	0
**OTU0459**	4	249	56	1	0	0	0	2	2	1	35	1	0	0	0	3	0	3
**OTU0460**	0	0	0	0	0	0	0	0	0	0	0	0	0	0	0	1	0	0
**OTU0461**	0	0	0	0	0	0	0	0	0	0	0	1	0	0	0	0	0	0
**OTU0462**	0	0	1	0	0	0	1	2	0	0	3	1	0	0	0	0	0	2
**OTU0463**	4	5	3	2	1	1	4	2	3	4	9	5	1	8	3	1	6	0

**OTU0464**	0	0	0	0	0	0	5	0	0	0	0	0	0	0	0	0	0	1
**OTU0465**	0	0	0	0	0	0	0	0	0	0	0	0	0	0	0	0	1	3
**OTU0466**	0	1	0	1	4	0	0	0	0	0	0	0	0	0	0	0	0	0
**OTU0467**	0	1	0	1	0	0	0	0	0	0	2	0	0	0	0	0	0	0
**OTU0468**	0	0	0	0	0	0	0	0	0	0	0	0	0	0	0	0	1	4
**OTU0469**	0	0	1	1	0	0	0	0	0	0	0	0	0	0	0	0	0	0
**OTU0470**	0	0	0	0	0	0	0	0	0	2	0	0	0	0	0	0	1	0
**OTU0471**	0	0	0	0	0	0	0	0	0	2	1	0	0	0	0	0	0	1
**OTU0472**	0	0	0	0	0	0	0	0	0	0	1	0	0	0	0	0	1	0
**OTU0473**	1	2	0	0	0	0	0	0	0	3	1	0	0	2	0	2	0	0
**OTU0474**	0	0	1	0	0	0	0	0	0	0	0	2	0	0	0	0	0	0
**OTU0475**	1	1	1	0	0	0	0	0	0	0	0	0	0	0	0	0	0	0
**OTU0476**	0	1	0	0	0	0	0	1	0	0	0	0	0	0	0	0	0	0
**OTU0477**	0	0	1	0	0	0	0	0	0	0	1	0	0	0	1	0	0	1
**OTU0478**	0	0	1	0	0	0	0	0	0	0	1	0	0	0	0	0	0	0
**OTU0479**	3	36	21	4	2	1	2	3	13	9	10	3	0	3	3	4	13	3
**OTU0480**	2	6	0	0	3	0	5	0	3	0	3	0	0	1	1	0	7	9
**OTU0481**	0	0	0	0	1	0	0	0	0	0	0	0	0	0	0	0	0	1
**OTU0482**	14	23	12	3	2	4	9	3	3	6	14	17	1	0	4	1	6	1
**OTU0483**	0	0	1	0	0	0	0	0	0	1	0	0	0	0	0	0	0	0
**OTU0484**	2	4	1	0	0	0	0	0	1	1	3	0	0	0	1	1	0	0
**OTU0485**	2	0	8	0	0	0	0	0	0	1	1	1	8	4	7	0	0	0
**OTU0486**	1	0	1	0	0	0	0	0	0	0	0	1	0	0	0	0	0	0
**OTU0487**	0	0	2	0	0	0	0	0	0	0	1	0	1	2	1	0	0	0
**OTU0488**	5	4	13	9	1	3	358	173	19	4	17	4	0	3	0	8	5	0
**OTU0489**	11	4	29	1	0	1	4	2	2	14	9	10	1	2	2	3	2	3
**OTU0490**	0	0	0	0	2	0	0	2	0	0	1	0	0	0	0	0	0	0
**OTU0491**	1	0	2	0	0	0	2	0	0	1	2	1	1	1	0	2	0	0
**OTU0492**	20	25	27	4	9	3	21	4	1	12	30	9	4	4	2	7	6	14
**OTU0493**	0	0	0	0	0	0	0	0	0	0	0	0	0	0	0	1	0	2
**OTU0494**	0	0	0	0	0	0	0	0	0	0	0	0	0	0	0	0	1	0
**OTU0495**	1	1	3	0	0	0	0	1	0	0	2	1	0	0	0	1	0	0
**OTU0496**	0	0	0	0	0	0	3	2	0	0	1	0	0	0	0	0	0	0
**OTU0497**	7	9	7	3	2	1	7	3	2	8	9	9	2	4	2	3	1	1
**OTU0498**	0	0	0	0	0	0	0	0	0	0	0	1	0	0	0	0	0	0
**OTU0499**	0	0	0	0	0	0	1	2	0	0	2	1	0	0	2	2	0	1
**OTU0500**	0	1	0	0	0	0	0	0	0	0	0	0	0	0	0	0	13	0
**OTU0501**	0	0	0	1	0	0	0	0	0	0	0	0	0	0	0	0	0	0
**OTU0502**	0	0	0	0	0	1	0	0	0	1	0	1	0	0	0	0	0	0

**OTU0503**	1	9	2	0	0	1	6	2	3	3	2	1	1	2	1	0	1	2
**OTU0504**	3	2	1	0	0	1	1	0	0	3	1	1	0	1	0	1	0	0
**OTU0505**	0	1	1	0	0	0	0	0	0	0	0	1	0	0	0	0	0	0
**OTU0506**	0	11	14	0	2	0	5	9	0	0	3	5	0	0	2	8	3	0
**OTU0507**	1	0	12	0	0	0	0	0	0	0	0	0	2	1	1	0	0	0
**OTU0508**	24	26	34	9	10	5	26	7	9	34	43	24	5	6	1	16	13	16
**OTU0509**	1	0	1	0	0	0	0	0	0	0	3	0	0	0	0	0	0	0
**OTU0510**	1	0	1	0	0	0	0	0	0	1	0	0	0	0	0	0	0	1
**OTU0511**	2	3	3	1	0	3	3	0	0	4	3	3	1	1	0	5	1	1
**OTU0512**	1	0	2	0	0	0	1	0	0	0	1	0	0	0	0	0	2	0
**OTU0513**	0	0	0	0	0	0	1	0	1	0	0	0	0	0	0	2	0	1
**OTU0514**	0	2	0	0	0	0	0	1	0	0	0	0	0	0	0	2	0	0
**OTU0515**	1	0	0	2	0	0	1	7	0	0	3	0	0	0	0	0	0	0
**OTU0516**	5	0	9	0	5	1	0	0	0	4	1	2	2	1	0	1	1	0
**OTU0517**	0	0	1	0	0	0	0	1	0	0	0	1	0	0	0	0	0	1
**OTU0518**	0	0	0	1	0	0	1	1	0	0	2	0	0	0	0	0	0	3
**OTU0519**	1	0	1	0	0	0	0	0	0	0	0	0	0	0	0	0	0	0
**OTU0520**	0	0	1	0	0	0	0	1	0	5	0	0	0	0	0	0	0	0
**OTU0521**	0	3	0	0	0	0	0	0	0	0	0	0	0	0	0	0	0	0
**OTU0522**	0	0	1	0	0	0	0	0	0	0	0	0	0	0	0	0	0	0
**OTU0523**	0	0	0	0	0	0	0	0	1	0	0	0	0	0	1	0	0	0
**OTU0524**	2	4	3	1	0	0	1	2	0	2	7	1	0	0	1	0	1	1
**OTU0525**	0	2	4	0	0	0	0	2	1	2	0	0	0	0	0	0	1	1
**OTU0526**	1	1	2	0	1	1	0	0	0	1	0	0	0	1	10	2	1	0
**OTU0527**	0	0	0	0	0	0	0	0	0	0	0	0	0	0	1	0	1	0
**OTU0528**	0	5	0	0	0	0	1	0	0	0	0	0	0	0	1	0	0	1
**OTU0529**	9	9	24	10	3	0	11	1	3	11	24	6	3	6	4	15	4	4
**OTU0530**	1	0	3	0	0	0	1	0	0	2	1	0	1	0	0	1	0	2
**OTU0531**	43	54	54	15	18	9	34	20	16	39	86	30	12	20	7	21	21	24
**OTU0532**	6	13	10	1	4	0	8	2	0	14	11	1	2	3	0	6	1	2
**OTU0533**	0	0	1	0	0	1	0	0	6	0	2	3	0	0	0	0	0	0
**OTU0534**	0	0	0	0	0	0	0	0	0	0	0	0	0	0	0	0	1	0
**OTU0535**	5	4	4	1	0	4	8	3	1	4	5	10	1	2	0	0	1	0
**OTU0536**	0	0	0	0	0	0	1	0	0	1	0	0	0	0	0	0	0	0
**OTU0537**	0	0	1	0	0	0	0	0	0	0	0	0	0	1	0	0	0	0
**OTU0538**	0	0	0	0	0	0	0	0	0	0	0	0	0	1	5	0	0	0
**OTU0539**	1	1	0	0	0	0	0	0	0	1	0	0	0	0	0	1	0	1
**OTU0540**	1	2	4	0	1	0	2	0	0	3	2	0	0	0	1	2	4	0
**OTU0541**	3	0	0	0	0	0	3	0	0	2	0	0	0	0	0	0	0	0

**OTU0542**	1	6	2	1	1	0	3	0	1	4	7	0	0	2	0	3	5	0
**OTU0543**	0	0	0	0	0	0	0	0	0	0	1	0	0	0	0	0	2	0
**OTU0544**	3	10	14	0	2	0	6	3	0	7	7	3	1	3	1	1	5	3
**OTU0545**	0	0	0	0	0	1	0	1	0	2	1	0	1	0	7	0	0	0
**OTU0546**	0	0	7	0	0	0	0	0	0	0	0	0	0	0	0	0	0	0
**OTU0547**	2	0	0	0	0	0	0	0	0	0	1	0	0	1	0	2	0	0
**OTU0548**	2	2	5	0	0	1	2	0	1	3	4	0	0	2	1	1	4	2
**OTU0549**	0	0	0	0	0	1	0	2	0	0	0	0	0	0	0	0	0	0
**OTU0550**	0	0	0	0	0	0	1	0	0	0	0	0	0	0	0	1	0	0
**OTU0551**	0	1	0	0	0	0	0	0	0	0	0	0	0	0	0	0	0	1
**OTU0552**	0	5	7	1	1	0	1	1	0	10	5	0	1	2	2	4	4	2
**OTU0553**	0	1	1	0	0	0	1	0	0	0	0	0	0	0	0	0	0	0
**OTU0554**	0	1	0	0	0	0	0	0	0	0	2	0	0	0	0	0	0	2
**OTU0555**	1	0	0	0	0	0	0	0	0	0	0	0	0	11	0	2	1	0
**OTU0556**	0	2	4	0	0	0	8	7	1	4	1	1	0	0	0	12	4	5
**OTU0557**	23	57	37	10	11	2	16	4	9	20	36	15	5	23	1	13	19	13
**OTU0558**	180	233	389	124	80	57	276	105	82	259	381	187	37	147	36	122	160	102
**OTU0559**	1	1	0	0	0	0	0	0	0	0	3	0	0	0	0	0	0	0
**OTU0560**	0	0	1	1	0	0	0	0	0	2	0	0	0	0	0	0	2	1
**OTU0561**	2	0	0	0	0	0	0	0	0	6	0	2	0	0	1	2	0	4
**OTU0562**	0	2	0	0	0	0	1	0	0	1	0	0	0	0	0	0	0	0
**OTU0563**	0	0	0	0	0	0	0	0	0	1	0	1	0	0	0	0	0	0
**OTU0564**	0	0	0	0	0	0	1	0	0	0	2	0	0	0	1	0	0	0
**OTU0565**	1	1	0	0	0	0	0	1	0	0	0	0	0	0	0	0	0	0
**OTU0566**	0	2	0	0	0	0	0	1	0	0	0	0	0	0	0	0	0	0
**OTU0567**	5	2	2	1	0	0	0	0	1	0	4	0	1	1	0	0	0	0
**OTU0568**	0	2	1	0	0	0	0	0	0	1	0	0	0	0	0	0	1	0
**OTU0569**	36	68	33	7	5	6	17	16	4	23	43	27	6	2	1	2	5	11
**OTU0570**	0	1	1	0	0	0	0	0	0	0	2	0	0	0	0	0	0	0
**OTU0571**	0	2	0	1	0	0	0	0	0	0	2	1	0	0	0	0	0	0
**OTU0572**	4	1	0	1	1	3	1	0	0	0	3	1	1	0	29	0	0	0
**OTU0573**	4	21	19	9	7	2	18	4	4	21	25	5	1	15	7	29	18	14
**OTU0574**	0	0	0	0	0	0	0	1	1	0	1	0	1	0	0	0	1	1
**OTU0575**	0	2	3	0	0	0	1	0	0	0	2	1	0	0	0	1	1	1
**OTU0576**	0	1	0	0	0	0	0	0	0	0	0	0	0	0	0	1	0	1
**OTU0577**	0	23	5	0	0	0	1	0	1	2	5	2	0	0	0	0	1	1
**OTU0578**	0	1	0	0	0	0	0	0	0	0	0	2	1	0	0	0	0	0
**OTU0579**	0	5	6	0	0	0	0	0	5	5	1	2	1	0	0	1	0	0
**OTU0580**	13	10	26	5	3	4	13	2	15	39	16	3	0	3	152	11	1	5

**OTU0581**	3	17	11	11	4	2	19	7	5	19	17	4	0	8	2	20	17	11
**OTU0582**	2	1	0	0	0	0	0	0	0	0	0	0	0	0	0	1	0	1
**OTU0583**	0	0	0	0	0	0	0	0	0	5	2	0	0	0	2	0	0	0
**OTU0584**	5	19	9	17	3	2	8	6	4	20	19	0	1	13	1	12	19	20
**OTU0585**	0	0	0	0	0	0	6	0	0	0	0	0	0	1	0	0	0	0
**OTU0586**	1	0	0	0	0	0	0	1	0	2	1	0	0	2	2	0	0	2
**OTU0587**	0	0	0	0	0	0	0	0	0	0	0	0	0	0	0	0	0	1
**OTU0588**	5	20	12	0	0	0	0	0	0	0	0	1	0	0	0	0	0	0
**OTU0589**	0	0	2	0	0	0	0	0	1	1	1	1	0	0	0	0	0	0
**OTU0590**	0	1	0	0	0	0	0	0	0	0	0	0	0	0	0	0	3	0
**OTU0591**	31	25	67	0	15	7	86	44	60	35	73	60	39	14	48	9	15	7
**OTU0592**	11	20	24	2	1	7	8	8	4	12	28	14	3	5	3	0	1	3
**OTU0593**	1	0	0	0	0	0	0	0	0	0	0	0	0	0	0	0	0	4
**OTU0594**	0	0	0	0	0	0	0	0	0	0	1	0	0	0	1	0	0	0
**OTU0595**	0	1	0	0	0	0	0	0	0	0	0	0	0	2	0	0	0	0
**OTU0596**	0	1	1	0	0	0	0	1	1	1	0	1	0	0	0	0	0	1
**OTU0597**	0	0	0	0	0	0	0	1	0	0	0	0	0	0	0	0	0	0
**OTU0598**	7	2	9	2	2	0	1	2	2	10	12	2	0	6	0	5	5	5
**OTU0599**	54	60	2040	13	64	23	30	25	13	1701	1433	78	115	175	109	9	18	8
**OTU0600**	2	2	100	0	1	0	2	0	0	22	74	2	52	10	44	0	2	0
**OTU0601**	0	1	3	0	0	0	0	0	0	1	1	0	0	0	0	0	0	0
**OTU0602**	0	0	0	0	0	0	0	0	0	0	0	0	0	0	0	0	3	0
**OTU0603**	0	0	7	0	0	1	0	0	0	2	1	0	3	0	0	0	0	0
**OTU0604**	0	0	0	0	0	0	0	0	0	0	1	0	0	0	0	0	0	0
**OTU0605**	0	1	3	0	1	0	0	1	0	0	2	0	0	1	2	0	1	0
**OTU0606**	1	0	0	1	0	0	0	0	0	0	0	0	0	0	0	0	0	0
**OTU0607**	0	1	1	0	0	0	0	0	0	1	2	1	2	1	1	0	0	0
**OTU0608**	0	0	1	0	0	0	1	0	0	1	0	0	0	0	0	0	0	0
**OTU0609**	0	0	1	0	0	0	42	0	0	0	1	0	1	0	1	0	0	0
**OTU0610**	1	1	0	0	0	0	0	0	0	0	0	0	0	0	0	0	0	0
**OTU0611**	1	1	1	0	0	0	0	0	0	1	0	0	0	0	0	0	0	0
**OTU0612**	10	12	98	1	5	8	15	9	8	260	76	5	30	16	11	6	4	3
**OTU0613**	0	0	1	0	0	0	0	0	0	0	0	0	0	0	0	0	0	0
**OTU0614**	3	0	0	0	0	0	0	0	0	0	0	1	0	0	0	0	0	0
**OTU0615**	2	2	5	0	1	1	1	0	0	1	2	0	3	1	2	0	1	0
**OTU0616**	1	0	2	0	0	0	0	0	1	0	0	0	0	0	0	0	1	0
**OTU0617**	0	0	0	0	0	0	0	0	0	0	11	0	0	0	0	0	1	0
**OTU0618**	1	2	0	0	0	0	0	3	1	2	2	1	0	0	0	0	0	0
**OTU0619**	0	0	0	0	0	0	0	1	1	0	2	0	0	0	0	0	0	0

**OTU0620**	2	6	5	0	3	0	5	2	0	2	4	3	2	2	1	0	3	0
**OTU0621**	0	2	1	0	0	0	1	0	0	0	1	0	0	0	0	0	0	0
**OTU0622**	0	0	0	0	0	0	0	0	0	1	1	0	0	0	0	0	0	0
**OTU0623**	1	1	2	0	1	0	0	0	1	0	1	0	0	0	0	1	0	0
**OTU0624**	8	19	23	6	2	1	20	2	7	17	28	15	4	11	0	10	10	9
**OTU0625**	5	8	11	1	0	1	1	2	2	9	9	12	0	3	2	12	5	22
**OTU0626**	2	3	0	0	0	0	1	1	2	1	3	1	0	0	2	2	2	0
**OTU0627**	0	0	0	0	0	0	1	2	0	0	0	1	0	0	0	0	0	0
**OTU0628**	0	0	0	0	0	0	0	0	0	1	0	0	0	0	1	0	0	0
**OTU0629**	1	1	1	1	0	0	0	1	0	1	4	0	0	0	0	0	0	1
**OTU0630**	0	0	0	0	0	0	2	2	0	0	0	0	0	0	0	0	0	0
**OTU0631**	7	10	16	2	7	9	9	6	8	34	37	9	1	24	2	10	11	13
**OTU0632**	8	6	8	5	0	2	2	2	0	12	8	2	2	2	0	5	5	9
**OTU0633**	0	0	0	0	0	0	0	0	0	0	4	0	0	0	0	0	0	0
**OTU0634**	0	1	0	1	0	0	0	0	0	0	0	0	0	0	0	0	0	0
**OTU0635**	5	47	13	0	0	1	5	1	9	3	10	1	0	0	0	1	6	2
**OTU0636**	35	66	101	44	21	8	34	30	6	70	108	32	8	53	18	59	65	51
**OTU0637**	30	72	62	47	21	10	35	10	5	89	96	23	11	31	9	40	52	54
**OTU0638**	6	4	3	0	1	1	1	1	2	2	2	5	0	1	0	0	0	0
**OTU0639**	1	0	0	0	0	0	0	0	0	0	0	0	0	0	0	1	0	0
**OTU0640**	8	7	11	1	1	1	2	3	0	9	9	11	1	1	1	0	0	0
**OTU0641**	5	7	8	0	1	1	2	2	0	6	7	3	0	3	3	4	5	3
**OTU0642**	1	0	0	0	0	0	1	0	0	0	0	0	0	0	0	0	0	0
**OTU0643**	2	1	0	0	0	0	1	0	0	0	1	0	0	0	0	0	0	0
**OTU0644**	0	0	0	0	0	0	0	0	0	0	0	1	0	0	0	0	0	0
**OTU0645**	1	1	0	0	1	0	0	0	0	0	2	1	0	0	0	0	0	0
**OTU0646**	0	1	3	1	0	0	0	1	1	1	2	2	0	0	0	0	0	1
**OTU0647**	2	1	5	0	1	0	3	2	1	1	1	3	0	1	2	0	1	0
**OTU0648**	0	0	0	0	0	0	0	0	0	1	0	0	0	0	0	0	0	0
**OTU0649**	3	0	1	1	2	0	1	1	1	5	4	5	0	2	0	1	4	4
**OTU0650**	0	0	1	0	0	0	0	0	0	0	0	0	0	1	0	0	0	0
**OTU0651**	0	0	1	0	0	0	0	0	0	0	0	1	0	0	0	0	0	1
**OTU0652**	0	3	10	0	0	0	0	0	0	0	3	3	0	0	0	13	0	3
**OTU0653**	0	0	1	0	4	0	0	0	0	0	0	1	0	2	4	0	0	0
**OTU0654**	2	2	0	0	2	0	0	0	0	0	3	0	0	0	3	0	0	0
**OTU0655**	1	2	4	1	0	1	2	1	1	1	1	0	0	6	0	3	1	0
**OTU0656**	0	0	1	0	0	0	0	1	0	0	0	1	0	1	0	0	0	0
**OTU0657**	0	1	0	0	0	1	0	0	0	0	0	0	0	0	0	0	0	0
**OTU0658**	0	0	3	0	0	0	0	0	0	0	3	0	3	1	5	0	0	0

**OTU0659**	17	32	33	1	10	0	7	4	5	26	44	8	5	6	2	7	5	2
**OTU0660**	0	0	0	0	0	0	0	0	0	0	1	0	0	0	0	0	1	0
**OTU0661**	0	2	0	0	0	0	5	0	0	4	1	1	1	1	1	9	5	6
**OTU0662**	0	2	0	0	0	0	0	0	0	0	0	0	0	0	0	0	0	2
**OTU0663**	177	315	359	49	102	41	157	74	58	320	371	154	49	101	2998	137	142	131
**OTU0664**	0	0	4	0	0	0	0	0	0	1	0	2	0	1	1	2	0	1
**OTU0665**	0	0	0	0	0	0	0	0	0	2	0	0	0	1	0	2	0	0
**OTU0666**	0	0	0	0	0	0	0	0	0	0	6	0	0	0	0	0	0	0
**OTU0667**	0	0	7	0	0	0	0	0	0	0	0	0	0	0	0	0	0	1
**OTU0668**	0	4	2	1	0	1	0	0	2	2	9	0	0	0	0	0	1	0
**OTU0669**	0	1	1	0	5	0	1	0	5	1	0	2	0	0	155	2	0	1
**OTU0670**	0	0	1	0	0	0	1	1	0	1	1	0	0	0	1	1	0	1
**OTU0671**	59	110	122	74	22	16	48	54	29	167	224	62	20	82	24	74	70	74
**OTU0672**	0	2	0	0	0	0	0	2	0	0	0	0	0	0	0	0	0	0
**OTU0673**	0	0	1	0	0	0	0	0	0	0	2	0	0	0	0	1	0	0
**OTU0674**	22	24	1675	1	5	5	9	8	6	587	1193	11	675	312	352	19	33	3
**OTU0675**	0	5	4	0	1	0	2	2	0	8	5	1	0	0	2	4	0	2
**OTU0676**	5	13	13	4	2	3	6	3	2	8	12	8	5	5	1	2	2	1
**OTU0677**	0	0	2	1	0	0	2	1	0	1	1	0	0	0	0	6	0	0
**OTU0678**	4	2	2	3	1	0	5	1	1	4	7	3	1	2	2	1	2	1
**OTU0679**	0	0	0	0	2	0	0	0	0	0	2	0	0	0	0	0	0	0
**OTU0680**	0	0	0	0	0	2	1	0	0	1	0	0	1	0	0	0	0	0
**OTU0681**	0	10	2	0	0	0	0	0	0	0	1	0	0	0	0	0	0	1
**OTU0682**	0	1	1	0	1	0	0	0	1	0	0	0	0	0	0	0	0	0
**OTU0683**	508	783	787	215	207	132	441	270	146	676	1053	437	107	337	77	341	413	337
**OTU0684**	0	0	0	0	0	0	0	1	0	0	0	0	0	0	1	0	0	0
**OTU0685**	0	2	2	0	2	1	2	1	2	0	2	0	0	1	0	3	0	0
**OTU0686**	0	0	0	0	0	0	0	0	0	0	1	0	0	1	2	0	0	0
**OTU0687**	0	0	0	0	0	0	0	0	0	1	0	0	0	0	0	0	0	0
**OTU0688**	0	1	0	0	0	0	4	8	2	0	0	0	0	0	0	0	0	0
**OTU0689**	0	4	0	0	0	0	0	0	0	0	0	0	0	0	0	0	1	0
**OTU0690**	3	3	2	0	0	0	1	0	0	0	5	2	0	0	0	1	0	0
**OTU0691**	0	0	0	0	0	0	1	0	0	0	1	0	0	0	0	0	0	0
**OTU0692**	0	0	0	0	0	0	1	0	0	1	0	1	0	0	0	0	0	0
**OTU0693**	2	2	4	0	1	0	12	25	2	5	9	3	1	3	1	3	2	6
**OTU0694**	0	0	2	0	0	0	0	1	0	1	0	0	0	0	0	0	1	0
**OTU0695**	0	0	0	0	0	0	0	0	0	0	0	0	0	1	0	0	0	0
**OTU0696**	12	28	28	10	18	5	14	13	13	19	42	16	6	5	6	4	10	12
**OTU0697**	128	169	284	86	56	19	138	68	72	225	324	96	33	117	22	186	124	64

**OTU0698**	1	3	9	1	1	0	4	2	0	5	2	2	1	2	1	1	5	1
**OTU0699**	2	5	6	5	3	1	3	0	0	4	11	3	2	3	1	4	2	1
**OTU0700**	1	1	1	0	2	0	1	0	3	1	4	0	0	1	0	1	6	3
**OTU0701**	0	1	0	0	0	0	0	0	0	1	2	0	0	2	1	0	0	0
**OTU0702**	5	8	5	1	0	2	3	3	2	0	7	1	0	1	0	0	3	1
**OTU0703**	1	1	0	0	0	0	0	0	0	1	0	0	0	0	0	0	0	0
**OTU0704**	0	0	1	0	0	0	0	0	0	0	1	0	0	0	0	0	0	0
**OTU0705**	18	25	21	15	9	4	115	71	55	19	41	14	2	10	4	156	123	85
**OTU0706**	0	3	1	0	0	0	0	0	0	0	1	1	0	0	0	0	0	0
**OTU0707**	2	0	1	0	0	0	0	1	1	0	0	0	0	1	21	0	0	0
**OTU0708**	0	10	0	0	0	0	0	0	0	0	8	0	0	0	0	0	0	0
**OTU0709**	0	0	1	0	0	0	0	0	0	1	0	0	0	0	0	0	0	0
**OTU0710**	2	4	69	0	0	2	1	2	1	12	33	2	99	58	65	2	4	1
**OTU0711**	8	43	8	0	1	0	3	8	4	5	21	1	0	3	0	3	2	0
**OTU0712**	6	6	7	4	6	0	1	2	4	4	12	4	2	4	1	2	9	4
**OTU0713**	38	30	19	0	0	0	0	1	0	12	20	18	0	0	1	1	1	0
**OTU0714**	102	135	143	38	44	16	568	358	237	128	200	76	29	73	20	762	816	585
**OTU0715**	1	1	0	0	4	0	0	0	0	0	0	0	0	0	0	0	0	0
**OTU0716**	132	413	269	49	45	17	70	32	38	185	302	106	27	66	20	76	77	32
**OTU0717**	0	0	1	0	0	0	2	0	0	2	1	0	0	0	0	1	0	1
**OTU0718**	98	63	33	3	2	2	383	5	2	19	48	38	1	5	5	4	4	14
**OTU0719**	11	18	17	14	9	1	70	58	36	21	17	16	3	4	0	60	89	44
**OTU0720**	0	1	0	0	0	0	0	0	0	0	1	0	0	0	0	0	0	0
**OTU0721**	1	1	1	0	0	0	0	0	0	0	1	1	0	0	0	0	0	0
**OTU0722**	1	3	2	1	0	0	1	2	1	2	2	0	0	1	1	0	1	2
**OTU0723**	1	1	0	0	1	1	0	0	0	1	1	0	0	1	13	0	0	0
**OTU0724**	0	0	0	0	0	0	0	0	0	1	2	0	0	0	0	0	0	0
**OTU0725**	6	5	5	0	0	0	1	0	2	9	7	4	1	4	0	1	4	4
**OTU0726**	0	0	0	0	0	0	2	2	1	0	6	2	0	2	0	2	0	2
**OTU0727**	0	0	3	0	0	0	0	0	0	0	0	0	0	0	0	0	3	0
**OTU0728**	1	0	0	0	0	0	0	0	0	0	0	0	0	0	0	3	0	0
**OTU0729**	0	0	1	0	0	0	1	0	1	0	0	0	0	0	0	0	0	0
**OTU0730**	1	6	8	3	2	0	0	0	0	5	18	1	1	0	3	4	0	2
**OTU0731**	1	6	2	0	0	1	1	0	0	1	1	0	0	0	1	0	2	0
**OTU0732**	4	1	3	0	2	0	1	4	0	1	2	3	0	0	3	2	1	0
**OTU0733**	1	1	0	0	0	1	1	0	0	2	0	0	0	0	0	0	0	0
**OTU0734**	1	0	0	0	1	0	1	0	0	1	1	0	0	0	0	0	0	1
**OTU0735**	8	21	4	3	0	0	10	5	7	5	12	9	0	2	3	1	1	0
**OTU0736**	0	1	0	0	0	0	0	0	0	0	0	1	0	1	0	0	0	0

**OTU0737**	164	857	391	70	51	24	78	61	34	182	457	95	36	76	24	80	83	89
**OTU0738**	0	2	4	4	1	1	2	0	0	4	2	0	0	0	0	1	1	4
**OTU0739**	0	3	3	0	0	0	0	0	0	4	5	0	0	1	0	0	2	1
**OTU0740**	0	1	0	0	1	0	0	0	0	0	0	1	0	0	0	0	0	0
**OTU0741**	16	18	19	4	7	4	6	8	4	6	21	12	2	2	3	4	6	6
**OTU0742**	0	0	0	0	0	0	0	0	0	0	0	0	0	0	0	0	7	1
**OTU0743**	5	5	4	2	3	0	3	1	1	9	6	3	0	1	0	4	5	1
**OTU0744**	0	0	0	0	0	0	0	0	1	0	0	1	0	0	0	0	0	0
**OTU0745**	0	0	0	0	0	0	2	0	0	0	1	0	0	0	0	0	0	0
**OTU0746**	20	24	34	11	9	2	23	12	5	23	33	9	6	13	6	17	24	24
**OTU0747**	0	2	0	0	0	0	0	0	0	0	1	0	0	0	0	0	0	0
**OTU0748**	0	5	0	0	0	0	0	0	0	0	11	0	0	0	0	0	0	0
**OTU0749**	0	1	1	1	0	3	4	8	3	1	3	2	0	0	16	2	2	4
**OTU0750**	9	33	3	3	2	2	4	3	1	3	11	3	0	0	0	0	4	1
**OTU0751**	0	0	0	0	0	0	0	0	0	0	1	0	0	1	0	0	0	0
**OTU0752**	1	1	0	0	0	0	0	0	0	0	1	0	0	1	0	1	0	1
**OTU0753**	0	1	0	0	0	0	0	1	0	0	0	0	0	0	0	0	0	0
**OTU0754**	1	1	1	0	0	0	0	0	0	1	2	1	1	0	1	1	0	0
**OTU0755**	0	0	2	1	0	0	3	0	0	1	3	0	0	0	0	0	0	0
**OTU0756**	0	0	0	0	0	0	0	0	0	1	0	0	0	0	0	0	0	0
**OTU0757**	0	1	1	0	0	0	0	0	0	0	3	0	0	0	0	1	2	1
**OTU0758**	0	0	0	0	0	0	1	1	0	0	1	0	0	0	0	0	0	0
**OTU0759**	0	6	0	1	0	0	0	0	0	0	0	0	0	0	0	0	1	0
**OTU0760**	0	1	0	0	0	0	0	0	0	1	0	0	1	0	0	0	0	0
**OTU0761**	0	0	0	0	0	0	1	0	0	0	0	0	0	0	0	0	0	0
**OTU0762**	0	0	1	0	0	0	0	0	0	0	0	0	0	0	0	0	3	4
**OTU0763**	0	0	5	0	1	0	0	0	0	0	0	0	0	0	0	0	0	0
**OTU0764**	11	12	10	3	2	0	8	1	4	13	17	3	1	6	5	3	5	8
**OTU0765**	0	0	7	0	0	0	0	0	0	2	2	0	2	0	1	0	0	0
**OTU0766**	4	8	4	1	0	1	4	1	0	6	7	4	1	1	0	0	0	1
**OTU0767**	0	1	1	0	0	0	0	0	0	0	0	0	0	1	0	1	0	0
**OTU0768**	1	0	0	0	1	0	0	0	0	0	0	0	0	0	0	0	0	0
**OTU0769**	0	2	0	0	0	0	0	0	0	0	2	0	0	0	0	0	0	0
**OTU0770**	2	8	4	1	0	1	5	1	3	4	9	4	0	1	1	2	6	2
**OTU0771**	2	0	2	1	1	1	2	0	1	3	6	1	0	1	0	1	1	2
**OTU0772**	1	0	0	1	0	0	0	1	0	0	1	0	0	1	0	0	0	0
**OTU0773**	1	0	2	0	0	0	0	0	1	1	4	0	0	0	0	0	1	0
**OTU0774**	778	1100	276	9	324	32	375	476	270	139	288	322	95	85	66	15	20	11
**OTU0775**	155	270	336	25	52	17	88	77	37	262	427	155	22	45	15	90	193	149

**OTU0776**	0	0	0	0	0	0	0	0	0	1	1	0	0	0	0	0	0	0
**OTU0777**	1	4	1	0	0	0	0	0	0	0	1	0	0	0	0	0	0	0
**OTU0778**	0	0	0	0	1	0	0	0	0	2	0	3	0	2	0	0	0	0
**OTU0779**	0	5	0	0	0	0	0	0	0	0	2	0	0	0	0	0	0	0
**OTU0780**	1	3	0	0	1	0	1	1	1	3	2	0	0	0	1	1	0	0
**OTU0781**	0	0	3	1	1	0	3	0	0	4	3	0	0	1	0	1	5	2
**OTU0782**	0	0	1	0	0	0	0	0	0	0	1	0	0	1	0	0	0	1
**OTU0783**	0	0	0	1	0	0	1	0	0	0	0	0	0	0	0	0	0	0
**OTU0784**	0	5	0	0	1	0	0	1	0	0	0	0	0	0	0	1	0	1
**OTU0785**	1	1	1	0	2	0	1	0	0	1	0	0	0	0	0	1	0	0
**OTU0786**	2	1	2	0	0	1	1	2	0	2	1	2	0	0	0	6	1	0
**OTU0787**	3	10	18	0	6	1	1	1	0	17	19	11	2	4	2	0	1	0
**OTU0788**	0	7	1	0	0	0	1	1	1	0	2	0	1	0	0	1	0	0
**OTU0789**	0	1	2	0	0	0	2	0	0	0	1	0	1	0	0	0	0	1
**OTU0790**	0	0	0	0	0	0	0	0	0	0	0	0	0	0	0	0	1	2
**OTU0791**	0	1	1	0	0	0	0	0	0	0	0	0	0	1	0	0	1	0
**OTU0792**	5	1	2	0	1	1	3	2	0	0	0	5	0	1	0	1	0	1
**OTU0793**	0	0	0	0	0	0	0	1	0	0	0	0	0	0	0	1	0	0
**OTU0794**	0	0	1	0	0	0	6	0	0	0	0	0	0	0	0	0	0	0
**OTU0795**	1	0	0	0	0	0	0	0	0	0	0	0	0	3	0	0	0	0
**OTU0796**	0	1	0	0	0	0	1	0	0	0	0	0	0	0	0	0	0	0
**OTU0797**	1	2	1	0	0	0	2	0	0	6	6	1	1	2	0	2	1	0
**OTU0798**	2	24	6	0	0	1	0	0	0	0	2	0	0	0	0	0	1	0
**OTU0799**	0	0	1	0	0	0	0	0	0	0	0	0	0	0	0	0	0	0
**OTU0800**	3	0	5	0	0	0	0	0	0	0	0	0	1	0	0	0	0	0
**OTU0801**	2	20	3	1	0	0	2	5	1	1	7	2	0	0	0	0	0	0
**OTU0802**	3	3	0	1	0	0	1	1	0	0	0	1	0	1	0	1	1	1
**OTU0803**	0	0	0	0	0	0	0	1	0	0	1	0	0	0	0	1	0	0
**OTU0804**	2	1	0	0	0	0	0	0	0	1	1	0	0	0	0	0	0	0
**OTU0805**	0	0	1	0	0	0	0	0	0	0	0	0	0	0	0	0	2	0
**OTU0806**	1	2	5	0	0	0	2	0	1	0	0	0	0	0	0	1	0	1
**OTU0807**	0	0	0	0	1	0	0	0	0	0	1	0	0	0	0	0	0	0
**OTU0808**	0	0	0	0	0	0	1	0	0	0	0	0	0	3	0	0	0	0
**OTU0809**	0	3	1	0	1	0	0	1	0	0	0	0	0	0	0	0	0	0
**OTU0810**	0	2	1	0	1	0	0	0	0	4	0	0	0	1	0	0	0	0
**OTU0811**	1	0	2	0	1	0	0	2	2	2	0	5	0	1	0	0	0	2
**OTU0812**	0	0	0	0	0	0	0	0	0	0	2	0	0	1	0	0	3	0
**OTU0813**	0	0	0	0	0	0	0	0	0	0	0	0	2	0	0	0	1	0
**OTU0814**	1	0	16	0	0	0	0	0	0	0	0	0	0	0	0	0	0	0

**OTU0815**	3	3	2	0	0	0	1	0	0	0	3	1	0	0	0	0	0	0
**OTU0816**	0	1	0	0	0	0	0	0	0	0	3	0	0	0	0	0	0	0
**OTU0817**	0	1	0	0	0	0	0	0	0	0	2	0	0	0	0	1	0	2
**OTU0818**	3	1	8	2	0	2	0	6	0	8	25	8	1	27	1	11	13	2
**OTU0819**	0	8	5	0	0	0	0	1	0	0	1	1	0	0	0	0	0	0
**OTU0820**	0	0	2	1	0	0	0	0	0	0	0	1	0	0	0	0	0	0
**OTU0821**	12	18	41	11	8	2	15	6	8	24	39	15	3	14	5	19	29	14
**OTU0822**	1	0	4	1	0	0	1	1	2	5	7	2	0	8	0	4	3	6
**OTU0823**	0	0	0	0	0	0	0	0	0	1	0	0	0	0	0	0	0	0
**OTU0824**	0	0	0	0	0	0	0	0	0	0	0	1	0	1	0	0	0	0
**OTU0825**	0	1	0	0	0	0	0	0	0	0	1	0	0	0	0	2	0	0
**OTU0826**	0	0	5	0	0	0	0	0	0	0	0	0	0	0	0	0	0	1
**OTU0827**	2	0	0	0	0	0	0	0	0	0	0	1	0	0	0	0	0	1
**OTU0828**	0	1	0	0	0	0	0	0	1	0	0	0	0	1	0	0	1	2
**OTU0829**	1	1	1	0	0	0	0	0	1	0	0	0	0	1	1	0	0	0
**OTU0830**	1	0	0	0	0	0	0	0	0	0	0	0	0	0	0	0	1	0
**OTU0831**	1	0	0	0	1	0	0	0	0	0	0	0	0	0	0	0	0	0
**OTU0832**	0	0	0	0	0	0	0	0	0	0	0	0	0	1	0	0	1	0
**OTU0833**	0	6	0	1	1	0	2	0	0	1	0	0	0	1	0	2	2	1
**OTU0834**	0	0	0	0	0	0	0	1	0	0	0	0	0	1	0	0	0	0
**OTU0835**	0	0	6	0	0	0	0	0	1	0	0	0	0	0	0	0	0	1
**OTU0836**	0	1	0	0	0	0	0	0	0	1	0	2	0	2	0	0	0	1
**OTU0837**	0	0	0	0	0	0	1	0	0	0	1	0	0	0	0	0	0	0
**OTU0838**	0	1	0	0	0	0	0	0	0	1	0	0	0	0	0	0	0	0
**OTU0839**	0	1	6	0	0	0	0	0	0	2	9	0	0	1	0	3	0	2
**OTU0840**	0	0	0	0	0	0	0	0	0	0	0	0	0	1	0	0	0	0
**OTU0841**	3	6	6	1	2	1	2	0	1	4	8	4	1	3	1	2	2	0
**OTU0842**	0	0	0	0	0	0	0	0	0	1	0	0	0	0	0	1	0	0
**OTU0843**	0	0	1	0	0	0	1	0	0	0	0	0	0	0	0	0	0	0
**OTU0844**	0	0	0	0	0	0	0	1	0	0	0	1	0	0	0	0	0	0
**OTU0845**	0	1	0	0	0	0	0	0	0	1	0	0	0	0	0	0	0	0
**OTU0846**	19	170	65	0	4	0	1	0	3	6	6	0	0	2	0	1	0	1
**OTU0847**	0	2	0	2	0	0	0	0	0	3	3	0	0	0	0	0	0	0
**OTU0848**	15	31	17	1	3	4	2	4	1	16	25	14	1	5	0	4	8	1
**OTU0849**	0	0	1	0	0	0	4	0	2	1	1	0	0	2	0	3	2	2
**OTU0850**	0	0	0	0	0	0	1	1	0	0	0	0	0	0	0	1	0	1
**OTU0851**	0	1	2	0	0	0	1	1	0	1	0	0	0	0	0	2	0	0
**OTU0852**	0	0	0	0	1	0	0	0	0	0	2	0	1	1	0	0	0	0
**OTU0853**	7	11	17	0	5	3	1	1	1	12	17	24	1	0	0	0	2	0

**OTU0854**	3	2	3	0	0	0	1	0	0	0	0	2	1	2	0	2	1	2
**OTU0855**	2	2	4	0	0	0	5	1	1	1	2	4	1	1	0	0	2	0
**OTU0856**	0	0	1	0	0	0	0	0	0	0	1	0	0	0	0	0	1	0
**OTU0857**	1272	3342	2893	654	495	227	931	674	373	2009	3284	971	294	841	221	882	883	836
**OTU0858**	2	2	1	0	0	0	4	0	0	0	0	0	0	0	1	0	0	0
**OTU0859**	19	26	26	10	10	3	118	60	41	26	40	12	2	9	4	86	104	98
**OTU0860**	1	0	4	0	0	0	0	0	0	0	0	0	0	0	0	0	0	0
**OTU0861**	2	3	1	1	0	1	0	2	3	0	5	2	0	0	0	0	0	0
**OTU0862**	0	0	0	0	0	0	0	0	1	0	2	0	0	0	0	0	0	0
**OTU0863**	4	0	3	0	0	1	536	2	0	1	2	0	1	2	0	0	0	0
**OTU0864**	3	1	1	2	1	1	1	0	0	8	5	2	0	1	0	2	4	3
**OTU0865**	1	0	0	0	0	0	0	0	0	0	0	0	0	0	0	0	0	0
**OTU0866**	0	0	0	0	0	0	0	0	0	0	1	0	0	0	1	0	0	0
**OTU0867**	0	0	1	0	0	0	0	0	0	0	1	0	0	0	0	0	0	0
**OTU0868**	0	1	0	0	1	0	0	0	0	0	0	0	0	0	0	0	0	0
**OTU0869**	0	0	0	0	0	0	0	1	0	1	0	0	0	0	0	0	0	0
**OTU0870**	0	0	0	0	0	0	0	0	0	1	0	0	0	0	0	0	1	0
**OTU0871**	0	0	5	3	0	0	5	0	0	0	3	0	0	0	0	0	0	0
**OTU0872**	0	0	2	1	0	1	0	0	0	0	1	0	0	1	0	0	0	0
**OTU0873**	0	0	1	0	0	0	0	0	0	0	2	0	0	0	0	0	0	0
**OTU0874**	1	0	0	0	0	0	0	1	0	0	0	0	0	0	0	0	0	0
**OTU0875**	0	0	0	0	0	0	0	1	0	0	0	0	0	0	0	0	2	0
**OTU0876**	0	0	0	1	0	0	0	0	0	0	0	0	0	0	5	0	0	0
**OTU0877**	15	34	21	6	6	1	11	6	8	18	53	33	4	8	3	60	22	43
**OTU0878**	1	0	0	0	0	0	0	0	0	0	0	0	0	0	0	0	2	0
**OTU0879**	219	335	382	126	90	30	213	94	70	312	504	192	69	162	28	150	201	141
**OTU0880**	0	1	0	0	0	0	0	0	0	0	1	0	0	0	0	0	0	0
**OTU0881**	1	0	0	0	1	0	1	0	0	0	0	0	0	0	0	0	0	0
**OTU0882**	12	19	267	3	5	6	10	11	12	95	469	16	44	530	181	8	19	4
**OTU0883**	0	0	0	0	0	0	0	0	0	0	1	0	0	0	0	0	0	0
**OTU0884**	2	6	10	4	1	0	15	4	2	9	10	3	2	2	3	20	14	16
**OTU0885**	1	3	0	0	0	0	0	0	0	0	0	0	0	0	0	0	0	0
**OTU0886**	0	0	0	0	0	0	0	0	0	1	0	0	0	0	0	0	0	0
**OTU0887**	0	0	0	0	0	0	0	0	0	0	1	0	0	0	0	0	0	0
**OTU0888**	0	2	2	0	0	0	1	0	1	1	0	0	0	1	0	0	1	0
**OTU0889**	0	2	0	1	0	1	2	2	2	0	5	3	0	1	0	2	1	4
**OTU0890**	4	0	0	0	0	0	0	0	0	5	0	2	1	0	0	0	0	0
**OTU0891**	2	0	1	0	0	0	1	0	0	1	0	0	1	0	0	0	0	0
**OTU0892**	8	193	35	0	0	0	1	0	1	0	16	0	0	0	0	0	1	1

**OTU0893**	0	0	0	0	1	0	0	1	0	0	0	0	0	0	0	0	0	0
**OTU0894**	0	0	0	0	0	0	0	0	0	0	0	0	0	0	0	4	0	0
**OTU0895**	20	25	10	2	0	2	5	8	0	25	55	24	3	0	0	77	10	47
**OTU0896**	0	1	1	0	0	0	0	0	0	0	0	0	0	0	0	0	0	0
**OTU0897**	4	4	17	3	2	0	2	0	0	0	0	1	0	7	1	0	0	0
**OTU0898**	0	1	0	1	0	0	0	0	0	3	0	5	0	0	0	1	0	1
**OTU0899**	0	0	1	0	0	1	1	0	0	0	2	0	0	0	0	1	0	0
**OTU0900**	1	0	0	0	0	0	0	0	0	0	2	0	0	0	0	1	0	1
**OTU0901**	1	0	1	0	1	0	0	0	0	0	0	0	0	0	0	0	0	0
**OTU0902**	0	0	0	0	0	0	0	0	0	1	0	0	0	0	0	0	0	0
**OTU0903**	0	3	1	0	0	0	1	0	1	0	0	1	1	0	0	1	3	2
**OTU0904**	0	3	3	0	0	0	0	0	0	0	0	0	0	0	0	0	0	0
**OTU0905**	0	0	0	0	0	0	1	1	0	3	1	0	0	0	0	0	1	0
**OTU0906**	0	7	0	0	0	1	0	0	0	4	3	0	0	0	3	0	0	0
**OTU0907**	0	0	0	0	0	0	0	0	1	0	2	0	0	0	0	0	0	0
**OTU0908**	2	0	0	1	0	0	0	0	0	3	3	1	0	2	0	0	1	1
**OTU0909**	0	2	0	0	1	0	1	2	0	0	1	2	0	0	0	0	0	0
**OTU0910**	0	0	1	0	0	0	0	0	0	0	1	0	0	1	1	0	0	0
**OTU0911**	1	0	0	0	1	0	1	0	0	1	7	0	0	0	0	0	1	0
**OTU0912**	5	37	19	0	0	0	1	0	0	8	20	4	0	1	0	0	0	1
**OTU0913**	1	5	1	0	0	0	0	0	0	0	0	0	0	0	0	0	0	0
**OTU0914**	0	0	0	0	0	0	0	0	0	0	1	0	0	0	0	0	1	0
**OTU0915**	3	1	3	3	0	0	0	1	0	6	8	0	0	6	0	1	0	2
**OTU0916**	7	4	10	2	2	0	1	0	1	5	9	1	0	1	0	5	5	3
**OTU0917**	0	0	0	0	0	0	0	0	0	0	1	0	1	0	0	0	0	0
**OTU0918**	0	0	0	0	0	0	7	0	3	0	0	0	0	0	0	0	0	3
**OTU0919**	0	2	0	0	0	0	0	0	0	0	6	0	0	0	0	0	0	0
**OTU0920**	1	1	1	0	1	0	2	0	0	0	4	0	0	0	1	4	2	4
**OTU0921**	2	0	3	1	1	0	1	0	0	1	2	1	1	0	2	1	2	1
**OTU0922**	22	35	20	2	10	6	16	19	5	15	30	19	10	2	3	2	7	0
**OTU0923**	3	1	0	0	0	0	1	1	1	0	2	1	0	0	0	2	1	3
**OTU0924**	0	2	3	0	1	0	1	0	0	0	1	1	0	0	0	1	0	0
**OTU0925**	1	2	3	1	0	0	1	0	0	0	1	0	1	0	0	0	1	0
**OTU0926**	0	0	0	0	4	0	0	0	0	0	0	1	0	0	0	0	0	0
**OTU0927**	0	1	0	1	0	0	0	0	0	0	0	0	0	1	0	0	0	0
**OTU0928**	0	0	0	0	0	0	0	0	0	0	0	0	1	1	0	0	0	0
**OTU0929**	1	0	6	0	0	0	1	0	1	1	4	0	1	1	0	1	4	4
**OTU0930**	0	1	0	0	0	0	6	0	0	1	1	0	0	0	0	0	0	0
**OTU0931**	0	0	1	0	0	0	0	0	0	0	7	0	0	0	0	0	0	0

**OTU0932**	49	134	108	29	18	18	55	24	26	45	90	45	15	19	3	12	16	13
**OTU0933**	0	0	7	0	0	0	0	0	0	0	2	0	0	0	0	0	0	0
**OTU0934**	12	1	9	0	0	1	12	54	4	8	4	16	1	2	2	12	3	1
**OTU0935**	1	0	1	0	0	0	1	1	0	0	1	0	0	0	0	1	0	2
**OTU0936**	1	0	0	0	0	0	0	0	0	1	0	0	0	0	0	0	0	0
**OTU0937**	0	0	0	0	0	0	0	0	0	0	2	0	1	0	0	0	0	0
**OTU0938**	0	8	0	2	1	0	0	0	0	2	6	2	0	0	0	0	0	0
**OTU0939**	0	1	6	1	1	0	0	1	0	2	3	0	0	0	0	0	4	1
**OTU0940**	0	0	1	0	0	0	0	0	0	0	0	0	0	0	0	0	1	0
**OTU0941**	1	1	0	0	0	0	0	0	0	0	0	0	0	0	0	1	0	0
**OTU0942**	0	0	0	0	1	0	0	0	0	0	0	3	0	0	0	0	0	0
**OTU0943**	0	0	0	0	0	0	0	0	0	2	0	0	0	0	0	1	0	0
**OTU0944**	0	0	0	0	1	0	1	0	0	1	0	0	0	0	0	0	1	0
**OTU0945**	0	0	0	0	0	0	0	0	0	0	0	0	0	0	0	0	1	0
**OTU0946**	0	0	1	0	1	0	0	0	0	0	0	0	1	1	0	0	0	0
**OTU0947**	2862	4508	4918	1380	1295	555	2425	1507	1035	3859	5872	2455	804	1776	489	1828	2368	1678
**OTU0948**	0	0	0	0	0	0	0	0	0	0	0	0	0	0	1	0	0	0
**OTU0949**	0	0	1	0	0	0	0	0	0	0	0	2	0	1	0	0	0	0
**OTU0950**	0	0	0	0	0	0	0	0	0	2	1	0	0	0	0	0	1	0
**OTU0951**	0	0	1	0	0	0	1	1	0	0	0	0	0	0	0	0	0	1
**OTU0952**	0	1	1	0	0	1	0	0	0	0	1	0	0	1	0	1	1	0
**OTU0953**	1	2	0	0	0	0	2	0	0	0	0	0	0	0	0	0	0	0
**OTU0954**	32	48	22	1	4	9	16	15	7	14	19	13	3	3	0	0	2	5
**OTU0955**	0	1	2	0	0	0	0	0	0	1	1	0	0	0	0	0	0	0
**OTU0956**	0	3	0	0	0	0	0	0	0	0	0	0	0	0	0	0	1	0
**OTU0957**	0	0	1	0	0	0	0	0	0	2	0	0	0	0	0	0	0	0
**OTU0958**	0	0	0	0	0	0	1	0	1	0	0	0	0	0	0	0	0	0
**OTU0959**	85	142	170	54	47	19	102	74	37	150	194	74	47	87	24	92	135	91
**OTU0960**	0	0	0	0	0	0	0	2	1	0	0	0	0	0	0	0	0	0
**OTU0961**	1	2	6	0	2	0	0	0	0	0	8	1	0	0	0	0	0	0
**OTU0962**	22	67	42	8	9	0	22	6	6	28	90	26	7	11	5	22	8	6
**OTU0963**	1	1	0	0	0	0	0	0	0	0	0	0	0	0	0	0	0	0
**OTU0964**	0	0	0	1	0	0	0	0	2	0	1	0	2	0	0	0	0	2
**OTU0965**	1	2	4	0	0	0	1	0	0	2	1	3	2	1	0	0	0	1
**OTU0966**	0	0	0	0	0	0	0	0	0	1	1	0	0	0	0	1	1	0
**OTU0967**	0	1	1	0	0	0	0	0	0	0	2	0	0	1	0	0	1	0
**OTU0968**	3	2	1	0	0	0	1	1	0	0	3	3	0	0	0	0	1	0
**OTU0969**	1	4	2	0	0	1	0	2	0	0	2	1	0	1	0	0	1	1
**OTU0970**	1	2	1	1	1	0	0	1	0	0	2	2	0	0	0	1	0	1

**OTU0971**	1	0	0	0	0	0	0	0	0	1	2	0	1	0	1	0	5	0
**OTU0972**	1	1	0	0	1	0	0	0	0	0	0	0	0	0	1	0	0	0
**OTU0973**	0	0	1	0	0	0	0	0	3	0	0	0	0	0	0	0	0	0
**OTU0974**	5	9	7	3	1	0	3	2	0	3	8	5	3	0	11	3	4	4
**OTU0975**	1	2	0	2	0	0	0	0	0	0	0	0	0	0	0	0	0	0
**OTU0976**	0	0	1	0	0	0	0	0	0	0	0	0	0	0	0	0	0	0
**OTU0977**	0	0	0	0	0	0	0	1	1	0	0	0	0	0	0	0	0	0
**OTU0978**	0	2	0	0	0	0	0	0	0	0	0	0	0	1	0	0	0	0
**OTU0979**	0	0	0	0	0	0	0	0	0	0	1	0	0	0	0	0	1	0
**OTU0980**	179	223	279	86	90	41	180	105	60	228	326	128	61	159	58	149	140	134
**OTU0981**	11	3	6	2	1	1	3	1	0	4	17	5	0	1	2	1	1	0
**OTU0982**	1	3	5	0	0	0	1	0	0	2	4	1	0	0	0	0	2	0
**OTU0983**	9	11	26	0	4	2	0	0	2	0	25	5	0	0	0	0	0	2
**OTU0984**	0	0	0	0	0	0	0	0	0	1	0	0	0	0	0	0	0	1
**OTU0985**	1	0	0	1	1	0	2	1	0	2	2	8	0	0	0	5	0	2
**OTU0986**	0	4	0	0	0	0	0	0	0	0	0	0	0	0	0	0	0	0
**OTU0987**	1	6	5	0	1	1	0	0	3	3	4	4	0	4	0	0	0	1
**OTU0988**	0	1	0	0	0	0	1	0	0	0	0	0	0	0	0	0	0	0
**OTU0989**	0	0	0	0	0	0	0	0	0	0	1	0	0	0	0	1	0	0
**OTU0990**	0	0	1	0	0	0	0	0	0	0	2	0	0	0	1	0	1	0
**OTU0991**	0	3	0	0	0	1	1	0	0	0	0	0	0	0	0	0	0	0
**OTU0992**	4	3	1	0	2	0	4	2	2	0	3	2	0	4	1	0	0	0
**OTU0993**	0	1	1	0	0	0	0	0	0	0	1	0	0	0	0	0	0	0
**OTU0994**	0	0	1	0	0	0	1	0	0	1	2	1	2	1	2	2	2	0
**OTU0995**	0	0	0	0	0	0	0	0	1	0	0	0	0	0	0	0	0	0
**OTU0996**	0	0	1	0	0	0	0	0	0	0	0	1	0	0	0	0	0	0
**OTU0997**	23	30	4	2	6	3	9	6	13	1	1	26	0	1	0	0	0	0
**OTU0998**	0	1	0	0	0	0	1	2	0	1	0	3	0	1	0	0	1	0
**OTU0999**	0	0	0	0	0	0	0	0	0	0	0	1	0	0	1	0	0	0
**OTU1000**	1	1	1	0	2	0	2	0	0	0	0	1	0	0	0	1	1	0
**OTU1001**	378	700	685	214	165	56	364	208	161	735	885	317	100	293	60	311	342	250
**OTU1002**	0	0	7	0	0	0	1	0	0	3	0	0	0	0	0	0	0	0
**OTU1003**	0	0	1	0	0	1	0	0	0	0	0	0	0	0	0	0	0	0
**OTU1004**	146	189	125	30	37	36	84	78	45	57	123	143	35	36	9	11	30	20
**OTU1005**	0	1	0	1	0	0	0	1	0	0	0	2	0	0	0	1	0	0
**OTU1006**	0	0	1	0	0	0	0	0	0	1	0	0	0	0	0	2	0	0
**OTU1007**	62	107	176	21	16	10	50	33	51	112	139	68	11	73	11	130	40	107
**OTU1008**	0	0	0	0	1	0	0	0	0	1	0	0	0	0	0	0	0	0
**OTU1009**	0	0	0	0	0	0	0	0	0	0	3	4	0	0	0	0	1	0

**OTU1010**	1	5	0	0	0	0	0	0	0	0	0	0	0	0	0	0	0	0
**OTU1011**	1	1	1	0	0	0	1	0	0	0	0	0	0	1	1	0	0	0
**OTU1012**	1	0	1	0	0	0	0	0	0	0	2	0	0	0	0	0	2	0
**OTU1013**	0	0	0	0	0	0	0	0	0	0	3	2	0	1	0	0	0	0
**OTU1014**	0	5	2	1	1	1	1	0	1	2	4	1	1	0	3	3	0	2
**OTU1015**	0	0	1	0	0	0	0	1	0	0	0	0	0	2	0	0	0	0
**OTU1016**	0	0	0	0	0	0	5	0	1	0	1	0	0	0	0	2	0	0
**OTU1017**	5	7	4	0	1	0	5	2	3	3	8	1	0	2	2	2	3	2
**OTU1018**	17	40	24	12	6	3	9	2	4	31	28	4	0	7	0	6	8	2
**OTU1019**	0	2	0	1	0	2	0	0	0	3	1	0	0	0	0	0	0	8
**OTU1020**	0	0	0	0	1	0	0	0	0	0	0	1	0	1	0	1	0	0
**OTU1021**	11	0	0	0	0	0	0	0	1	0	8	2	0	0	0	0	0	0
**OTU1022**	0	0	0	0	0	0	0	0	0	2	0	2	0	0	0	0	0	0
**OTU1023**	21	55	57	16	18	7	16	11	17	29	59	23	17	15	3	19	18	13
**OTU1024**	0	2	1	0	0	0	0	0	0	0	4	0	0	0	0	0	0	0
**OTU1025**	0	2	0	1	0	0	0	1	0	1	1	0	0	3	0	0	0	0
**OTU1026**	0	0	0	0	0	0	0	0	0	0	1	0	0	0	0	1	1	0
**OTU1027**	4	14	9	6	0	1	6	2	0	12	9	5	0	3	0	3	3	2
**OTU1028**	0	0	1	0	0	0	0	0	0	0	0	0	0	1	0	0	0	0
**OTU1029**	511	751	779	256	198	112	503	270	161	616	943	363	162	350	177	250	365	367
**OTU1030**	278	343	301	83	87	78	230	118	61	155	360	200	77	116	31	84	102	72
**OTU1031**	7	19	28	4	3	5	11	8	13	19	42	5	1	11	1	9	7	12
**OTU1032**	0	1	0	2	0	0	3	0	0	1	1	1	0	0	0	1	0	2
**OTU1033**	0	1	1	0	0	0	0	0	0	0	0	0	0	0	0	0	0	0
**OTU1034**	0	0	0	0	0	0	1	0	0	1	1	1	0	0	0	1	0	0
**OTU1035**	0	0	0	0	0	0	0	0	0	0	0	1	0	0	0	0	0	0
**OTU1036**	0	0	0	0	0	0	0	1	0	0	1	0	0	0	0	0	0	0
**OTU1037**	0	0	0	0	0	0	0	0	0	0	1	1	0	0	0	0	0	0
**OTU1038**	0	0	0	0	0	0	0	0	0	0	1	0	0	0	0	0	0	0
**OTU1039**	1	0	0	0	0	0	0	0	0	0	0	0	0	0	0	0	1	0
**OTU1040**	2	0	1	0	0	0	0	0	0	1	1	0	0	1	0	1	0	0
**OTU1041**	0	0	1	0	1	0	0	0	0	0	3	0	0	0	0	0	0	1
**OTU1042**	4	9	8	2	2	0	0	0	2	5	6	0	2	2	0	1	3	2
**OTU1043**	0	0	1	0	0	0	0	0	0	0	1	0	0	0	0	0	0	0
**OTU1044**	0	0	0	0	0	0	0	0	0	0	1	0	0	0	0	0	0	0
**OTU1045**	0	0	1	1	0	0	0	0	0	1	0	0	0	1	1	0	0	1
**OTU1046**	0	0	0	0	0	0	0	0	0	0	0	2	0	0	0	0	0	0
**OTU1047**	39	48	76	30	21	9	45	29	16	68	95	36	22	48	14	45	56	38
**OTU1048**	4	6	4	0	0	1	0	0	0	2	2	3	0	3	0	0	1	1

**OTU1049**	0	0	0	0	1	0	0	0	0	0	0	0	0	0	1	0	0	0
**OTU1050**	0	0	0	0	0	0	0	0	0	0	0	0	0	0	1	0	1	0
**OTU1051**	0	1	2	0	0	0	0	0	0	1	1	0	0	0	0	0	0	0
**OTU1052**	0	0	2	0	0	0	0	1	0	0	0	0	0	0	0	0	0	0
**OTU1053**	1	1	1	2	0	0	0	0	0	0	4	0	0	0	0	0	2	1
**OTU1054**	0	0	5	0	0	0	0	0	0	0	0	0	0	0	1	0	0	0
**OTU1055**	0	0	0	0	0	0	2	4	2	0	0	0	0	0	0	0	0	0
**OTU1056**	0	2	0	0	0	0	0	0	0	0	0	0	0	0	0	1	0	0
**OTU1057**	0	0	1	0	0	0	1	0	0	1	0	1	0	0	0	0	0	0
**OTU1058**	0	0	1	0	0	0	0	0	0	0	0	0	0	0	0	0	1	0
**OTU1059**	0	0	2	0	1	1	0	0	0	0	0	0	0	0	1	0	0	0
**OTU1060**	0	24	6	1	0	0	0	0	0	0	0	0	0	1	0	2	0	3
**OTU1061**	0	0	0	0	0	0	0	0	0	0	0	0	0	0	0	0	0	1
**OTU1062**	1	7	5	2	0	1	2	1	0	1	3	3	0	2	0	2	0	0
**OTU1063**	7	3	118	0	3	4	15	6	3	9	12	5	12	3	33	3	0	0
**OTU1064**	1	2	0	0	0	0	1	0	2	0	0	0	0	0	0	0	0	0
**OTU1065**	4	1	0	0	0	0	0	0	0	2	0	2	0	0	0	0	0	2
**OTU1066**	0	0	0	0	0	0	0	0	0	0	1	0	0	0	0	0	0	2
**OTU1067**	0	0	0	0	0	0	1	1	0	1	0	0	0	0	1	0	1	0
**OTU1068**	0	3	0	0	0	0	0	0	0	1	0	0	0	0	0	0	0	0
**OTU1069**	0	3	1	0	0	1	2	0	0	9	5	0	0	5	0	0	1	3
**OTU1070**	2	10	5	2	3	1	7	2	0	3	5	3	1	1	0	7	1	4
**OTU1071**	0	1	0	0	0	0	0	0	0	0	0	0	0	0	0	0	0	0
**OTU1072**	0	1	2	0	0	0	1	0	0	2	0	0	0	0	2	0	0	0
**OTU1073**	0	0	1	0	0	0	0	0	1	0	0	0	0	0	0	0	0	0
**OTU1074**	21	58	36	6	1	1	8	5	2	22	31	15	1	7	5	7	9	9
**OTU1075**	0	0	0	0	0	0	0	0	0	0	0	0	0	2	0	2	0	0
**OTU1076**	0	0	0	0	0	0	1	2	0	0	0	0	0	0	0	0	0	0
**OTU1077**	0	2	0	0	0	0	1	0	0	1	2	1	0	0	0	0	0	3
**OTU1078**	2	8	111	1	2	2	2	6	0	24	70	2	132	94	97	3	4	3
**OTU1079**	0	0	0	1	0	1	0	0	0	0	0	0	0	1	0	1	1	0
**OTU1080**	4	1	2	0	0	0	3	1	0	3	4	3	1	1	1	2	0	1
**OTU1081**	3	1	0	0	0	0	13	0	0	1	0	0	0	0	0	0	0	0
**OTU1082**	5	6	4	1	0	2	42	94	14	3	2	4	1	1	9	1	1	2
**OTU1083**	6	24	20	1	1	2	3	1	3	4	1	7	1	0	0	0	0	0
**OTU1084**	14	225	70	2	0	0	4	0	1	1	28	3	2	2	1	4	1	4
**OTU1085**	0	0	0	0	0	0	0	0	0	0	1	0	0	0	0	0	0	0
**OTU1086**	0	3	3	0	0	0	0	0	0	0	2	5	1	0	0	5	0	2
**OTU1087**	2	2	1	0	1	0	0	0	1	0	0	1	0	0	1	0	0	0

**OTU1088**	0	0	0	0	0	0	0	0	0	1	1	0	0	0	0	0	0	0
**OTU1089**	0	1	0	0	0	1	0	0	0	0	0	0	0	0	0	0	0	0
**OTU1090**	51	150	137	11	10	4	25	23	7	85	85	39	8	6	2	27	33	16
**OTU1091**	18	62	31	0	2	1	7	3	3	8	22	5	3	5	0	2	5	6
**OTU1092**	0	1	0	0	0	0	0	1	0	0	0	0	1	0	0	1	0	0
**OTU1093**	0	0	0	0	0	0	2	0	0	1	1	0	0	0	0	0	0	0
**OTU1094**	0	0	1	1	0	0	0	1	0	1	0	0	0	0	0	0	0	0
**OTU1095**	0	0	0	1	0	0	0	0	0	0	0	2	0	0	0	0	0	0
**OTU1096**	1	1	1	0	0	0	0	0	1	1	3	0	0	0	0	0	0	0
**OTU1097**	16	7	17	3	0	5	7	4	2	19	14	3	3	7	1	7	11	3
**OTU1098**	0	1	1	0	1	0	2	3	1	0	0	0	0	0	1	3	2	1
**OTU1099**	0	1	0	0	0	0	0	2	0	0	0	0	0	0	0	3	0	0
**OTU1100**	0	2	0	0	0	0	0	0	0	0	0	0	0	0	0	1	0	0
**OTU1101**	9	13	17	3	3	0	5	2	10	15	25	14	0	5	3	12	3	13
**OTU1102**	3	4	6	1	1	1	1	0	0	2	4	0	0	0	0	2	4	1
**OTU1103**	0	0	0	0	0	0	1	0	0	0	1	0	0	0	0	0	0	0
**OTU1104**	2113	2373	3887	90	913	590	208	143	68	2659	3797	4160	512	279	466	74	119	44
**OTU1105**	0	0	0	0	0	0	1	0	0	1	1	0	0	1	0	0	0	0
**OTU1106**	1	0	0	0	0	0	0	1	0	0	0	0	0	0	0	1	0	0
**OTU1107**	0	0	0	1	0	0	0	0	0	1	0	0	0	0	0	2	1	0
**OTU1108**	0	0	1	0	0	0	0	0	0	1	0	0	0	0	0	0	0	0
**OTU1109**	0	0	0	0	0	0	0	0	0	0	2	0	0	0	0	1	0	0
**OTU1110**	0	0	0	0	1	0	1	0	0	0	0	0	0	0	0	0	0	0
**OTU1111**	3	0	0	0	2	1	4	3	2	0	18	3	0	17	4	13	2	1
**OTU1112**	0	0	0	1	0	0	0	0	0	0	0	0	0	0	0	0	0	1
**OTU1113**	0	0	0	0	0	0	0	1	0	0	0	0	1	0	0	0	0	0
**OTU1114**	0	0	0	0	0	0	0	1	0	0	0	0	1	0	0	1	0	0
**OTU1115**	0	0	1	0	1	0	0	0	0	1	0	0	0	1	0	2	0	0
**OTU1116**	0	2	13	0	0	0	1	0	0	3	0	0	0	1	0	0	0	0
**OTU1117**	398	450	7941	30	105	96	459	339	317	1271	9230	449	6249	877	2245	39	61	35
**OTU1118**	0	0	1	9	2	0	3	12	4	4	11	2	4	1	2	0	5	11
**OTU1119**	0	0	0	0	0	0	0	1	0	1	0	0	0	0	0	0	0	0
**OTU1120**	9	24	81	13	15	1	28	16	8	60	62	22	2	28	4	32	33	19
**OTU1121**	0	1	0	0	0	0	0	0	0	0	0	0	0	0	0	0	0	0
**OTU1122**	0	0	0	0	0	0	0	0	0	1	0	0	0	0	0	0	0	0
**OTU1123**	0	0	1	0	0	0	0	0	0	2	0	0	0	0	0	0	0	0
**OTU1124**	0	0	0	0	0	0	0	0	0	3	0	1	0	0	0	0	0	0
**OTU1125**	0	0	0	0	0	0	0	0	0	0	0	0	0	0	1	0	0	1
**OTU1126**	0	0	0	0	0	0	1	0	0	1	1	0	0	2	0	0	1	0

**OTU1127**	2	1	2	0	2	1	2	0	0	6	4	0	1	1	2	3	5	3
**OTU1128**	0	0	0	0	1	0	0	0	0	0	0	1	0	0	0	0	0	0
**OTU1129**	11	23	11	5	4	1	8	4	2	16	15	4	0	1	1	5	7	3
**OTU1130**	0	3	0	0	0	0	0	0	0	0	4	0	0	0	0	0	2	0
**OTU1131**	127	257	330	73	49	37	120	102	55	300	243	96	38	104	43	184	130	91
**OTU1132**	1	0	0	0	0	0	0	0	0	1	17	0	1	0	0	0	0	0
**OTU1133**	0	0	1	0	0	0	0	0	0	0	1	0	0	0	0	0	0	0
**OTU1134**	1	6	3	2	0	0	2	0	0	0	7	1	1	1	1	0	1	2
**OTU1135**	1	1	0	0	0	0	1	0	0	0	2	0	0	2	1	0	0	0
**OTU1136**	0	1	1	0	0	0	0	0	0	0	0	0	0	0	0	0	0	0
**OTU1137**	108	179	179	70	57	28	116	61	35	179	268	94	35	91	32	86	107	95
**OTU1138**	34	31	22	2	5	9	20	13	9	9	22	29	8	2	5	2	3	3
**OTU1139**	0	0	0	0	0	0	0	0	0	0	0	0	0	1	0	3	2	1
**OTU1140**	0	0	0	0	0	0	0	1	0	0	1	0	1	1	0	0	0	2
**OTU1141**	1	4	3	0	0	0	0	0	0	0	2	0	0	0	0	0	0	0
**OTU1142**	0	6	0	0	0	0	1	0	1	0	3	6	0	0	0	0	0	0
**OTU1143**	1	0	0	1	0	1	0	1	2	5	0	0	0	0	0	1	0	1
**OTU1144**	0	0	0	0	0	0	0	0	0	0	0	0	0	0	7	0	0	0
**OTU1145**	37	70	70	8	15	9	30	23	8	45	43	24	8	11	9	15	18	8
**OTU1146**	5	0	0	0	0	0	0	0	0	0	0	3	0	0	0	0	0	0
**OTU1147**	1	0	1	0	0	0	1	0	0	0	3	0	0	0	0	0	0	0
**OTU1148**	0	0	0	0	0	0	0	0	0	0	4	0	0	0	0	0	0	1
**OTU1149**	0	2	0	0	0	0	0	0	0	2	4	2	1	2	1	1	3	1
**OTU1150**	6	18	10	7	6	5	19	5	6	19	17	14	3	18	3	6	10	4
**OTU1151**	0	0	0	0	0	0	0	0	0	0	0	1	0	0	0	1	0	0
**OTU1152**	0	1	1	2	0	0	0	1	0	3	0	1	1	0	1	3	0	1
**OTU1153**	0	3	0	0	0	0	0	1	0	0	0	0	0	0	0	0	0	0
**OTU1154**	0	0	0	0	0	0	0	1	0	1	0	0	0	0	0	0	0	0
**OTU1155**	0	0	0	0	0	0	1	0	0	0	1	0	0	0	0	0	0	0
**OTU1156**	106	178	156	43	50	25	105	56	17	103	167	75	25	64	14	50	69	49
**OTU1157**	2	9	2	0	1	0	2	1	0	2	7	0	0	0	2	4	2	3
**OTU1158**	0	0	0	0	0	0	0	0	0	0	0	0	0	0	1	0	0	0
**OTU1159**	0	0	1	0	0	0	0	0	0	1	0	0	0	0	0	0	0	0
**OTU1160**	0	1	2	0	0	0	0	0	1	2	2	0	0	0	1	0	1	0
**OTU1161**	1	2	4	1	1	0	0	2	2	1	2	3	0	1	0	2	1	1
**OTU1162**	233	328	378	134	85	41	242	139	91	376	502	208	49	167	44	220	257	175
**OTU1163**	5	7	6	0	0	1	0	2	0	7	11	0	0	4	0	2	1	1
**OTU1164**	0	0	0	1	1	0	0	0	0	0	0	0	0	0	0	0	0	0
**OTU1165**	2	1	1	1	0	0	0	0	0	0	3	1	0	0	0	2	1	0

**OTU1166**	0	0	0	0	0	0	0	0	1	0	0	0	0	0	0	1	0	0
**OTU1167**	0	0	0	0	0	0	0	0	0	1	0	0	1	0	0	0	0	0
**OTU1168**	0	1	4	0	0	0	0	0	0	0	3	0	0	1	0	0	0	0
**OTU1169**	4	0	4	1	0	0	0	0	3	2	13	0	0	0	0	2	1	0
**OTU1170**	1	9	0	0	0	0	0	0	0	1	0	0	0	0	0	0	0	0
**OTU1171**	0	9	1	0	0	0	1	0	0	0	10	1	0	0	0	0	0	0
**OTU1172**	1	0	3	0	0	0	0	0	0	0	2	1	1	0	0	0	0	1
**OTU1173**	0	0	0	0	0	0	1	0	2	0	0	0	0	0	1	0	0	0
**OTU1174**	0	1	0	0	0	0	1	0	0	1	1	1	0	1	0	0	0	1
**OTU1175**	0	8	3	1	0	0	0	0	0	0	1	0	0	0	0	0	0	0
**OTU1176**	0	0	1	0	0	0	0	0	0	0	1	0	1	0	0	0	0	0
**OTU1177**	1	1	0	0	0	0	0	0	0	0	4	0	0	0	0	0	0	0
**OTU1178**	0	1	2	0	0	0	0	0	0	0	1	0	2	4	4	0	0	0
**OTU1179**	1	4	2	2	2	0	1	0	3	7	13	3	0	2	0	2	1	1
**OTU1180**	2	0	0	0	0	0	2	0	0	1	0	0	0	0	2	0	1	0
**OTU1181**	4	104	17	0	0	0	0	1	0	0	12	0	0	0	3	0	0	0
**OTU1182**	10	17	11	1	5	6	11	8	5	4	17	13	3	1	1	3	2	2
**OTU1183**	15	9	0	0	0	1	43	2	1	1	4	3	2	1	2	1	0	2
**OTU1184**	0	2	0	0	0	0	0	0	0	0	0	0	0	0	0	0	0	0
**OTU1185**	1	0	1	0	0	0	0	0	0	1	0	0	0	0	0	0	1	0
**OTU1186**	0	0	0	0	0	0	0	0	0	0	0	0	0	0	0	0	0	1
**OTU1187**	0	0	0	0	0	0	0	0	0	0	1	1	0	0	0	0	0	0
**OTU1188**	0	0	1	0	0	0	0	0	0	0	3	0	0	0	0	0	0	0
**OTU1189**	0	0	1	0	0	0	1	0	0	0	1	0	0	0	0	0	0	0
**OTU1190**	0	1	1	0	0	0	1	0	0	0	2	0	1	0	0	0	0	0
**OTU1191**	0	0	0	1	0	0	0	0	0	0	0	0	0	0	1	0	0	0
**OTU1192**	19	38	51	11	14	24	36	11	4	48	59	39	4	11	4	54	11	44
**OTU1193**	0	1	0	1	0	0	0	0	0	1	0	0	0	0	0	0	0	0
**OTU1194**	1	20	17	0	0	0	0	0	0	0	0	0	0	0	0	0	0	0
**OTU1195**	4	4	8	0	2	1	2	0	1	0	1	1	0	0	6	0	1	2
**OTU1196**	20	42	56	14	0	6	40	10	8	26	64	11	6	27	6	17	24	9
**OTU1197**	0	0	1	0	0	0	0	0	0	3	0	0	0	1	0	0	0	0
**OTU1198**	0	0	0	1	0	0	0	0	0	0	1	0	0	0	0	0	0	0
**OTU1199**	1	1	0	0	0	0	2	1	0	0	0	0	1	0	0	1	1	0
**OTU1200**	0	0	0	1	0	0	0	0	0	1	0	0	0	1	0	1	2	0
**OTU1201**	0	1	2	0	0	0	0	0	0	0	0	0	0	0	0	0	0	0
**OTU1202**	73	117	178	14	26	7	53	22	16	145	106	42	13	28	17	57	57	29
**OTU1203**	1	2	0	0	1	0	0	0	0	0	0	0	0	0	0	2	0	0
**OTU1204**	0	0	0	0	0	0	2	0	1	0	0	0	0	0	0	1	0	0

**OTU1205**	16	14	13	1	2	2	7	4	3	4	11	7	6	1	1	1	0	2
**OTU1206**	0	0	1	0	0	0	0	0	0	0	0	0	0	0	0	0	0	0
**OTU1207**	8	31	31	16	11	6	29	16	11	41	44	5	7	26	3	37	49	37
**OTU1208**	0	7	0	1	1	0	0	0	4	0	0	1	0	2	0	0	1	0
**OTU1209**	0	2	0	0	0	0	0	0	0	0	4	1	0	0	0	1	0	0
**OTU1210**	0	0	0	0	0	0	0	0	0	0	0	2	0	0	0	1	0	0
**OTU1211**	2	2	2	0	0	1	0	0	0	0	0	0	0	1	1	0	1	1
**OTU1212**	0	0	0	0	1	0	0	0	0	0	0	0	0	0	0	0	0	1
**OTU1213**	4	0	0	0	0	0	0	0	1	0	0	0	0	0	0	0	0	0
**OTU1214**	0	0	0	0	0	0	0	0	0	0	4	0	0	0	0	0	5	0
**OTU1215**	0	0	0	0	0	0	0	1	1	0	0	0	0	0	0	0	0	1
**OTU1216**	0	0	0	0	0	0	3	8	2	0	0	0	0	0	0	0	1	1
**OTU1217**	0	1	0	0	0	0	0	0	0	0	1	0	0	0	0	0	1	0
**OTU1218**	1	0	0	0	0	0	0	0	0	0	0	0	0	0	2	0	0	0
**OTU1219**	0	0	1	0	2	0	0	0	1	3	3	1	0	1	0	0	1	0
**OTU1220**	0	0	0	0	0	0	36	0	0	1	0	1	0	0	1	0	0	0
**OTU1221**	1	2	1	0	0	0	1	1	0	1	3	0	0	3	0	0	0	0
**OTU1222**	7	1	7	0	0	244	2	2	2	3	2874	1	74	1	1	0	1	1
**OTU1223**	43	79	70	24	27	6	317	209	114	67	110	37	17	45	12	397	382	246
**OTU1224**	0	0	0	0	0	0	0	0	0	0	1	0	0	0	49	0	0	0
**OTU1225**	0	1	0	2	0	0	1	0	0	1	2	1	0	0	0	0	1	0
**OTU1226**	0	0	1	0	0	0	0	0	0	0	0	0	0	0	0	1	0	0
**OTU1227**	1	0	1	0	0	0	1	3	0	0	1	0	0	0	0	2	2	1
**OTU1228**	1	0	0	0	0	0	0	0	0	0	0	0	0	0	0	0	1	0
**OTU1229**	0	0	1	0	0	0	0	0	0	1	5	0	0	1	0	0	0	2
**OTU1230**	47645	57545	10489	2160	11619	14703	35917	38506	24110	2708	11996	45961	8752	2658	3827	459	1446	640
**OTU1231**	0	0	0	0	0	1	0	0	0	0	1	0	0	0	0	0	0	0
**OTU1232**	0	2	0	0	0	0	0	0	0	0	0	0	0	0	0	0	1	0
**OTU1233**	3	10	4	2	2	1	2	5	3	14	11	5	1	2	2	6	4	4
**OTU1234**	65	111	121	30	41	13	52	27	19	126	121	55	14	48	12	32	48	40
**OTU1235**	1	2	1	1	0	0	99	0	0	0	1	1	0	0	0	2	0	0
**OTU1236**	2	0	2	0	0	0	1	0	0	5	2	0	1	1	0	2	1	0
**OTU1237**	0	1	0	0	0	0	0	0	0	1	0	1	0	0	0	0	0	1
**OTU1238**	0	0	0	0	0	0	0	0	0	0	0	1	0	1	0	0	0	0
**OTU1239**	0	0	0	0	1	0	0	0	1	0	1	0	0	0	0	2	0	0
**OTU1240**	4	3	14	0	4	4	0	2	1	5	6	5	1	6	1	10	1	4
**OTU1241**	1	3	0	0	0	0	0	0	0	0	3	1	1	0	2	0	0	1
**OTU1242**	34	80	32	22	13	8	28	23	11	42	57	18	6	20	3	35	43	30
**OTU1243**	1	1	0	0	0	0	1	0	0	0	1	0	1	0	0	1	0	0

**OTU1244**	2	15	12	0	1	0	1	1	1	0	5	7	0	3	0	2	3	0
**OTU1245**	2	5	7	2	1	1	3	1	1	8	13	3	1	5	3	2	2	4
**OTU1246**	1	0	0	0	0	0	1	0	1	0	0	1	0	0	0	0	0	0
**OTU1247**	0	0	0	0	0	0	0	0	0	2	2	0	0	0	0	0	0	0
**OTU1248**	0	0	1	0	1	0	0	0	0	0	0	0	0	0	0	2	1	0
**OTU1249**	36	54	76	13	20	7	59	37	14	76	98	31	17	26	7	38	40	29
**OTU1250**	0	0	1	0	0	1	19	8	2	0	0	0	0	1	0	0	0	0
**OTU1251**	1	0	2	0	0	0	0	0	0	0	1	0	0	0	0	0	0	1
**OTU1252**	1	7	14	3	1	0	2	1	1	8	9	1	0	3	1	10	9	2
**OTU1253**	1	0	2	0	0	0	0	0	0	0	0	0	0	0	0	0	0	0
**OTU1254**	0	0	1	0	0	0	1	0	0	0	0	0	0	0	0	0	0	0
**OTU1255**	0	1	0	0	0	0	0	0	0	0	0	0	0	0	0	0	1	0
**OTU1256**	0	0	0	0	0	0	0	0	0	1	0	0	0	0	2	0	0	0
**OTU1257**	0	0	0	0	0	0	0	0	0	0	0	0	0	0	7	0	1	0
**OTU1258**	0	0	0	0	0	0	0	0	0	1	1	0	0	0	0	0	0	0
**OTU1259**	0	0	0	0	0	0	0	1	0	0	0	0	0	0	0	1	0	2
**OTU1260**	8	14	17	3	2	0	6	5	3	15	13	7	3	6	0	4	4	9
**OTU1261**	7	5	4	1	1	0	14	0	0	2	2	1	0	0	0	1	1	1
**OTU1262**	0	1	2	0	1	0	1	7	0	0	0	0	0	0	0	2	2	0
**OTU1263**	0	0	0	0	0	0	0	0	0	0	2	0	0	0	0	0	0	0
**OTU1264**	0	0	0	0	0	0	1	0	0	0	1	1	0	0	0	0	0	0
**OTU1265**	0	2	0	0	2	0	0	0	0	0	1	0	0	0	0	0	0	0
**OTU1266**	1	0	0	0	0	0	1	0	0	0	1	0	0	0	0	0	0	0
**OTU1267**	2	1	0	0	0	0	1	0	0	0	2	0	0	1	1	0	0	0
**OTU1268**	0	0	1	0	0	0	0	0	0	0	1	0	0	0	0	0	0	0
**OTU1269**	3	4	6	1	2	1	12	0	1	8	12	3	1	4	0	1	2	2
**OTU1270**	2	0	6	0	0	0	0	0	0	9	5	0	0	1	0	0	0	0
**OTU1271**	0	17	3	0	0	0	0	0	0	0	4	0	0	0	0	0	0	0
**OTU1272**	0	0	0	0	0	0	10	7	0	0	1	0	0	0	0	0	0	0
**OTU1273**	2	2	5	2	2	2	4	3	0	2	7	2	0	2	0	1	0	1
**OTU1274**	0	0	2	0	0	0	0	0	0	0	0	0	0	0	1	0	0	0
**OTU1275**	118	117	181	52	54	26	129	49	38	146	227	80	27	71	20	123	112	61
**OTU1276**	17	9	17	3	7	1	8	6	1	16	16	10	4	6	1	9	7	4
**OTU1277**	1628	4131	4422	1565	966	391	2111	1209	826	4591	5703	1421	510	2210	423	2222	2851	1944
**OTU1278**	0	1	0	0	1	0	3	0	0	0	0	0	0	0	0	0	0	0
**OTU1279**	0	6	2	0	1	1	4	1	0	1	4	1	0	2	7	1	0	1
**OTU1280**	1	5	4	3	0	2	0	0	0	2	9	3	0	2	0	1	1	1
**OTU1281**	4	7	15	6	7	0	3	3	1	9	20	3	3	3	1	5	6	1
**OTU1282**	0	0	1	0	1	0	0	0	0	0	0	0	0	0	0	0	0	0

**OTU1283**	0	1	0	2	1	0	0	0	0	0	1	0	0	0	0	0	0	0
**OTU1284**	0	2	2	0	1	2	4	0	1	2	1	7	0	6	0	0	4	0
**OTU1285**	1	0	0	0	0	0	0	0	0	0	0	0	0	0	1	0	0	0
**OTU1286**	0	0	2	0	1	0	3	0	3	1	0	0	0	0	0	0	0	3
**OTU1287**	0	1	1	0	1	0	0	0	1	1	0	0	0	0	0	1	0	1
**OTU1288**	0	0	0	1	1	0	0	0	0	0	0	0	0	0	0	0	0	0
**OTU1289**	12	34	25	13	7	1	14	8	7	32	43	7	9	9	6	9	10	14
**OTU1290**	0	0	1	0	0	0	2	1	0	0	0	0	0	0	0	0	0	1
**OTU1291**	0	0	0	0	2	0	1	0	0	2	0	0	0	0	0	0	0	2
**OTU1292**	56	44	68	21	11	11	31	15	4	44	79	19	9	18	5	29	23	22
**OTU1293**	0	9	0	0	0	0	0	0	0	0	0	0	0	0	1	5	0	0
**OTU1294**	0	0	2	0	0	0	0	0	0	0	0	0	0	0	0	2	0	0
**OTU1295**	4	10	4	2	1	0	3	4	5	12	13	3	0	8	2	7	5	10
**OTU1296**	0	0	1	0	0	0	0	0	0	0	0	0	0	0	0	0	1	0
**OTU1297**	0	1	0	0	0	0	1	1	0	0	0	0	0	0	0	0	0	0
**OTU1298**	337	351	111	1	76	22	106	138	78	20	147	133	82	20	41	1	0	1
**OTU1299**	7	5	11	4	2	0	3	3	3	9	11	1	0	5	0	6	5	7
**OTU1300**	1	0	0	0	0	0	0	0	0	0	1	0	0	0	0	0	0	0
**OTU1301**	1	0	0	0	0	0	0	1	0	0	2	0	0	0	0	0	0	0
**OTU1302**	0	2	0	0	0	0	0	0	0	0	3	1	0	0	0	0	0	2
**OTU1303**	0	0	0	1	0	0	1	0	0	1	0	0	0	0	0	0	0	0
**OTU1304**	4	7	3	3	1	0	5	2	1	4	9	5	2	1	0	3	3	4
**OTU1305**	3	0	1	1	1	0	2	0	0	0	16	12	1	1	2	25	0	11
**OTU1306**	1	6	2	0	1	1	1	0	1	0	1	0	0	1	0	0	0	0
**OTU1307**	6	6	7	0	0	0	4	0	2	6	1	1	0	0	0	2	3	3
**OTU1308**	0	5	0	0	0	0	0	0	0	0	7	0	0	0	0	0	0	0
**OTU1309**	0	0	0	0	0	0	0	0	0	0	1	0	0	0	1	0	0	0
**OTU1310**	0	0	1	0	1	0	3	0	0	0	1	0	0	1	0	2	1	0
**OTU1311**	0	0	0	0	1	0	0	0	0	0	0	0	0	0	0	0	0	0
**OTU1312**	14	12	19	2	4	1	16	36	7	16	21	10	3	15	0	14	5	5
**OTU1313**	0	1	1	0	0	0	0	0	0	0	0	0	0	0	0	0	1	0
**OTU1314**	0	1	0	0	0	1	0	3	2	1	2	2	1	1	0	6	5	1
**OTU1315**	0	0	0	0	0	0	0	0	0	0	6	0	0	0	0	1	0	0
**OTU1316**	1	23	8	1	0	0	1	1	4	3	55	0	1	0	0	1	0	0
**OTU1317**	0	0	0	0	0	0	0	0	0	0	0	0	0	0	0	4	0	0
**OTU1318**	0	3	0	0	0	0	0	0	0	0	1	0	0	0	0	0	0	0
**OTU1319**	3	8	10	2	5	1	6	1	7	16	18	4	3	20	7	8	6	6
**OTU1320**	18	37	30	9	6	5	18	6	5	23	32	13	5	6	3	9	13	15
**OTU1321**	2	2	0	1	0	0	0	0	0	0	0	0	1	0	0	0	0	0

**OTU1322**	4	7	9	2	2	2	9	4	3	10	9	3	2	6	6	5	6	3
**OTU1323**	0	1	0	0	0	0	0	0	0	0	0	0	0	0	0	0	0	0
**OTU1324**	2	2	0	0	0	1	1	0	0	0	0	1	0	0	0	0	0	0
**OTU1325**	1	9	4	0	0	0	0	0	0	0	2	0	0	0	0	0	0	0
**OTU1326**	68	96	91	25	13	13	32	29	13	48	110	43	12	37	16	23	39	35
**OTU1327**	0	0	0	0	0	0	4	2	0	0	0	0	0	0	0	0	0	0
**OTU1328**	0	0	0	0	0	0	1	0	0	0	0	0	0	0	1	0	0	0
**OTU1329**	1	1	0	0	0	0	0	2	0	2	0	0	0	1	0	0	1	0
**OTU1330**	0	0	0	0	0	0	0	0	0	0	0	0	0	0	0	1	0	0
**OTU1331**	5	5	8	0	1	2	2	1	1	1	8	6	1	0	0	1	0	0
**OTU1332**	0	0	1	1	0	0	0	1	0	1	2	0	0	0	0	0	0	1
**OTU1333**	1	0	1	0	0	0	0	0	0	0	0	0	0	0	0	0	0	0
**OTU1334**	0	0	0	1	0	0	0	0	0	1	0	0	0	0	0	0	0	1
**OTU1335**	0	0	1	0	0	0	0	0	0	0	1	0	0	0	0	0	0	0
**OTU1336**	3	0	2	0	0	3	0	0	2	2	2	1	0	0	1	0	0	0
**OTU1337**	0	0	0	0	0	1	0	0	0	0	0	0	0	0	2	0	0	0
**OTU1338**	349	623	824	243	163	90	380	206	130	584	838	297	75	349	78	270	326	235
**OTU1339**	0	1	0	0	0	0	1	0	0	2	2	0	0	0	0	0	0	0
**OTU1340**	1	2	1	0	0	0	1	0	1	2	0	1	0	2	0	0	0	1
**OTU1341**	0	0	0	0	0	0	0	0	0	0	1	0	0	0	0	0	0	0
**OTU1342**	0	0	1	0	0	0	0	0	0	0	3	1	0	0	0	0	0	3
**OTU1343**	0	0	0	0	0	0	0	0	0	0	1	0	0	0	0	0	0	0
**OTU1344**	21	56	34	5	7	3	12	11	2	29	41	4	7	5	5	7	6	4
**OTU1345**	4230	7113	8052	2463	2013	1044	4697	2609	1784	7024	9214	3527	1326	3837	875	3430	4525	3640
**OTU1346**	4	12	13	0	3	1	4	3	1	6	9	4	0	5	1	1	2	2
**OTU1347**	1	1	0	0	0	0	2	0	1	1	0	0	0	0	0	1	0	0
**OTU1348**	1	4	11	3	0	0	5	1	0	2	4	3	1	5	1	1	2	1
**OTU1349**	0	2	0	0	1	0	1	0	1	0	0	0	1	0	0	0	0	0
**OTU1350**	26	1416	442	0	1	2	3	6	0	0	85	4	4	0	0	1	2	0
**OTU1351**	0	1	6	0	0	0	0	0	0	0	0	0	0	0	0	0	0	0
**OTU1352**	5	4	6	1	4	1	1	0	5	9	5	0	1	4	0	2	6	6
**OTU1353**	1	0	0	0	0	0	0	0	0	0	0	0	0	0	3	0	0	0
**OTU1354**	3	3	3	1	3	2	10	8	6	9	11	1	2	3	0	18	13	26
**OTU1355**	2	0	0	0	0	0	0	0	0	0	0	0	0	0	0	0	0	0
**OTU1356**	226	232	8941	10	48	50	156	127	69	1312	10377	172	9459	1007	2736	19	48	28
**OTU1357**	4	0	0	0	0	0	0	1	0	0	1	4	1	0	1	0	0	0
**OTU1358**	0	0	1	0	0	0	0	0	1	1	0	0	0	0	0	0	0	0
**OTU1359**	0	0	0	0	0	0	1	0	0	0	0	0	0	1	0	0	0	0
**OTU1360**	0	0	2	0	0	0	0	0	0	0	0	0	0	0	0	0	0	0

**OTU1361**	5	27	27	2	5	1	8	4	1	8	24	9	4	6	3	1	2	3
**OTU1362**	0	0	0	0	0	0	4	0	0	0	0	0	0	0	0	0	0	0
**OTU1363**	0	0	0	1	0	0	0	0	2	0	0	0	0	0	0	0	0	0
**OTU1364**	0	0	0	0	0	0	0	0	0	1	0	0	0	0	0	0	1	0
**OTU1365**	2	3	2	0	0	0	4	0	0	1	1	0	0	0	0	1	1	1
**OTU1366**	0	0	0	0	0	0	0	0	0	0	4	12	0	1	1	0	0	0
**OTU1367**	0	1	0	0	0	0	0	1	1	0	0	0	0	0	1	0	1	0
**OTU1368**	0	0	0	0	0	0	0	0	0	1	0	0	0	0	0	0	0	0
**OTU1369**	7	7	9	1	0	0	4	1	1	4	8	1	0	5	18	5	3	6
**OTU1370**	0	1	0	0	0	0	0	0	0	0	0	0	0	0	0	0	0	0
**OTU1371**	0	0	3	0	0	0	0	0	0	0	28	0	0	0	0	0	0	0
**OTU1372**	1	0	0	0	0	0	1	0	0	1	0	0	0	0	0	0	1	0
**OTU1373**	1	0	0	0	0	0	1	0	0	1	2	0	0	0	0	0	0	0
**OTU1374**	6	2	5	1	0	0	2	6	3	13	13	1	0	0	2	1	5	3
**OTU1375**	1	2	9	0	0	0	0	0	1	0	3	0	0	0	0	0	2	0
**OTU1376**	282	898	841	275	179	80	371	216	104	720	1009	256	95	400	75	343	462	343
**OTU1377**	2	1	0	0	0	0	1	1	0	0	0	1	1	0	0	0	1	0
**OTU1378**	0	0	0	0	0	0	0	0	0	0	0	0	0	0	0	0	2	0
**OTU1379**	9	19	28	10	5	5	7	11	5	24	35	10	3	13	2	10	12	10
**OTU1380**	0	0	0	0	1	0	3	0	0	0	0	0	0	0	0	0	0	0
**OTU1381**	1	0	2	0	0	0	0	0	0	0	0	0	0	0	0	0	0	0
**OTU1382**	0	16	21	0	0	0	0	0	0	0	0	0	0	0	0	0	0	0
**OTU1383**	0	0	0	0	0	1	0	0	0	0	0	1	0	0	0	0	0	0
**OTU1384**	0	0	0	0	0	0	1	5	0	0	3	0	0	0	0	0	0	0
**OTU1385**	27	88	54	18	16	6	27	6	9	66	108	22	11	17	7	13	27	19
**OTU1386**	0	2	0	0	0	0	0	0	0	0	1	1	0	0	0	0	0	0
**OTU1387**	0	0	0	0	0	0	0	0	0	0	0	0	0	1	0	0	1	0
**OTU1388**	1	1	1	1	0	0	6	3	1	0	1	0	0	0	0	4	5	4
**OTU1389**	0	0	0	0	0	0	0	0	1	0	1	0	0	0	0	0	0	0
**OTU1390**	0	3	0	0	0	0	0	0	0	0	2	1	0	0	0	0	0	0
**OTU1391**	0	1	0	0	0	0	14	5	1	0	0	1	0	0	0	0	0	0
**OTU1392**	0	0	0	0	0	0	0	0	0	0	0	0	1	0	1	0	0	0
**OTU1393**	0	2	0	0	2	0	0	0	0	1	2	0	0	0	1	0	1	1
**OTU1394**	1	15	11	0	0	1	0	0	0	4	0	0	0	0	0	0	0	0
**OTU1395**	0	0	0	0	0	0	2	1	1	0	1	1	0	0	0	0	0	0
**OTU1396**	0	0	1	1	0	0	0	0	0	0	0	0	0	0	0	0	0	0
**OTU1397**	0	0	0	0	0	0	0	0	0	1	0	0	0	0	0	0	1	0
**OTU1398**	0	0	0	0	0	0	0	0	0	1	0	0	0	0	1	0	0	0
**OTU1399**	0	2	0	0	0	0	1	0	0	0	0	0	0	0	1	1	1	0

**OTU1400**	0	0	0	0	0	0	0	0	0	0	1	1	0	0	0	0	0	0
**OTU1401**	3	22	0	1	2	0	0	0	6	3	3	0	0	2	5	12	4	0
**OTU1402**	0	1	0	0	0	0	0	0	0	0	1	0	0	0	0	0	0	0
